# *De novo* computational identification of stress-related sequence motifs and microRNA target sites in untranslated regions of a plant translatome

**DOI:** 10.1038/srep43861

**Published:** 2017-03-09

**Authors:** Prabhakaran Munusamy, Yevgen Zolotarov, Louis-Valentin Meteignier, Peter Moffett, Martina V. Strömvik

**Affiliations:** 1 Department of Plant Science, McGill University, Sainte-Anne-de-Bellevue, Québec, H9X 3V9, Canada; 2 Department of Biology, Université de Sherbrooke, Sherbrooke, Québec, J1K 2R1, Canada

## Abstract

Gene regulation at the transcriptional and translational level leads to diversity in phenotypes and function in organisms. Regulatory DNA or RNA sequence motifs adjacent to the gene coding sequence act as binding sites for proteins that in turn enable or disable expression of the gene. Whereas the known DNA and RNA binding proteins range in the thousands, only a few motifs have been examined. In this study, we have predicted putative regulatory motifs in groups of untranslated regions from genes regulated at the translational level in *Arabidopsis thaliana* under normal and stressed conditions. The test group of sequences was divided into random subgroups and subjected to three *de novo* motif finding algorithms (Seeder, Weeder and MEME). In addition to identifying sequence motifs, using an *in silico* tool we have predicted microRNA target sites in the 3′ UTRs of the translationally regulated genes, as well as identified upstream open reading frames located in the 5′ UTRs. Our bioinformatics strategy and the knowledge generated contribute to understanding gene regulation during stress, and can be applied to disease and stress resistant plant development.

Precise regulation of gene expression is important for plants to survive environmental variations. Plant cells have to synthesize proteins in response to biotic and abiotic stresses. Studies have revealed that the level of mRNA transcript produced does not always correlate to the protein synthesized, which could be attributed to variable mRNA translation efficiency^1^. Several features of mRNA influence the translation activity, most importantly regulatory elements in the 5′ and 3′ untranslated regions such as a 5′ methyl cap and a 3′ poly-A tail, which have both been found to play a significant role in regulating gene expression at the translational level^2^,^3^,^4^. Other influencing features include the length of 5′ and 3′ UTRs, secondary structure, presence of start codon and upstream open reading frames (uORFs)^5^,^6^. A number of studies have been carried out to understand the translational mechanism occurring in plants in response to stress. It has been observed that ribosome loading of mRNAs is affected globally under abiotic stresses such as salt, drought, hypoxia, light and darkness^7^,^8^. Some mRNAs also seem to escape from the translational block and are thus regulated during stress. For example, under heat stress in plants, mRNAs encoding heat shock proteins were found to be highly regulated whereas some mRNAs bound to heat shock granule complexes were translationally repressed^9^. Follow-up studies^10^,^11^ identified a sequence element located in the 5′ UTR responsible for active mRNA translation under heat shock.

Plant miRNAs are known to trigger mRNA cleavage or translational repression by binding to target sites found in the 5′ or 3′ UTR, and protein coding regions^12^,^13^. Studies have identified various roles of miRNAs in plant growth and development, and its response to stress^14^,^15^. For example, an Arabidopsis SPL3 gene encoding an SBP-box transcription factor is translationally regulated by miR156/miR157, which inhibits SPL3 gene expression by binding to its target complementary site in the 3′ UTR leading to early flowering phenotype^16^. In addition to miRNAs, UTRs have regulatory motifs or sequence patterns to which regulatory RNA binding proteins bind and mediate translational control. For example, Bruno-like proteins encoded by the *AtBRN1* and *AtBRN2* genes in *Arabidopsis thaliana* led to delayed flowering time. In another study, it was discovered that a Bruno-like protein binds to a sequence element in the 3′ UTR of *SOC1* mRNA and represses its expression, thereby causing delayed flowering time^17^. However, understanding the control and regulation of translational processes is still at its early stage. Most of the evidence on translational control of genes is provided by mutant and genetic screening studies, and very little work has been carried out at the high-throughput sequence level to identify RNA control elements.

In this study we set out to use various bioinformatics approaches and different computational tools to analyze genes that are translationally regulated under stress in Arabidopsis for various regulatory elements potentially responsible for mediating translational regulation.

## Results

### Identification of stress-regulated genes at the translational level

In order to study gene regulation at the translational level during stress, differential expression analysis was carried out on RNA-Seq data of total and ribosome-bound mRNAs from control and dexamethasone treated *Arabidopsis thaliana* plants. Assuming that a transcriptionally regulated gene would have equal levels or abundance in the transcriptome and the translatome, the definition of a translationally regulated gene would be a gene for which the level or abundance in the transcriptome differ from the level or abundance in the translatome.

The differentially expressed genes were classified into six different groups depending on them being likely transcriptionally or translationally regulated in control and/or treated plants (representing stress), and whether this regulation was up (more transcripts) or down (fewer transcripts). The YNup and YNdown groups contain genes that are translationally up-regulated and down-regulated respectively in treated plants (i.e. under stress) but are normally regulated (transcriptionally) in control plants. The NYup and NYdown groups contain genes with significant translational up-regulation and down-regulation respectively in control plants but with normal regulation (transcriptional) in treated plants. The YYup and YYdown groups contain genes that are up-regulated and down-regulated respectively at the translational level in both control and treated plants. Based on the Cuffdiff derived FPKM values, in total 514 genes (Supplementary Table 1) are noticeably regulated at the translational level during stress, spread between 12–265 genes depending on the group (Table 1). About half of the genes (265/514 or 51.5%) were in the NYdown group, whereas the lowest number of genes were in the YYup group, with only 12 genes.

Since the untranslated regions (UTRs) of genes are of great importance to the ability of a transcript to be translated, the UTRs of the six different groups of genes were investigated for potential conserved sequences, such as regulatory elements, miRNA target motifs and upstream open reading frames (uORFs). Genes with 3′ and 5′UTR sequences longer than 10 nucleotides were selected using a custom written Python script. The resulting number of genes with 5′ and 3′ UTR sequences for each group is shown in Table 1.

### Presence of uORFs in the 5′ UTR of stress-regulated genes

Upstream open reading frames (uORFs) are thought to influence translation of downstream protein coding regions of a gene. Therefore, the 5′ UTRs of the genes in the set of translationally regulated genes were searched for open reading frames (ORFs) using the UTRscan program, which finds matches to experimentally verified or predicted uORFs from literature (see Pesole, G., *et al*.^5^). The results show that approximately 19% (89/455) of the genes contain one or more uORFs. In total, 106 uORFs were identified from 89 genes exhibiting significant translational regulation during stress (Table 2). The size of the putative uORFs identified in the data ranges between 66 nucleotides (or 22 codons) to 231 nucleotides (or 77 codons), with an average of 105 nucleotides (or 35 codons). Out of the 106 uORFs, 65 (or 73%) were located 20 base pairs away from the start (5′ end) of the 5′ UTR sequence, which could possibly be regulating the translational process of the genes containing them. The full list of uORFs are detailed in Supplementary Table 2. Among the genes predicted to have uORFs in the 5′ UTR, some of them were identified to contain multiple uORFs (Table 3). We hypothesized that the number of genes containing uORFs would be higher in the set of translationally regulated genes than in the whole transcriptome. However, based on a statistical z-test with p-value < 0.05, the prevalence of uORFs (89 out of 455 genes) were significantly lower in the set of translationally regulated genes when compared to the total Arabidopsis transcriptome (8,234 uORFs present in 6,089 out of 19,128 genes), and therefore the hypothesis was rejected.

### Start codon sequence context of the predicted uORFs

**Figure 1:**
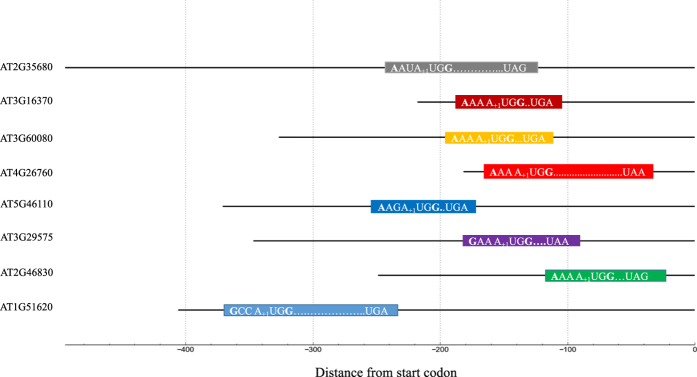
Position of upstream open reading frames (uORFs) possessing a strong Kozak sequence context in plant genes translationally regulated under stress. The Kozak signal is the nucleotides [A/G] at position −3 and G at +4, where A of the uORF AUG codon is designated +1. 0 indicates the start of the main protein coding sequence.

Previous studies have shown that the nucleotides around the start (AUG) codon of the uORFs play a major role in determining the translation efficiency of the uORF and its effect on the main coding region in the mRNA^18^,^19^. In particular, the A(A/G)CCAUGGC sequence called a Kozak signal seems to be of importance. Therefore, the sequences surrounding the start codons of the predicted uORFs were analyzed. As seen in Fig. 1, eight uORFs with strong sequence context were identified around the start AUG codon with nucleotides ‘A/G’ at −3 and ‘G’ at +4 positions (A of AUG codon is marked with +1 in the figure). In addition, approximately 18% of the identified uORFs were found to have a sub-optimal sequence context around its AUG codon, which could possibly be recognized by the ribosome under some specific conditions and initiate the translation process.

### MicroRNA target sites located in the 3′ UTR

Several studies have demonstrated the activity of miRNA in plant gene expression during plant stress and development processes. In this study, miRNA targets were analyzed in the 3′ UTRs of genes predicted to be translationally regulated under stress, using the psRNAtarget web server^20^. In total, 26 miRNA target sites were predicted in the down-regulated groups (YNdown, NYdown and YYdown) and 5 target sites were identified in the up-regulated NYup group (Table 4). The genes in the YNup and YYup groups do not have any miRNA target sites in their 3′ UTRs. Specifically, the number of miRNA target sites identified in each group were 22, 2, 2 and 5 in the NYdown, YNdown, YYdown and NYup, respectively. Based on this analysis, approximately 8% (26 out of 316) of the translationally down-regulated genes have miRNA target sites, as do around 3% (5 out of 154) of the translationally up-regulated genes. The presence of more miRNA binding sites in the 3′ UTRs of down-regulated genes is consistent with the role of miRNA; i.e., they either suppress or inhibit gene expression.

Most of the identified target sites match perfectly with complementary regions of miRNAs. The least mismatch is ≤3 base pairs between them. Importantly, the mismatches are present at either end of the miRNA complementary region, and very rarely at the central region between 9–11 nucleotides, which determines the activity of the miRNA in either cleaving or inhibiting the mRNA expression.

Genes AT4G12080, AT1G53160 and AT2G03750 had target sites for the miR156/miR157 in their 3′ UTRs. The AT1G53160 encodes the transcription factor SPL (SQUAMOSA promoter binding protein-like), which is involved in the regulation of flowering specifically during the floral induction in plants. This gene has been identified in our analysis, and from previous studies, to be regulated by the miR156^21^,^22^.

**Figure 2:**
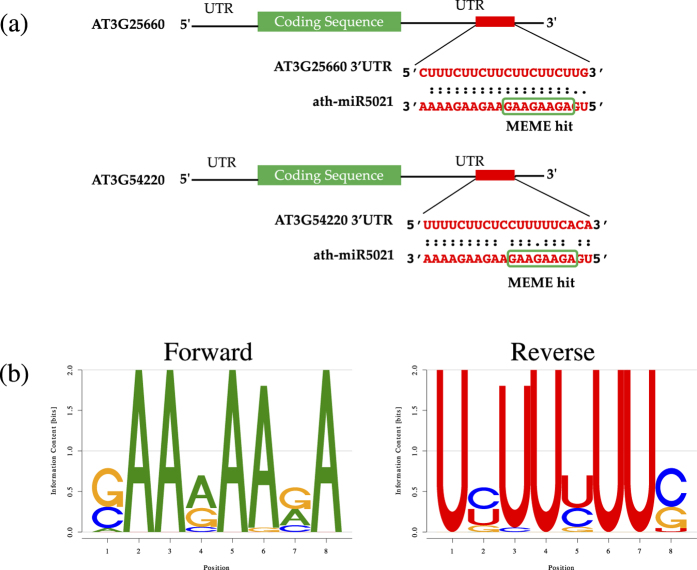
Two genes in the group predicted to be translationally down-regulated under stress were identified in the analysis to contain a binding site for the microRNA (ath-miR5021) in the 3′UTR. The psRNA target web server was used to identify microRNA target sites in translationally regulated genes. (**a**) Gene AT3G25660 has three nucleotide mismatches present on either end of their binding region which could lead to miRNA cleavage activity; whereas the gene AT3G54220 has a nucleotide mismatch in the central region of the binding site possibly leading to mRNA translation inhibition. (**b**) In addition, a conserved sequence motif discovered by MEME was found within the miRNA binding region. The motif is present between 2–11 nucleotides from the 5′ end of the miRNA, which is known as the seed region - important for miRNA activity. This motif was conserved in the 3′ UTR of 26 out of 54 genes that are translationally regulated under stress.

The AT3G25660 gene encodes an amidase family protein, and it is among those predicted to be translationally down-regulated under the impact of stress response in our Arabidopsis transgenic plants. It has a miRNA binding region in its 3′ UTR between 134–153 bp for miR5021 (Fig. 2). Analyzing the 5′ and 3′ end around the binding site of the miRNA (miR5021) revealed a complete and a partial mismatch at either end of the complement, and a full complementarity in the central region. Interestingly, another gene, AT3G54220, a basic-leucine zipper domain containing protein, functioning similar to a DNA binding protein involved in root radial organization and leaf development, was predicted to have a target site for the miR5021 in the region 1–20 of the 3′ UTR. Analyzing its complementary binding region, a nucleotide mismatch was identified in the middle region of the mRNA-miRNA duplex (Fig. 2a).

### Genes regulated at the translational level contain regulatory motifs

In order to understand the mechanism of gene regulation at the translational level, putative regulatory motifs were predicted in the 5′ and 3′ UTRs of the mRNA. The approach was to divide the set of sequences into random subgroups and analyze those with the three *de novo* motif discovery tools Seeder, Weeder and MEME using the untranslated sequences of the whole Arabidopsis genome as the background distribution model. Based on the statistical threshold cut-off value such as Q-value ≤ 0.05 for Seeder, E-value ≤ 0.05 for MEME and top significant motifs produced by the Weeder adviser, a number of significant motifs were found in the 5′ and 3′ UTRs, detailed below.

### Motifs identified in the 3′ and 5′ UTR

The random subgroups containing the 3′ and 5′ UTR sequences were analyzed for the presence of motifs of length 6 and 8. Several motifs were detected with high significant cut-off value in the 3′ UTRs. Seeder identified over-represented 6- and 8-mer sequence motifs among the subgroups of genes in the YNup, YNdown, NYup, NYdown and YYdown groups, but not in the YYup group. MEME identified 6-mers only in subgroups of the NYdown and NYup groups, 8-mers in the NYdown, NYup and YNdown groups, and no motifs in the YNup, YYup and YYdown groups. Weeder detected 8-mers in all six groups and 6-mers in three of the six groups (YNup, YYdown and YYup). The exact number of significant motifs detected in each group is shown in Tables 5 and 6.

The number of 6- and 8-mer motifs discovered in the 5′ UTRs also varied between the three different tools used (Tables 5 and 6). The 8-mer motifs were detected in almost all the groups by the three motif prediction tools except that motifs were not identified by MEME in the YYup group. In the prediction of 6-mers, significant motifs were found in five out of six groups by Seeder and MEME, and none were identified in the YYup group. Finally, Weeder was able to produce top significant 6-mer motifs for the groups YNup, YYdown and YYup, but no over-represented motifs were predicted in the NYdown, NYup and YNdown groups.

Many identified motifs are similar to each other within the same group. We carried out a pairwise comparison analysis using the position weight matrices of the motifs to determine their similarity. Using the Tomtom tool^23^ for pairwise comparison, based on a threshold cut-off value of 0.05, the motifs that are similar were clustered to produce an average position weight matrix containing the information content of the nucleotides from each and every matrix combined into one. The clustered average matrix was then used to find genes having those motifs in the individual dataset as well as in the whole Arabidopsis genome.

In order to predict the function of the discovered motifs, the literature was searched to find similar motifs identified in the UTRs. As a result, two motifs relevant for 3′ UTRs and one for 5′ UTRs were identified that are experimentally tested and found to play a significant role in gene regulation at the translational level. The motifs in the 3′ UTR are [UGUA (A/C/U) AUA], a Pumilio protein binding motif 24 and [U(G/A)U(A/G)U(G/A)U], Bruno-like protein binding motif^17^; and the motif in the 5′ UTR is [UAGGGUUU]^25^. Position weight matrices were made of these three motifs and used to compare against the motifs discovered in the study. As a result, 23 motifs identified from the 3′ UTRs were found to match the binding motifs of Pumilio and Bruno-like protein (Supplemental Tables 3–8). Examples of genes containing these two motifs are mentioned in Tables 7 and 8. Likewise, 7 motifs from the 5′ UTR matched to the single motif [UAGGGUUU] identified from literature (Supplemental Tables 9–14). For instance, motif S29 identified by Seeder in the 5′ UTR matched to the reverse complement of the motif [UAGGGUUU]. Motif S29, predicted from the NYup group, was found to be present in 12 out of 79 genes in the specific group. Among the genes of interest containing the S29 motif, gene AT4G27310, which encodes a B-box type zinc finger family protein possessing DNA binding transcription factor activity, is involved in transcription regulation^26^ whereas genes AT2G07706 and AT1G20970 encode an uncharacterized protein. Gene AT5G59880 encodes actin depolymerizing factor 3 (ADF3), which is known to play a role in several biological processes such as depolymerisation of actin, gluconeogenesis and stress response^27^,^28^. Another gene, AT1G10940 encodes a protein kinase similar to the calcium/calmodulin-dependent protein kinase subfamily and the SNF1 kinase subfamily (SnRK2), which is involved in plant response to stress^29^.

In addition, we tested if any motifs discovered in this study matched motifs identified in data from 24 different eukaryotic species comprising 244 RNA binding motifs (of which six were from plants) recognized by 205 RNA binding proteins (see Ray *et al*.)^30^. Approximately 10–12% of the motifs, i.e., 19 out of 193 and 32 out of 276 motifs identified from the 3′ UTR and 5′ UTRs of translationally regulated genes, had a match within the set of 244 motifs. The matches are noted in Supplementary Tables 3–14. Some of the matches were to motifs already identified as matches to Pumilio and or Bruno motifs (e.g. the motifs RNCMPT00011/(PAPI), RNCMPT00166, (BRUNOL5), RNCMPT00003/(ARET), RNCMPT00270/(ARET), RNCMPT00004/(BRUNOL4) all match our CACACAA motif (S19)), while other matches were to unannotated motifs.

### A conserved sequence motif identified using MEME found in the down-regulated set of genes present in the miRNA binding site

Using a *de novo* motif discovery tool, MEME, a motif (Fig. 2b) was discovered in 26 genes among the set of 54 down-regulated genes in the 3′ UTR identified based on FPKM values in plants under stress. Interestingly, this motif is in the miRNA target site of the two genes that were predicted to bind to ath-miR5021. In addition, this motif is present in the 5′ end region of the miRNA, which is highly responsible for miRNA recognition of the target mRNA, called a seed region (2–11 of 20 nucleotides) (Fig. 2a). It is possible that other genes containing this motif might also be regulated by miRNAs except that it was not predicted in our analysis for reasons such as the complementarity, and the un-pairing energy optimal threshold value^31^ used to predict miRNA sites might vary in the *in vivo* conditions of plants.

## Discussion

### Upstream ORFs located in the 5′ UTRs may be involved in translational regulation

In order to understand the role of uORFs during gene translation under stress, several uORFs were detected in the 5′ UTR of translationally regulated genes expressed under stress. Studies have reported that sequences around the AUG start codon of the uORF play an important role in promoting translation initiation in plants^32^,^33^. There is a high probability for the uORFs with a strong Kozak signal to be translated upon ribosome recognition whereas the uORFs with weak or sub-optimal nucleotide context might get translated under certain specific conditions in plants; however, the exact mechanism of uORF start codon recognition by the ribosome is not yet clear^34^. Evidence from several studies show that the uORFs position in the 5′ UTR, among other factors, has a great impact on its functional role. Out of the 106 predicted uORFs, 65 were located 20 nucleotides away from the start of the 5′ UTR. Studies have shown this to be the optimal distance for uORF translation initiation to occur^35^. It is also to be noted that these 65 uORFs are positioned relatively at a distance of approximately 20 nucleotides from the downstream main ORF^36^,^37^.

Based on the factors that determine uORF functionality, some of the predicted uORFs could act in a sequence-dependent manner. One of the translationally regulated genes with a detected uORF, AT2G46830 encodes circadian clock associated 1 (CCA1), a transcription factor protein involved in regulating the circadian system of Arabidopsis, and is required for sensing changing environmental conditions such as light and temperature^38^. AT2G46830 contains an uORF between positions 147–232 in the 249 nt long 5′ UTR. The uORF of this gene has a strong Kozak sequence required by the ribosome for translation initiation. In addition, this uORF is located a few nucleotides away from the main ORF, which could result in translation of the uORF after which a translation reinitiation process might occur to translate the main ORF located downstream of it. From the differential gene expression analysis, this gene is predicted to be down-regulated at the translational level during stress. Therefore, it is highly likely that the uORF upon translation would either produce a peptide and in turn stall the ribosome from further scanning thereby inhibiting the main ORF translation or the ribosome after uORF translation initiation would encounter a pre-termination codon triggering a non-sense mediated decay process. Studies show that reduced levels or loss of CCA1 function in Arabidopsis affects various developmental processes regulated by light, prompts change in flowering time and disrupts the function of circadian clock and its related gene expression^39^,^40^.

Two other genes of interest are AT1G74088, which encode galacturonosyltransferase enzyme, and AT1G54260, coding for winged-helix DNA-binding transcription factor family protein involved in nucleosome assembly^41^. These two genes are among the YNdown genes, and they have three and two uORFs, respectively. It is possible that the expression of these two genes might be inhibited by the presence of more than one uORFs through stalling the ribosome from scanning the mRNA transcript. In addition, the time required to transverse the 5′ UTR of these genes by the scanning ribosome would be very long. Thus, it is possible that translation of the main open reading frame of those two genes would be inhibited in the process under stress conditions. In a similar manner, gene AT4G02280, that belongs to the NYdown group, had two uORFs in its 5′ UTR. AT4G02280 encodes a sucrose synthase enzyme 3 involved in processes such as starch metabolism, sucrose biosynthesis and metabolism, and studies have demonstrated the requirement of sucrose synthase activity during hypoxia and water deprivation conditions in plants^42^. Additional genes of interest from the up-regulated groups, NYup and YNup, such as AT3G29575 and AT1G51620, contain two uORFs. AT3G29575 encodes an ABI five binding protein 3 (AFP3) and studies have discovered that ABI five related proteins play a major role in abscisic acid signalling and in various stress responses to modulate the seedling development and growth where it is highly expressed^43^,^44^. Gene AT1G51629, a protein kinase family protein, has been discovered in studies to be highly regulated during resistance against bacterial *P . syringae* infection on plants^45^. As these two genes AT3G29575 and AT1G51620 are found to play a significant role during stress, the possible mechanism that could occur with the presence of uORFs in their 5′ UTR is that under stress the ribosome might by-pass without recognizing the uORF start codon through a leaky scanning mechanism and may directly translate the main ORFs.

### The 3′ UTRs of translationally regulated genes contain microRNA targets

Several miRNA prediction tools are available for animal data^46^,^47^. For plant miRNA prediction, however, only a few are available. Using the psRNAtarget server, several miRNA targets were identified in the 3′ UTRs of genes regulated at the translational level during stress. Genes specifically down-regulated during stress contained predominantly higher numbers of microRNA target sites compared to the up-regulated genes.

It is evident from the mechanism of miRNAs action on the targets that these miRNAs, upon binding to the target site in the 3′ UTR, would recruit the argonaute proteins and they in turn either cleave the mRNA using an RNA-induced silencing complex mechanism or translationally repress the activity of the mRNA by sequestering to it^48^, based on the complementarity around the target regions between the miRNA and the mRNA target.

As indicated in the results, gene AT3G25660 of YNdown group was predicted to bind to ath-miR5021 with complete complementarity in the central region of the binding site. Studies have discovered^31^,^49^ that perfect complementarity in the middle of the miRNA-target duplex would allow access for the Argonaute proteins and RISC complex to bind and cleave the mRNA whereas in the case of gene AT3G54220, a basic-leucine zipper domain containing protein, a mismatching nucleotide base pair in the middle region of the miRNA binding site for the same microRNA (ath-miR5021) was revealed, which could lead to a bulge formation. Therefore, the miRNA would only be able to repress the translational activity as the RISC complex would be blocked from interacting with the target. Thus, these two genes, AT3G25660 and AT3G54220, with a miRNA binding site at the 3′ UTR, have a very good chance of being translationally repressed by miRNAs in the event of stress induction in plants.

### Highly conserved sequence motifs in the UTR

A motif search in the untranslated regions of translationally regulated genes yielded motifs with consensus sequences that were rich in [GA] and [CT] nucleotide repeats both in the 5′ and 3′ UTRs. Studies suggest that single/di-nucleotide repeats plays a role at the transcriptional as well as at the translational level^50^,^51^,^52^,^53^. Important factors such as the location of the repeat and the nucleotide content of the UTR has been observed to affect its functionality and very little has been studied on sequence repeats in plants^54^. For example, a CAG repeat located in the 5′ UTR of the human calmodulin-1 (hCALM1) gene when disrupted has been observed to reduce its expression level significantly^55^. BASIC PENTACYSTEINE1 (BPC1), a regulatory protein, is known to bind the *Arabidopsis thaliana* gene SEEDSTICK (STK) and regulate the ovule identity in Arabidopsis. In a study conducted on a bpc1 mutant an increased STK expression was observed and it was revealed in the study that BPC1 induces conformational change to the STK gene upon binding to the GA repeats in wild-type plants (see Kooiker, M., *et al*.^50^). It is possible that the predicted sequence motifs containing di-nucleotide repeats may be involved in translational regulation but experimental validation is needed for confirmation.

### Motifs similar to Pumilio and Bruno-like protein motifs found in our analysis

Based on the *de novo* motif discovery analysis of untranslated regions (UTRs) of genes regulated at the translational level during (simulated) defense, using our approach of dividing the set of sequences into random subgroups, a number of significant regulatory motifs have been identified in the 3′ UTRs using the Seeder software. Among the several significant motifs discovered, we found a few motifs that match a sequence/*cis*-regulatory element that was experimentally characterized and validated in two different studies (see Kim, H. S., *et al*.^17^ and Huh, S. U., *et al*.^24^). In a study conducted in Arabidopsis, a Bruno-like protein was found to bind a motif [U(G/A)U(A/G)U(G/A)U] located in the 3′ UTR of SOC1 (SUPPRESSOR OF OVEREXPRESSION OF CONSTANS1) mRNA, which encodes a MADS box transcription factor, which modulates the flowering time in plants (see Kim, H. S., *et al*.^17^). In another study, during viral infection on Arabidopsis, a *cis*-regulatory element [UGUA (A/C/U) AUA] in the 3′ UTR of viral RNA interacts with the Pumilio protein, which suppresses the viral infection in plants (see Huh, S. U., *et al*.^24^). Interestingly, both the motifs identified in the literature are highly similar to each other but are recognized by two different proteins under different conditions. Out of the 54 genes in our dataset of translationally down-regulated (YNdown) genes, 18 genes had motifs in their 3′ UTR similar to the ones recognized by the Bruno-like protein (noted in Table 7). As mentioned previously, the Bruno-like protein is involved in translational down-regulation of genes responsible for the regulation of flowering time by binding to the *cis*-regulatory element in their 3′ UTR. Mounting evidence has shown that altering flowering time when under stress is an evolutionary strategy to maximize the chances of reproduction^56^, and it is possible that the genes in our study with motif binding sites for the Bruno-like protein are down-regulated under stress, which might in turn affect the flowering time. With this evidence, and from our bioinformatics analysis findings, there is a high probability that the regulatory motifs located in the 3′ UTR in our set of genes might be responsible for gene down-regulation at the translational level. Likewise, in the same dataset, we discovered 8 genes (noted in Table 8) containing the motif recognized by the Pumilio protein in Arabidopsis, which could be involved in translational control of the genes. Overall, the down-regulation of genes during defense might be associated with the Pumilio and Bruno-like protein motif in their 3′ UTRs. About 10% of the discovered motifs in this study, including some of the motifs matching Pumilio and Bruno-like protein motifs, also matched motifs previously described^30^, adding further strength to the results of our study. These motifs are candidates for a future study on the molecular mechanisms of translational regulation in plants under stress. Since our data is restricted to leaf under stress condition, it is likely that other motifs and more matches would be detected in additional data representing the translatome under different developmental stages, different tissues and different stresses.

## Conclusion

The role of untranslated regions in post-transcriptional or translational control of plant genes is not thoroughly known. Experimentally, it is difficult and tedious to find the specific regulatory elements involved in gene translational regulation. Computational methods can help form hypotheses and make experimental validation more targeted and precise. In this study, we used various computational methods to predict statistically significant regulatory elements in UTRs of genes that are likely to be translationally regulated.

In the first objective of our study, for the prediction of *de novo* motifs in the untranslated regions (5′ and 3′ UTR), we developed a novel bioinformatics approach to elucidate the conserved sequence motifs in our genes of interest. The objective was carried out on genes that are differentially regulated at the translational level in plants under stress. As a result, several over-represented motifs were identified in the 5′ and 3′ UTRs. Interestingly, a higher number of conserved and distinct motifs were discovered using our approach. Some of the motifs predicted matched to the experimentally validated motifs, Pumilio and Bruno-like protein binding motifs, which could be potentially involved in the translational regulation of genes.

For the second objective, the computational tool psRNAtarget was used to predict the miRNA target sites in the 3′ UTRs. The Arabidopsis miRNAs publicly available in the miRBase database were used to search for miRNA complementary sites in the 3′ UTRs of our genes. As a result, several miRNA target sites were predicted and most of them were found in the translationally down-regulated genes of our dataset. It is very highly likely that these genes with miRNA target sites could be down-regulated as the miRNA upon binding to the target either cleaves the mRNA transcript or represses its translational activity.

An interesting finding in this study is that one of the discovered miRNA target sites was also identified in our *de novo* motif discovery analysis as a statistically significant overrepresented motif. Studies suggest that the seed region is of high importance for the miRNA binding and correct function and, based on us finding it in the YNdown group, this suggestion appears to be accurate. Two genes that were predicted to be down-regulated at the translational level, and that contain the ath-miR5021 miRNA target site in their 3′ UTR and a conserved motif in the miRNA binding region, are currently being tested for the miRNA activity in a transient transformation experiment.

In addition to miRNA and regulatory motif identification, the 5′ UTRs were analyzed for uORFs, which have been demonstrated to play a significant role in translation initiation. We have identified several uORFs, some with a strong Kozak sequence context necessary for ribosome recognition, and they are located at an optimal distance that could facilitate their translation. Some of the genes with uORFs in their 5′ UTR also had miRNA target sites in the 3′ UTR. It is possible, depending on the specific condition, either one of these two regulatory elements could be involved in gene translation.

In conclusion, our bioinformatics approach for motif identification has detected several significant motifs, and few of them were found to match the experimentally verified motifs. The regulatory elements such as miRNAs, uORFs as well as the regulatory motifs predicted in this study need to be further validated using experimental techniques in the lab to determine their exact role(s) in translation of genes under stress.

## Materials and Methods

### Source of transcriptome and translatome data

Transcriptome and translatome data were generated from transgenic *Arabidopsis thaliana* plants containing a dexamethasone (DEX) inducible promoter::*Avrpm1* protein construct and rpl18-FLAG^57^,^58^,^59^. Briefly, when the construct is induced (by DEX), the Avrpm1 protein is expressed and recognized by the Rpm1 protein, which leads to a rapid defense response in plants. The rpl18-FLAG expresses an epitope tagged version of the rpl18 ribosomal protein, which was used to purify mRNAs bound to ribosomes. This mRNA pool thus represents the translatome. Plants were subjected to two hours of treatment in the presence or absence (control) of DEX. Total RNA and ribosome-bound mRNAs were purified and RNAseq libraries were prepared^60^. The four different RNA samples (total RNA from DEX treated plants; ribosome-bound mRNA from DEX treated plants; total RNA from control plants; ribosome-bound mRNA from control plants) were sequenced using the Illumina HiSeq2000 sequencing platform. The data discussed in this publication have been deposited in NCBI’s Gene Expression Omnibus^61^ and are accessible through GEO Series accession number GSE75640. Raw reads were mapped against the TAIR10 assembly of the Arabidopsis genome^62^ using Bowtie2 v2.1.0^63^. Using samtoolsv1.4^64^, reads with a mapq score >10 were kept for subsequent analysis. Using Cuffdiff^65^, differential gene expression values were calculated between DEX treated and control samples at the transcriptome (total RNA purification) and translatome (ribosome-bound mRNAs) levels. The translational efficiency of each gene was determined by calculating the ratio of translatome FPKM over transcriptome FPKM in control and DEX conditions.

An assumption is that under normal gene regulation, the transcriptome level of a gene would be equal to the level of that gene in the translatome (ribosome-bound mRNAs), that is, a ratio of 1. A gene is labelled ‘YES’ (Y) when that gene has a translational efficiency significantly different from 1, (transcriptome over translatome ratio) as calculated using Cuffdiff. A label ‘NO’ (N) is given to the gene if it is normally translated (ratio not significantly different from 1) based on its translational efficiency (transcriptome over translatome ratio). To investigate if any gene is under translational control in treatment or control plants, based on the logarithmic ratio of translational efficiency, each gene is denoted with YES-NO (YN), NO-YES (NY) or YES-YES (YY) designation. As per the nomenclature and based on the log ratio of translational activity, the three main groups or lists of genes of interest are: YES-NO (YN)—genes that possess significantly different translational efficiency under defense response (DEX treatment) but a normal translational efficiency in control plants; NO-YES (NY)—genes that possess a normal translational efficiency under defense response (DEX treatment) but a significantly different translational efficiency in control plants; and YES-YES (YY)—genes that show a translational efficiency significantly different from 1 in both treatments. Furthermore, the genes in each group were also identified as up- or down-regulated based on the comparison of their log ratio of transcript abundance at the translational level in control to the defense plants versus the log ratio of transcript abundance at the transcriptional level. In total there are thus six lists of genes: YNup, YNdown, NYup, NYdown, YYup and YYdown.

### Sequence retrieval

For each of the six gene lists in the dataset, using the gene identifiers (e.g., AT3G54220), the 5′ and 3′ UTR FASTA sequences of *Arabidopsis thaliana* were retrieved using the BioMart tool from Phytozome (http://www.phytozome.net/)^66^. Genes with no sequence content and those with very short sequences (<10 nucleotide) were removed using a custom written Python script, available through GitHub: https://github.com/prabhakaranm/UTR_Regulatory_elements.

### Prediction of uORFs

The 5′ UTR sequences of 463 genes differentially regulated at the translational level in *A . thaliana* under stress conditions were used for upstream open reading frame (uORF) analysis. The sequences in FASTA format were subjected to open reading frame (ORF) prediction using the UTRscan tool with default parameters (http://itbtools.ba.itb.cnr.it/utrscan) resulting in an output file with the ORF sequence and its position in the 5′ UTR of the genes. UTRscan utilizing PatSearch^67^, a pattern matching program searches the input sequences for matches to predefined patterns or motifs from the UTRsite database, which is built based on experimental evidence and literature reports concerning ORFs. The database contains 473,330 5′ UTR and 527,323 3′ UTR entries of eukaryotic mRNAs, obtained from 483,605 genes across 79 species^68^.

### Prediction of microRNA target sites

The miRNA target sites in the 3′ UTRs of translationally regulated genes were predicted using a plant small RNA target analysis (psRNATarget) server^20^ (http://plantgrn.noble.org/psRNATarget/). The 3′ UTR sequences of genes with potential translational activity were uploaded to the psRNATarget server and compared with the 337 published miRNAs of *Arabidopsis thaliana* from the miRNABase^69^ already available at the server. The analysis was run with default parameters using a score of 3.0 for maximum expectation (mismatch value accepted), a length of 20 bp for complementarity scoring and an un-pairing energy threshold value of 25.0. A range from 9 to 11 nt was set to find any mismatch in the central region, which would predict the activity of the miRNA.

### *De novo* motif discovery in the 3′ and 5′ UTRs

To discover motifs we used three different *de novo* motif discovery tools, Seeder, Weeder and MEME, run with their default parameters tolerating 5% false discovery rate. The 3′ UTR sequences (20,346) from the whole *Arabidopsis thaliana* genome were downloaded from Phytozome. The Seeder::Background module was used to generate the background distribution of seed length 6 and 8 (6- and 8-mers) for computational prediction of motifs in the dataset using Seeder^70^. A sixth-order Markov Model was created for MEME^71^ using the 20,346 3′ UTR sequences of *Arabidopsis thaliana*. The motif discovery analysis was performed using MEME with a system of 8GB RAM and 4 core processor. The Seeder software was used for detecting significant motifs of length 6 (6-mers) with the same system. Background computation for analysis of 8-mers was performed on a Rocks Linux desktop cluster system with four compute nodes each with RAM memory of 16, 8, 8 and 8 GB, respectively. Random subgroups of the 3′ UTR sequences were created from each of the six gene lists. A Python script (available through GitHub: https://github.com/prabhakaranm/UTR_Regulatory_elements) was written to produce 500 subgroups for each dataset. Each subgroup contained ten 3′ UTR sequences of the genes that are randomly chosen from each of the six gene lists.

The 20,346 3′ UTR sequences of *Arabidopsis thaliana* were used to create frequency files of 6- and 8-mers for Weeder^72^, which was used to predict *de novo* motifs in the entire set of each of the six lists of 3′ UTRs (i.e. no subgroups were created).

Likewise, 19,128 5′ UTR sequences of *Arabidopsis thaliana* were downloaded from Phytozome. These were used to create the Seeder and MEME background distribution as well as the Weeder frequency files for the 6- and 8-mer analysis.

### Comparison of position weight matrices and creation of sequence logos

To eliminate redundant motifs identified in the motif discovery analysis a pairwise comparison tool, Tomtom v4.11.2^23^, available under the MEME suite, was used. The motifs obtained were matched against each other to find their similarity based on a threshold E-value cut-off of 0.05 and minimum overlap of seven nucleotides. The motifs that were found to be similar were clustered using a python script from the GimmeMotifs software^73^ to produce an average position weight matrix of all the similar motifs. Again, using the Tomtom tool, the clustered average matrix was queried against the motifs with known function from literature as well as against the RNA binding motifs discovered by Ray *et al*.^30^ (RNA/Ray2013_rbp_All_Species.meme available under MEME suite, RNA motif databases) to find matches and annotate the motifs. Finally, the WebLogo software^74^ was used to create sequence logos, which display information content in bits at each nucleotide position of 6- or 8-mers.

## Additional Information

**Accession Codes:** NCBI Gene Expression Omnibus (GEO) accession: series GSE75640.

**How to cite this article:** Munusamy, P. *et al. De novo* computational identification of stress-related sequence motifs and microRNA target sites in untranslated regions of a plant translatome. *Sci. Rep.* **7**, 43861; doi: 10.1038/srep43861 (2017).

**Publisher's note:** Springer Nature remains neutral with regard to jurisdictional claims in published maps and institutional affiliations.

## Supplementary Material

Supplementary Tables

## Figures and Tables

**Table 1:** Number of translationally regulated genes in Arabidopsis plants treated with (Defense) and without (Control) dexamethasone.

Group	NYup	NYdown	YNup	YNdown	YYup	YYdown
Genes with differential translational efficiency (total 514)	90	265	65	58	12	24
Sub-set genes with 3′ UTR sequence (total 470)	85	241	59	54	10	21
Sub-set genes with 5′ UTR sequence (total 455)	79	237	58	53	6	22

Up, up-regulated genes; down, down-regulated genes.

NY represents genes translationally regulated in control but normally regulated in treated plants.

YN represents genes translationally regulated in treated plants but normal in control plants.

YY represents genes translationally regulated in both control and treated plants.

**Table 2:** Number of uORFs found in the 5′ UTRs of translationally regulated genes during defense response.

Dataset	Number of 5′ UTR sequences	Number of 5′ UTRs containing uORFs	Number of predicted uORFs
YNdown	53	15	18
YNup	59	8	9
NYdown	241	50	62
NYup	80	13	14
YYdown	23	2	2
YYup	7	1	1

**Table 3:** Examples of translationally regulated genes containing one or more uORFs in their 5′ UTR.

Gene	Gene description	Number of uORFs	uORF location in the 5′ UTR
AT2G46830	Circadian clock associated 1, a transcription factor	1	[147–232]
AT1G54260	Winged-helix DNA-binding transcription factor family protein	2	[23–97], [156–245]
AT4G02280	Sucrose synthase enzyme 3	2	[3–107], [145–222]
AT3G29575	ABI five binding protein 3	2	[86–169], [178–267]
AT1G51620	protein kinase family protein	2	[35–169], [237–365]
AT1G74088	Unknown protein	3	[45–116], [132–197], [213–326]

**Table 4:**
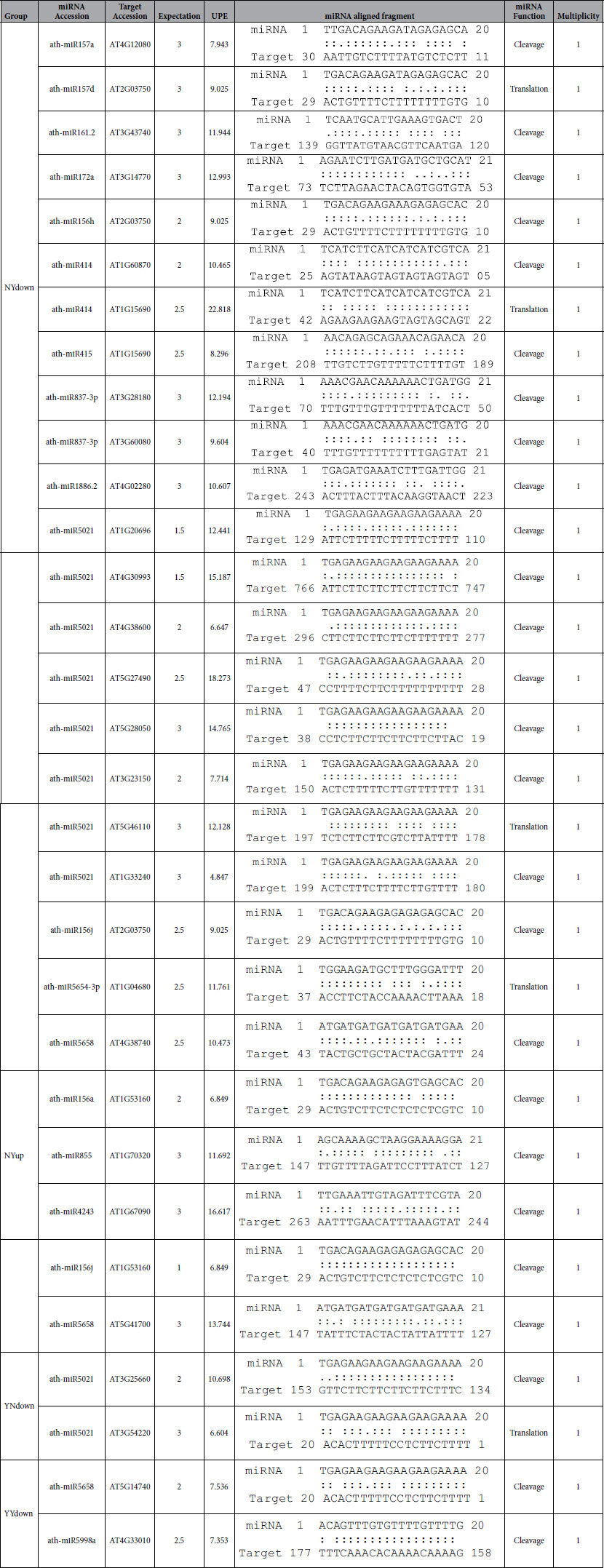
Translationally regulated genes containing miRNA target sites in their 3′ UTR predicted using psRNATarget server.

**Table 5:** Number of significant motifs identified in the 3′ UTRs of translationally regulated genes.

Motif length	*De novo* motif discovery tool	NYdown	NYup	YNdown	YNup	YYdown	YYup
6-mer	Seeder	3	16	3	1	3	None
	MEME	1	1	None	None	None	None
	Weeder	None	None	None	1	1	1
8-mer	Seeder	56	62	29	7	22	None
	MEME	6	3	2	None	None	None
	Weeder	1	1	1	1	1	1

**Table 6:** Number of significant motifs identified in the 5′ UTRs of translationally regulated genes.

Motif length	*De novo* motif discovery tool	NYdown	NYup	YNdown	YNup	YYdown	YYup
6-mer	Seeder	36	91	79	36	86	None
	MEME	87	28	81	140	27	None
	Weeder	None	None	None	1	1	1
8-mer	Seeder	57	39	50	42	49	None
	MEME	6	8	8	7	4	None
	Weeder	1	1	1	1	1	1

**Table 7:** Translationally regulated genes with motifs [U(G/A)U(A/G)U(G/A)U] recognized by Bruno-like protein in their 3′ UTR.

Gene ID	Gene description	Position	Sequence that matches to Bruno-like protein motif
AT2G06520	Encodes a protein similar to spinach photosystem II subunit PsbX	−94	C UGUGAU
AT4G38460	Geranylgeranyl reductase involved in isoprenoid biosynthetic process	−169	C UGUGAU
AT5G57350	Arabidopsis H(+)-ATPase	−230	C UGUGAU
AT2G06520	Encodes a protein similar to spinach photosystem II subunit PsbX	−93	UGUGUAU U
AT5G53030	Unknown protein	−70	UGUGUAU A
AT2G29670	Tetracopeptide repeat-like superfamily protein	−256	U UAUGUAU
AT4G38460	Geranylgeranyl reductase involved in isoprenoid biosynthetic process	−19	C UAUGUAU
AT4G38460	Geranylgeranyl reductase involved in isoprenoid biosynthetic process	−15	G UAUGUAU
AT4G25570	Alpha/beta-Hydrolases superfamily protein	−143	U UAUGUAU
AT4G14500	Polyketide cyclase/dehydrase and lipid transport superfamily protein	−54	A UAUGUAU
AT1G56280	Unknown protein	−289	C UAUGUAU
AT3G54220	Similar to DNA binding protein containing basic-leucine zipper region	−95	U UAUGUAU
AT4G30400	RING/U-box superfamily protein	111	UAUCAUA U
AT3G46450	Cytosolic factor family protein/Phosphoglyceride transfer family protein	59, 95	AUACAUA G
AT3G54220	Similar to DNA binding protein containing basic-leucine zipper region	138	AUACAUA G
AT2G34070	Member of TRICHOME BIREFRINGENCE-LIKE gene family	11	A AUACACA
AT2G29670	Tetracopeptide repeat-like superfamily protein	66	U AUACACA

*Bold and underlined represents the sequence that matches to motif.

**Table 8:** Translationally regulated genes with motifs [UGUA (A/C/U) AUA] recognized by Pumilio protein in their 3′ UTR.

Gene ID	Gene description	Position	Sequence that matches to Pumilio protein motif
AT4G25570	Encodes cytochrome b561	−85	UG UGUAAA
AT3G01400	ARM repeat superfamily protein	−165	GG UGUAUA
AT5G53030	Unknown protein	−70	UG UGUAUAU
AT1G78630	Embryo defective 1473 involved in embryo development	206	UG ACAUGU
AT3G49670	Encodes a CLAVATA1-related kinase-like protein	204	A UAUGUAC
AT4G30400	RING/U-box superfamily protein	111	AU ACAUAU
AT4G00490	Encodes a chloroplast beta-amylase enzyme	−220	UU ACAUAU
AT4G17080	Histone H3 K4-specific methyltransferase SET7/9 family protein	−132	GU ACAUAU

*Bold and underlined represents the sequence that matches to motif.
