# Supplementary material for: De novo computational identification of stress-related sequence motifs and microRNA target sites in untranslated regions of a plant translatome
Source: Sci Rep. 2017 Mar 9;7:43861. doi: 10.1038/srep43861 (PMC5343461; doi:10.1038/srep43861)
Supplement: Supplementary Tables [file srep43861-s1.pdf]

De novo computational identification of stress-related sequence motifs and microRNA target sites in untranslated regions of a plant translatome

Prabhakaran Munusamy, Yevgen Zolotarov, Louis-Valentin Meteigner, Peter Moffett, Martina Strömvik

Supplementary Table 1. List of translationally regulated genes belonging to different groups based on differential expression analysis.

Group	Gene IDs
**NYup**	AT2G05710, AT5G59880, AT3G29575, AT5G60440, AT3G48000, AT1G79440, AT3G62290, AT1G04800, AT1G06530, AT1G10940, AT1G20920, AT1G20970, AT1G23130, AT1G25275, AT1G31540, AT1G36730, AT1G54410, AT1G64370, AT1G74250, AT2G04520, AT2G07706, AT2G17240, AT2G22370, AT2G23120, AT2G26440, AT2G31410, AT2G39400, AT2G39570, AT3G04300, AT3G05220, AT3G08530, AT3G09160, AT3G17800, AT3G28940, AT3G57930, AT3G60540, AT4G08850, AT4G27290, AT4G27310, AT5G10625, AT5G10840, AT5G15940, AT5G17190, AT5G24230, AT5G38070, AT5G42220, AT5G61390, AT5G63370, AT5G63390, AT5G67410, AT3G51800,AT3G19190, AT5G28540, AT3G56240, AT4G11920, AT5G58940, AT1G08450, AT4G26430, AT5G49740, AT1G17220, AT4G36810, AT1G05200, AT5G53460, AT5G35630, AT2G33210, AT3G02875, AT2G02130, AT4G37300, AT2G35630, AT4G37260, AT1G54330, AT5G47640, AT4G24020, AT3G52525, AT5G54160, AT5G43980, AT1G55670, AT1G67090, AT1G21450, AT4G29160, AT5G67250, AT1G53160, AT1G80070,AT5G49190, AT2G18260, AT3G16640, AT2G43520, AT5G41700, AT1G70320, AT5G64810
**NYdown**	AT1G36160, AT4G12080, AT5G19140, AT4G35450, AT5G46110, AT5G19220, AT1G01320, AT1G02305, AT1G03140, AT1G04680, AT1G07080, AT1G09310, AT1G11240, AT1G11360, AT1G15230, AT1G16000, AT1G18620, AT1G23390, AT1G25570, AT1G26761, AT1G26800, AT1G26900, AT1G34110, AT1G47970, AT1G48300, AT1G50950, AT1G56423, AT1G61740, AT1G64510, AT1G66100, AT1G66840, AT1G67250, AT1G68730, AT1G69980, AT1G75280, AT1G77020, AT1G77170, AT1G78230, AT1G80130, AT1G80180, AT1G80780, AT1G80940, AT2G03505, AT2G03750, AT2G04340, AT2G15020, AT2G16595, AT2G17350, AT2G23200, AT2G24090, AT2G26390, AT2G28600, AT2G35640, AT2G35680, AT2G37750, AT2G41870, AT2G42700, AT2G45750, AT2G46780, AT3G02400, AT3G03150, AT3G05900, AT3G07230, AT3G09980, AT3G10020, AT3G11930, AT3G13590, AT3G14067, AT3G14190, AT3G14420, AT3G14770, AT3G16350, AT3G16370, AT3G16650, AT3G19440, AT3G22440, AT3G23450, AT3G24490, AT3G26450, AT3G27100, AT3G27460, AT3G29240, AT3G30390, AT3G43740, AT3G47070, AT3G48210, AT3G52500, AT3G56510, AT3G57400, AT3G57490, AT3G57910, AT3G58840, AT3G60080, AT3G61260, AT4G00026, AT4G00895, AT4G09950, AT4G10300, AT4G11320, AT4G13840, AT4G15030, AT4G19420,

	AT4G21450, AT4G21920, AT4G23670, AT4G26630, AT4G27450, AT4G28080, AT4G28240, AT4G30993, AT4G36420, AT4G37470, AT4G40030, AT5G01200, AT5G01520, AT5G02160, AT5G02410, AT5G03660, AT5G06530, AT5G09450, AT5G10350, AT5G14510, AT5G15750, AT5G19590, AT5G20700, AT5G20820, AT5G21430, AT5G22580, AT5G23760, AT5G25010, AT5G25280, AT5G25630, AT5G26610, AT5G27490, AT5G28050, AT5G38420, AT5G38980, AT5G40500, AT5G42680, AT5G43180, AT5G43830, AT5G46870, AT5G47550, AT5G49700, AT5G50390, AT5G51570, AT5G52740, AT5G52990, AT5G53880, AT5G58260, AT5G65207, AT5G65220, AT5G65380, AT5G66490, AT4G16520, AT4G10120, AT1G15690, AT4G36540, AT3G01500, AT1G42990, AT3G01500, AT2G21890, AT4G37970, AT3G56240, AT4G34050, AT1G02800, AT4G25080, AT1G48260, AT4G15560, AT5G43860, AT5G50920, AT2G47400, AT2G46410, AT4G04720, AT5G40380, AT3G56940, AT1G08830, AT3G28180, AT4G17090, AT3G54900, AT5G23530, AT5G23060, AT3G26125, AT5G42800, AT1G08930, AT1G20900, AT3G23150, AT4G33630, AT4G30950, AT5G53170, AT1G17220, AT5G53170, AT1G42970, AT2G18380, AT4G36240, AT1G03970, AT5G48030, AT4G02520, AT2G47730, AT1G33240, AT2G22430, AT1G20696, AT5G23420, AT1G04100, AT4G02670, AT1G64790, AT4G38600, AT1G01790, AT3G13850, AT5G01530, AT1G61520, AT5G01530, AT5G44610, AT4G26760, AT2G33820, AT1G11760, AT4G00950, AT1G60870, AT1G33520, AT4G04840, AT2G33480, AT4G37260, AT1G21640, AT5G47640, AT2G19620, AT3G25882, AT5G55810, AT1G79280, AT4G20260, AT2G26020, AT1G04980, AT5G64070, AT4G11840, AT5G38150, AT2G42120, AT5G45070, AT5G61770, AT2G42600, AT1G31330, AT1G52230, AT5G66570, AT4G20360, AT1G13260, AT1G01360, AT2G15080, AT5G25610, AT4G38740, AT1G52240, AT1G60620, AT5G49010, AT2G46340, AT5G24150, AT2G32765, AT4G02280, AT3G55980, AT5G42980, AT2G47770, AT5G46740, AT2G06530, AT5G64530, AT5G64570, AT5G41000, AT1G58340, AT1G56590, AT1G10970
**YNup**	AT3G28970, AT1G09580, AT1G51620, AT1G52800, AT1G55152, AT1G61170, AT1G66530, AT1G67170, AT1G67800, AT1G72810, AT1G76240, AT2G15300, AT2G17970, AT2G24650, AT2G30105, AT2G36170, AT2G44370, AT2G44580, AT3G04970, AT3G09035, AT3G09070, AT3G11720, AT3G23540, AT3G44220, AT3G57000, AT4G04950, AT4G09490, AT4G16695, AT4G17430, AT4G18400, AT4G25320, AT5G01380, AT5G01790, AT5G08430, AT5G16720, AT5G17610, AT5G25210, AT5G45590, AT5G46160, AT5G46680, AT5G56940, AT5G67090,

	AT1G80420, AT2G26000, AT4G15130, AT1G25425, AT5G39210, AT2G45080, AT4G18750, AT2G36640, AT4G05110, AT2G37640, AT1G10240, AT1G79460, AT4G02290, AT3G51910, AT1G48050, AT4G26890, AT5G44160, AT5G15860, AT5G02010, AT4G34410, AT3G28150, AT2G26580, AT3G21175
**YNdown**	AT4G25570, AT1G62700, AT1G01630, AT1G07280, AT1G25440, AT1G54260, AT1G56570, AT1G60000, AT1G68400, AT1G68520, AT1G70180, AT1G74088, AT2G29670, AT2G32560, AT2G44230, AT3G01400, AT3G01660, AT3G23900, AT3G25660, AT3G46450, AT3G62050, AT4G04880, AT4G14500, AT4G17080, AT4G17616, AT4G30400, AT5G45670, AT5G53030, AT5G56850, AT2G25900, AT3G49670,AT4G00490, AT2G43360, AT5G18930, AT2G46830, AT1G29395, AT4G11460, AT3G26300, AT1G56280, AT1G72630, AT2G24762, AT4G38460, AT5G57350, AT1G17560, AT1G15820, AT1G22640, AT2G32100, AT1G04520, AT4G03280, AT1G32440, AT2G06520, AT4G02080, AT3G54220, AT1G75540, AT4G13460, AT2G34070, AT2G46030, AT1G78630
**YYup**	AT1G48090, AT2G07687, AT2G07698, AT2G07707, AT2G07727, AT2G07732, AT2G07738, AT4G21020, AT3G02260, AT1G75830, AT5G26000, AT5G25980
**YYdown**	AT4G29140, AT1G06080, AT1G74840, AT2G18300, AT2G31141, AT3G54500, AT5G07140, AT5G14740, AT5G10930, AT1G09340, AT2G21330, AT4G38970, AT1G14070, AT3G26650, AT4G33010, AT2G26080, AT5G04140, AT1G07900, AT2G34430, AT2G34420, AT3G47470, AT5G54270, AT4G10340, AT2G39800

Up, up-regulated genes; down, down-regulated genes

NY represents genes translationally regulated in control but normally regulated in treated plants

YN represents genes translationally regulated in treated plants but normal in control plants

YY represents genes translationally regulated in both control and treated plants

Supplementary Table 2. Translationally regulated genes containing uORFs in their 5′ UTR

Gene ID	5′ UTR Length	uORF Position	uORF Sequence
AT3G19440.1	83	uORF [2,70]	ATGACTATCGGTTCCATCTTTCCCAAGTTCCTCTGCT CCAAAACCTCTTAAACCCTAATTCGAATT TAG
AT4G10300.1	102	uORF [3,74]	ATGCTATACGATTATATTGGACAGAAAATGAAAAAT TTAAAATGGAGTTTTCTATTGCATTTTGTAAGC TGA
**AT4G36540.1**	116	uORF [2,82]	ATGGATTACTTTTCTTCCACACATATATAACTTAACT CTCTCTTTTTCTCTTTTGCTTTAACCCCCTCAAAGAAA AGA TAA
AT1G60620.1	119	uORF [6,74]	ATGAAATTGAAGAACGAAATTGAAGTGAAAAGTGA AATCGTGCAATTTTCATCTTCCTCTTCTCCT TAA
AT1G21640.1	129	uORF [13,105]	ATGAAGAAGAGGACGAATCCACGAGGTCCACCAAAT TCCTGCAAACTTCCAAAACACCATCTCAAATATCCAC ATTCTCCGCCATTACCC TGA
AT5G19140.1	129	uORF [17,88]	ATGTCCGATATATCGTTACCAGAGAAAGAGAGCTCA GAGATTTAGTTAGCTTAATTTTTCTTTTCGTTT TGA
AT3G11930.1	134	uORF [1,111]	ATGCATATCTTATTGCACGTGGCCGGTGCCCTTTGAG ACGTATCACCCTCTACATGCAATATAAAAAGCATTG CAAATGAACCAAAGTTTGATCACATCCATCACTTA TAA
AT3G03150.1	147	uORF [12,86]	ATGCATATATATAAGCACACATTAAACGGAGTTAAA CTCATCATACATTACTGACTAAAAGAAGCAAAATCT TGA
AT4G23670.1	148	uORF [11,100]	ATGATTTAAAGAAACGGTAGAAACCTTGTTTTTAACC GTCACGGTCCCTATATAAACCTATGAGCTAGCGACG ACAAGTATTCAGTA TAA
AT4G13840.1	160	uORF [41,127]	ATGTCCAAGCAAAGGTTCATATAATTTTATGTTATTA AATTTGCTTTTTTTTTGTTAAAGTGTTACATACATCGA TCGACTTGT TAG

**AT1G56423.1**	163	uORF [17,136]	ATGGCTTTTGGAGTCTTTGAATATTCCTTCCCTCTGA CTTATGGTTTCCAGAAACCTTCCTCACACTCAAATGT TTTGTTCTTCTTAATCACAATTTCAGAATTGGTCTTTT ACGGT TAA
AT2G42700.1	163	uORF [1,72]	ATGTTCGTCGTCGGTGAGATTTCCCGGCGAAGCGAA AGCAGTGTTTCTCCGGTGGTTCAGGCATCAGTC TGA
AT5G42980.1	165	uORF [4,90]	ATGTTTTATTAATTATTATTATTATTCCATTTGCATTA AAATATAATAAAAAGATGTAAGATTCTCTCCGCCTC CTTCTCTCTA TAA
AT1G01360.1	167	uORF [4,126]	ATGTCGGTGGGTTCATTAAAAGAAACCAAAAAACAT AAACAAGTAATTTTGTTTTGGCATAACGAAGCATCTT CTTCTTCTTCCTTGTATTTATTATCCATTTCCAGAGAT TCTCCCTTG TGA
AT1G80130.1	168	uORF [41,106]	ATGCGGCTTACCATTATATAAGACTCTGGTAGACTAC TCTCATTATATACATTATAAAGATAC TGA
AT1G42970.1	171	uORF [8,73]	ATGTATCTTACCTACATAATAGCCACATATTACTTTG CTTCACTTTGAACCATTGGTTGGTTT TGA
**AT4G26760.1**	182	uORF [4,159]	ATGGGTCCCACTTTTCCCATTTCCCAACCTCCATAAC CAAAGTTGGAGATTTCTCTGTTTCCTATGCTCTCTCTC TCTCTTACTCTCACAGTGACTATTCGTCGCTTCAGAT CTAAAAGAGAGGAAGATAAACCATTTGGATTCAATA TCGAT TAA
**AT1G11360.1**	189	uORF [15,98]	ATGGTTTTGGTTATATATTTTTACAGCCGTTAGATCTT CAAACCGATCAATCTAACGGTTCTTAACAATATCCA ATTCTCA TAA
AT1G10970.1	206	uORF [13,87]	ATGCGTCAGTCTCTCATCAGTGTTCAACTGCCACGGA GCGAACCGATTCCTAATTGCAACGTCCCGAGTCCA TAG
AT1G10970.1	206	uORF [89,172]	ATGTCGACACTCTTTCACTCTTTCTCCAAGTTGCCTCC TTTGAGTCCTTTCTCATATTTTATAGACTCACTTTCTG TTTCT TGA
**AT2G22430.1**	218	uORF [7,150]	ATGGAAAACGCTCATCAACCTCAACAATCTCTCTCCT CTCTCTCTCTGTATATAGAAGAATCTCCATTGTCTTT AATTTCTCTCCATTTCTCTTTCTCTCTTCCTTCAAAGT TTTCTCTTTTCTTGATGGGTTTAAGAGAG TAA

**AT3G16370.1**	218	uORF [42,116]	ATGGTTGGAGAAATATTGAATAAGAGAGAGAGAGAGAGAGAGAGAGATAGAGTTTCAGAGTTGACACAACAT TGA
AT1G52240.1	222	uORF [35,136]	ATGCTTATACGTACATTTCTTCTTGAAAAGACTTTGAATCCAAGAACCCGTGAGGAAGTAATCCCAGTGAAGAGAAGACGACAAAGAAAAAGGAGCCTA TAA
**AT1G33240.1**	226	uORF [133,210]	ATGGTTTTATGAGATTTATATCAAAAAACATTGAGGAGCTAGAGAGAAAGAGAGAGTGTGTGTGTAGAAAAAGAT TGA
AT2G17350.1	226	uORF [32,118]	ATGTCTCTGTCCCTTTCTTCCTCAGTTCTCACGCAAGCTTCAACGAGAAAAGATCGGAATCTCGCCGTCGGTAAAGCGCCGCCG TAA
AT3G47070.1	226	uORF [23,193]	ATGTATCCAAGTGATTCAAAGAAAAATGTGTGAAGGGAAAAAAGAAAGAGGATAAGACACTTGTGGAGAGCTTGTGCCAAAAAAATCCAACGGCTCAGATTCAACCAACCTCGTTATAATCCCTTCGTAACAAATGCCACACGAAGACACACATACACACACTACCGA TAA
AT5G49010.1	233	uORF [144,221]	ATGTTTTTATTTCCCGCTCGAGCAAATTGATCTGATCCGAGTCTTTCAAGCGGGGAGAGAAAGAGAGAGATTCGT TAG
AT2G16595.1	236	uORF [6,137]	ATGTTATAACACCTATGGGCAAGGTGTTTCTAAATTGGTAAGTCGGAAAACGACGTCGTTTACAAAATCCGATCTGCACACACACTCACATTCATATGTCGTTCTTCGTCAACGTCGGTGAAGAAAGCA TAA
AT4G02280.1	239	uORF [3,107]	ATGTACTCTCCTCTAACATAAACACGTCACTTGTAGCGAAAACAGTATCAAGAAAAAGAGAAGATCAAACACGTCTTCTTTTCTCTCTCTCTCTTTGTCGCC TAA
AT4G02280.1	239	uORF [145,222]	ATGATTTTTCCTTTTAGTAGCAATCGTTGGTGATTCGAAAAACCAAACTTTTCTCGGACTAGGATTCTAGGGTTT TAG
AT1G18620.1	243	uORF [24,227]	ATGTGGCTGTCTTTTTGTTTTGTTCTCTGAGCAGAACCAGGAGGAAACATTTTGCAGAGGATTTACTGGAAGCACAATAAAGAAGAGTTAAGACACTGTCACTACAGAGAGAAGAGTTCAAATTCTCTCCTTTTTGTTACCAGTAGCAAGTTCCGGCGCCGGAGATTCGCCGGAGAACCCTCCTGGTTTCAGGTGAACTTC TAG
AT1G80780.1	250	uORF [119,217]	ATGAAACCAGATCGGGTCTTCCTCTAGATCCGTTCTAGGTTTTCTATTTCCACCGTCTTATCATTATCATCATCATCAATTCATCATCATCATCAA TAA

AT3G24490.1	252	uORF [7,93]	ATGTTCACCGGTGGTGGTAGCTTTTCTTCTACTCCGA<br>CCCGGCTGAAAATGCAAATCCCTAACCCTAGACACG<br>GCCATGGTTTA TAA
AT5G52990.1	260	uORF [18,83]	ATGATCTCCATTTCTGTTATTTTCCCTAGAAATTTTCG<br>CAAACCCTAAAAAGAGAGAGAAACA TAA
AT5G52990.1	260	uORF [128,217]	ATGTATTTCTAAAAAGAGAGAGAAATCCTCTGATCT<br>CCTCTCTGATTCGATCGAGATCGCATCATTTCCCTAA<br>TGATTTCAAAAACC TAA
AT1G34110.1	277	uORF [19,96]	ATGTAAAGACCTCACTCTTAGCTTCACATGGTTATCT<br>ACAGTTATCGCTCTTGATGTTCTTGTTCTGCAATGGT<br>T TAG
AT3G28180.1	281	uORF [20,106]	ATGATCCTCGTCTCTCTTTCTTTCTCTCTCTCAATCAC<br>AAGTTTCAGTTACACAGCTGAACCCAAAGTATCTCA<br>CATTCTGATC TAA
AT1G17220.1	300	uORF [148,243]	ATGCTTTCGGATTTGATTCGTCGATAACCCCAATTTC<br>TTTACAAAGTTGAAGTTTTTATGTCATTTCTGGTTCG<br>AAATTCGTTGCAATTGGAA TGA
AT4G19420.1	300	uORF [197,283]	ATGTGCTGAAACTGAGTCATCGTCCGAGTTAGCAAA<br>GTGAAACTTCGAAGTTTCTCTTACACTAATCTCAGGT<br>TTGGGATTTTG TAA
AT2G46340.1	310	uORF [88,219]	ATGTAATTATCAATAGTATATGACAAATTTATAGCCT<br>CACCTTTCTTCTTCTTCTTCTTCTTCTTCTGTGTGTTTT<br>TGAATTTGCTTTCTTCAAGAGGTTTCTTCTCCACACC<br>GCTTCGGTGTATGATC TGA
AT4G15560.1	311	uORF [37,144]	ATGAAGTTGGCTTTCTTGTCGTTTTACTTCATCACCCC<br>ATTTTTTTAAAGTCTCCATCTTTATACTTCTTCAACTC<br>TCCACCACCACCATTGTCACCACCACATT TAA
**AT3G60080.1**	327	uORF [127,192]	ATGGTAGGCCCTTTATAAACCAACTTTAATCCATTTT<br>AACTTTTATATATTAAAAAAGGGCCT TGA
AT3G01500.1	330	uORF [102,326]	ATGTCGACCGCTCCTCTCTCCGGCTTCTTTCTCACTTC<br>ACTTTCTCCTTCTCAATCTTCTCTCCAGAAACTCTCTC<br>TTCGTACTTCTTCCACCGTCGCTTGCCTCCCACCCGC<br>CTCTTCTTCTTCCTCATCTTCCTCCTCCTCGTCTTCCC<br>GTTCCGTTCCAACGCTTATCCGTAACGAGCCAGTTTT<br>TGCCGCTCCTGCTCCTATCATTGCCCCTTATTGG TGA

AT3G52500.1	333	uORF [42,140]	ATGTATTGGCTGGCTCTGTATCATCATGTCTACGTAGACAGAGATGTATCATGTCGACTAAACAAAAATTACATTTCCACGTATAAGATTTACTTA TAG
AT5G40380.1	349	uORF [16,210]	ATGAATAGTAGTTTTGTAATTAATTAATCAAGAATTAAAAAAAGTTCTATCAAAATCTAAATATATAGTCTTTTGTTTGTAATATGTTCTGCTTTTTACAAATATGGACTTTTGGTATCAATCATATTCACTTGTTCAGTATTATTCGAATTTCTTGAATCATATAAAAACGAATTATGTAATAAAACGATA TAA
AT5G40380.1	349	uORF [232,300]	ATGTCAATTGTCATATAACTATATAGTATATATATATAAACCTTTCTGCAAATACAATCCTATATT TAA
**AT5G01520.1**	365	uORF [196,267]	ATGGAAATGGGAGAGAGAATCAATTCGAGTTGTTGTTAGGGGGTTTGCGATATTTTAGGCTTCTGTCGG TGA
AT5G46110.1	371	uORF [49,123]	ATGATGATGGTGATATGGAACTTCGATTGGCTAATATTCACTGTGTCTCTAAAAACCATCCACTTATCAAGA TAA
**AT5G46110.1**	371	uORF [125,202]	ATGGACCCTACACTCATCCAATCTAAACCAGTATCTCAAGATTCTTATCTAATTACATCATTCTCTACCGTTAGA TGA
**AT3G23150.1**	396	uORF [39,107]	ATGGCGGTTTTCCGGCACTAATCATCTCCGGCATATATAAATAAACGTACTTCACGTTTTTTTATA TAA
AT3G23150.1	396	uORF [239,316]	ATGAGAGGAAGATCGGAATGTCGAAGAGAATTAGAAGATTCTCGTACATCACTTCGTTGGAATTTCACAGGTCGA TGA
AT2G41870.1	417	uORF [48,206]	ATGTGAAAAATACAATCGATCGCATTATCTTTATCCCTAGCTAATCATTCATGTACAAGCATGTCTCCGAAGGTTAAAAGCAGTCGCTATTTACCGGACCAACGTAGTTTTCTCGAAGTGGTGGTCCGTTGTCATATTTTAAATTTATCACCTTCT TGA
AT2G41870.1	417	uORF [234,344]	ATGTAGTGTATATTTTTTCCTCTAACCTAATTAAAATCAAAACAAAATCCTTTGACCCAATTAGCTTCGCGATATATCAGAAGAGATCAAACTACTTTGATCAGACCA TGA
AT1G20900.1	468	uORF [29,133]	ATGAATCTCAAGCTTCTCTCTCCTTTTTTTCCCATAGCACATCAGAATCGCTAAATACGACTCCTATGCAAAGAAGAAGCTACTTCTTTCTCTTGCCCTAAT TAA

AT1G20900.1	468	uORF [165,314]	ATGAGAGAGATCATTTAACATAAGTCACCTTTTTTAT ATCTTTTGCTTCGTCTTTAATTTAGTTCTGTTCTTGGT CTGTTTCTATATTTTGTCGGCTTGCGTAACCGATCAC ACCTTAATGCTTTAGCTATTGTTTCCTCAAAATCA TGA
**AT2G35680.1**	495	uORF [251,370]	ATGGTGGTGATCTCCCTGAAGATTTTGAATTCAAAAT TTCTTCGGTTGCTTCCTCTTGACTTTATCGCGATTTCG ATTACGTGTAGACTTTCTTCTTGGCTTTGTCTTCGAAT CAAT TAG
AT3G14770.1	502	uORF [1,156]	ATGATTTTCAAAACCTTTCGAGTTTTTTCAATCTTTTT AATCAATGGGATCAAATTTCGAATATCATTAAGACTT TTAAGAGAATTTTTAGACCATTATGCATATGTTTACC TTAATTTTTTCATGATCTATATTATTTTAAATGGAATT TAT TAA
AT3G14770.1	502	uORF [162,305]	ATGTATAACGTTACGACACTTTTTCGTAATTAATTTG TTTGTAAGATAGTTAAACAGTAGACAAAGCCAAATA TGGACCGCATATATATAGTCGTACACATTCAGCCTTT TGCTCCTTACCTTTTTAAGAAAGTCTTCTTT TAA
AT2G47730.1	745	uORF [6,89]	ATGAATTGGTTTGATAACTTTCGATTTTACTTGGAAA TTAACTATTACAAGAAAAATCTAATTTCAAATAGTG GAATGATT TGA
AT2G47730.1	745	uORF [111,326]	ATGATAACAATTACAAAATAAAAATGAGACGGCCGG CTGTAGAATAATATGACGACGTGAAGCAAAAGCAGG AGTGCGACAGAAACCTCTCTAACCAAATTGCTTTTAG TTATATTCTTCTTCGTCTTGACGCTACTCTTTACCTTT TTTTATCTGATTTCAGATTTCTCCAGAATCTTCAGATT CCCATGGCCACTGTTCATGTTGTCGGTC TGA
AT2G47730.1	745	uORF [375,500]	ATGCAATTCATGCTGCTTTATCACCGTCATCATCTCT ATCATTACTCAGAGGAGTGTTACCAAAGTATTTCAGC TCAGAGTTGTCAGTGGCTCAGTTCCGGTTGGTCGAAG GATCTTAATACA TAG
AT2G47730.1	745	uORF [513,611]	ATGAGAAGAACACAGATTCTTGGGATGGATGGGAGC TTCTACAAAGGCCAGTTTTGATCCAGTACACGGAGG AGAGATGTCTCAGCTCGAGTATCA TAA
**AT4G36810.1**	139	uORF [23,94]	ATGGCCAATCCAATTCTCCATTCATAATTTTGACTCC ACTTTCCAAAAAAAACAAAAAAAAATAAATAC TAA
**AT5G10625.1**	147	uORF [13,126]	ATGGCTATAAATACAGAGCCCGCACACAAACTCTCT CATAATTCATAAGTAAACACCAAAGCAATCGCTCGA GACCCCTTACAAATATCGATCTCTCTCTTTCTATATA CA TAA

AT5G59880.1	151	uORF [1,132]	ATGTTTACTTATTCGGACTAGAGAGCTTCCGCATAAAGCTGAGGAAAAAAAAGAGAGAAGACGCACACCGAAGAAAACACACAAGACTCCATTATTCTCCTGCTTCTTCGTCTCTTCAATTATTTTCTC TGA
AT4G37300.1	164	uORF [19,108]	ATGAAATTTGTAATGAGAAAATTAAAGGACTAAAAAGGCCCACTAAAAGCCCATATACATTTTCTTTTAAACACAACTAAAAACCAG TGA
AT3G48000.1	177	uORF [12,125]	ATGTGTTAGAGATATCCGGAGATCTCAACCGTTGGATTCTTTCTCCTTCAAATCAAATTATAAATCCCATCAAACAGAGAGAGAGAGAGAAGAGGAGGAGAATTCGAAGAA TAA
AT2G43520.1	186	uORF [34,102]	ATGATAATTTCTAGACCCAACGATAAAGATAAAATAGTAGCATCGCACCACAACAAGTGTTGAGCA TGA
AT1G05200.1	227	uORF [16,132]	ATGTAAAGCACCAGTTTCTAGAATCAAAGGGCCTCATATAAATACATTATTTTATACACTATAAATAAATATACAGACACAAAAATCTGTATACACCAATCTCTCTACAGAATC TAA
AT4G26430.1	237	uORF [54,155]	ATGCTATAATCTTTTCCATTATTTTTCTAACAAAAGAAAGTTATATATGGGCTAAACTTAGAAGGAAAGCCCAAGAGGCCAAGACTATATAAAGATTCA TGA
**AT3G17800.1**	278	uORF [35,100]	ATGGTCTCAAAATATTTAAACGAAAAATATAATTTTCTGAGTCCTTTCATTTATTATTATAAA TAA
AT1G17220.1	300	uORF [148,243]	ATGCTTTCGGATTTGATTCGTCGATAACCCCAATTTCTTTACAAAGTTGAAGTTTTTATGTCATTTCTGGTTCGAAATTCGTTGCAATTGGAA TGA
AT5G35630.1	324	uORF [15,194]	ATGAGTATTGAAGTTGAGATAGAGGAGGTACAAGGAGACCTTATCTGCAGAAGACAAAAAGCCATTTTTAGCAAAACTAAAGAAAGAAAAAAGATTGAAACACAAATATGCGCCACTCGTAGTCCACCCCTATCTCTTTGGCAAAAGCCACTTCACTCTTTTTCCCTTTTTATATAT TAA
AT3G29575.1	347	uORF [86,169]	ATGTTGAAATTTTGTGGGATTTTTTTTTATTTCTTTATTAAACTTTTTTTTATTGAATTTATAAAAAGGGAAGGTCGTCAT TAA
**AT3G29575.1**	347	uORF [178,267]	ATGGAATCTTCCAAAATTTGATATTTTGCTGTTTTCTTGGGATTTGAATTGCTCTTTATCATCAAGAATCTGTTAAAATTTCTAATC TAA

AT1G36730.1	470	uORF [264,437]	ATGTCTGAACAGGCTTCTCTCTTTCAATCATCTATCC GGCTTTTAGGAGAGTACTGTCCTATATCTAGGAAAA AGTCCACTCATAAAGTTGGTGGTTTGCGTTTTGAGGA GGACATCACGAGCTGTATTGGATTTCAGTTTATGCAA CTGAGTGATTATCACTGCCTCTTC TGA
AT1G29395.1	82	uORF [6,74]	ATGCAGTGATATAGTCGTTTAACAGAAAGATCACTC CGTCAATCTTTCCACCACTGTGACTAAATC TAA
AT4G00490.1	102	uORF [3,74]	ATGATTCTTGGATCCCATGAATAAGCCTTCACGTGGC TTTTGATTTCACATTCCACGAAAATCCAATTT TAA
AT4G03280.1	168	uORF [8,79]	ATGTGGAAGCAGAGGCTGGCGCGTGCCTTATCTGCT TCTCTCTCTCCAGTGGTCTCAAATCTCTCTGTG TGA
AT1G25440.1	176	uORF [17,109]	ATGCGCTTCATGCGGGTCATCCTCTTAATCTCAAACT CTCTAGGACTACACTAAATCTAACTTTTTGCAGAGAG CAAAAGATTCAATAAT TGA
AT1G01630.1	193	uORF [5,94]	ATGAGTAGAATTTTTGTAAGGAAGAGAGTAGTTGGT GAGCGTCGCCATTGAAGGGGAAAGCAGCCCAACTTT GCATTGAATGACACT TAA
**AT3G23900.1**	193	uORF [82,162]	ATGGTAATGGAATCTTAGGCTCTGCTTCTTGTTGATT GTAAATTCTCGAATTAGGGATTTAGCTCTCATTTGAG AGAT TGA
AT4G14500.1	215	uORF [98,205]	ATGTCGCTTAAATTTTTAGCGATCTTCGAATCCCATT TTCCTTCGGGAATCTGACATTTTTTCTCGGGAAATTT TTTTATCCAAATCGGAGTAAAGTATCGAATC TAG
AT3G46450.1	241	uORF [43,138]	ATGAACTTCGGAGAGAAGACGAAATCTCGCTCGCTT TCGCCGCCGTGAAATTTGATTCTCCGATCACTACAAC GATTCCGATCTGTCCTCCGT TGA
AT5G53030.1	245	uORF [57,188]	ATGTCTTCTCTAACCTCTCCTCCCACTATACATCGTCC TTAACTTTTCCTTTTTCTCGTTCAAATGAATCCAATTC CTATAAAGCTTTCTTTAATTTTTCTCTCCTATTTAACC CAAACCCATTTCATA TAA
**AT2G46830.1**	249	uORF [142,237]	ATGGAATCTTTATCGAATCCAAGCTGATTTTGTTTCT TTCATTGAATCATCTCTCTAAAGTGGAATTTTGTAAA GAGAAGATCTGAAGTTGTG TAG

**AT1G04520.1**	274	uORF [12,98]	ATGGTCATCAAAAGCCAAAGCCAAAGCCAAAAAAACAAAGAACCCAGAAACCTAGAAGACAGAAACAAAAATCAAAGCTTTAC TGA
**AT1G54260.1**	276	uORF [23,97]	ATGGACGGTGGAAGAAGGAAGCCCTTCTCGCCGGACTAGCCAAGCACGGTTCCGGAAAATGGACAAGTATTTTAG
**AT1G54260.1**	276	uORF [156,245]	ATGGCGTAATATAAAGAAATCAAGGACACAATCACTCGAATCTACCTCCATTTTCACTTCACCTTCAGAGACTTCTCGTTCTGTTGT TAG
AT3G54220.1	392	uORF [17,247]	ATGAATAGAGATAGAAAGAGTCATTAAATGTACGAAGCGACATTCACAATAATTCGAAAGGTGGAAGACGACTTAGATACGGCCAGGCTTCACTGTCCTCCTCGTCCTCCTCAATTACCCCTAACCCCTTTTTCCGGGATTCATCTCCAACCCACATCCTTCCAAATTCTCACCCCCTCACTGAGTTTTTGCTTTTTCTCCTCATCGGAGATCGTGAAGACGATCAAG TAA
**AT2G25900.1**	394	uORF [6,173]	ATGGATAAAGAAAAAATATTTTTATTTTGTTTTATTATTCTCTTTTAAAGTTAAAGACTTTAGGAAAAAAACAAAAGAAAAAAAGTAAATGGAGAGAAAAAAGCTTTTAAAACTTTAAAAATTAATAAAGTTTTAAAAAAAAAAAAAAAAAAAAAAAGACGAACA TGA
AT1G74088.1	404	uORF [45,116]	ATGTCACTCTCTAGTCTCTCCTCCATCTCTCGATTAATCCCAGCAAGATCCTACTTGAAGGAGACGCAA TAG
AT1G74088.1	404	uORF [132,197]	ATGACCGTACAAATTTCACTGAGAAGGCAACAAGCGCTTCCGCGTACTCCTGACAATGTTAGA TGA
AT1G74088.1	404	uORF [213,326]	ATGTTTCTATGACCAAAGAACACAGTAATCTTCGTTTAGCTTCTGCTCAATAACACAAAAATCAGAAATTGCCGCTTCTGCTCTCAAAAGCTGCAATCAAAAGATGACATA TAA
AT3G11720.1	192	uORF [12,89]	ATGTATATTCCCGAGAAAATGAGGGAAAGTATCTTCTAATCGGGAAAAACATCTCCACGTTCCTTGCCCGTCTCG TAG
AT1G80420.1	224	uORF [21,146]	ATGATTTCGATCAAAGAGAAGCCACGGGGGGAGCGCTAGCACTTAAAGATTTGCACCGTTTTCTCTCTGGCTTTGTGTTGTGCTAGATGCGATGCAATAGCAATATTTTCCTGGAGTTTCAAG TAA

AT4G26890.1	233	uORF [35,124]	ATGTCGTATCCGACTTCGAACCAAAGCCCCATGCCAAAGTCACATACACACACTTCTACATACCCTAAACACACACACTCGAATACA TAA
AT3G09035.1	247	uORF [43,138]	ATGTCTCTGCTTTCTCCAGAAGATGATATTTACTTTTTGTGTCAATTAGCTGTATAACGTCTTCTTCACATAAAACAAGAACACTCCACTTAC TAA
AT5G08430.1	274	uORF [171,254]	ATGTATTCGCCTTTCATTGCAGTAGTAGGGTTTATAGTTTCTTAAGCTAGAGGTGTGAGTTTTTTTCTTCTCCTTAGGTTT TGA
AT5G02010.1	340	uORF [61,285]	ATGTTGAAGATGCAGAGAGATCGAGGAAGACAGAGCAAGAGAACCTCTTCACTCACAGACCCACTCACTGCATTTTCTCTTCTTTTCTCAGAAAATACTTTTTTCCCCCGAGAAAATGTTCCACAAGAATCAGATCGAACAATAAGGGTTCTCATAAATGTAATGCGATTCAAGCACGAGAATATCCACAGAGAGAGATACAGAAGGCTCCTTTTGAGTATT TGA
**AT1G51620.1**	406	uORF [35,169]	ATGGTGTGTATTGGTGTTTCTAGGAAACAGATAAACCAGTTTGCATTCTAGGCGATGACAGAAGCGACAGGCCTGAGGCGTTTCTTTTCTAAGTTCATTTGCTGTGTGTCCAATGCACAAGATCCATTTTTA TGA
AT1G51620.1	406	uORF [237,365]	ATGCAGACGATTCGACTATTGAATATGAACCATATGTGTACATTCCAGCGAATAGTTACTCGTATGCCGAGGTTACGAAAATTACAAACAAGTTTAATAGAGTTCATGGCAAAGGAGGGTTTGGTG TAG
AT4G25320.1	411	uORF [141,227]	ATGTATGTTTTAGGTCGAATTTTCTGAAATTAAGATTCATTCCTCCATGGAAGAAGCTCTGTTTTTATTCTCTTTAGCTTAGCT TAG
**AT5G10930.1**	208	uORF [53,148]	ATGGAATTGCTCTACTTCTTCACCTATGAGAGTAATATATCCTCATGTACTCAAAATACTAATCAAACTTCATGCTTCAACACCACCTTTTCT TAA
AT5G14740.1	285	uORF [27,125]	ATGTTTTTGTTTAATCAACAAGAGGCGGAGATACGGGAGAAATTGCATGTGTAATCATAAAATGTAGATGTTAGCTTCGTCGTTTTTACTATAGTT TAG
AT2G07727.1	285	uORF [41,151]	ATGAATCTAAGAAATTTAGGTCTCTGCCCGCTTGAAAGATTCTTCTTTCCTTTTCGGTGAAAGAGGGCAAAAGTGTGTAGGAGAAAGAATTCTAAAAACGTCGACGCT TAA

*Genes highlighted in bold represents that they have strong kozak signal

Supplemental Table 3: *De novo* motifs of length 8 found in the 3' UTRs of genes that belong to NYdown group

		***De novo* motif**				
**Motif ID**[1]	**Software**[2]	**Forward**[3]	**Reverse**[4]	**Occurence in the group**[5]	**Occurence in the whole genome**[6]	**Possible function / Matches in RNA motif database - All species (Ray et al., 2013)**[7]
S10	Seeder	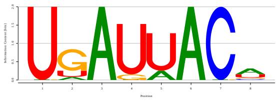	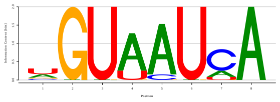	51 / 241	657 / 20346	-
S11	Seeder	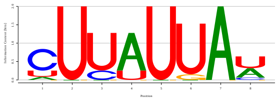	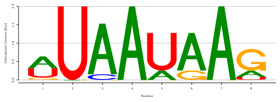	126 / 241	1625 / 20346	-
S12	Seeder	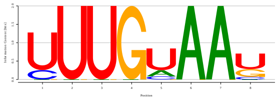	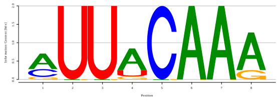	125 / 241	2509 / 20346	-
S13	Seeder	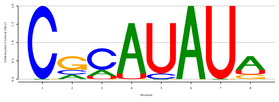	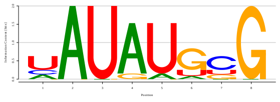	47 / 241	112 / 20346	Matches Bruno motif, regulation of translation
S14	Seeder	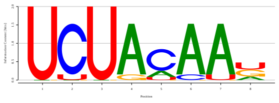	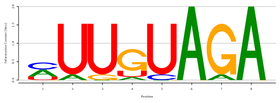	48 / 241	498 / 20346	-
S15	Seeder	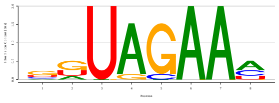	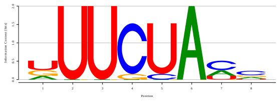	59 / 241	234 / 20346	-
S16	Seeder	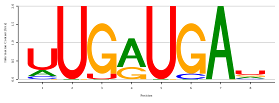	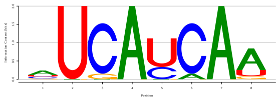	128 / 241	2368 / 20346	-
S17	Seeder	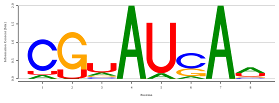	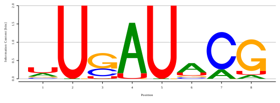	28 / 241	460 / 20346	-
S18	Seeder	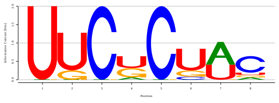	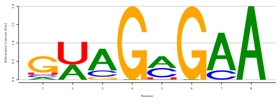	35 / 241	405 / 20346	-
S19	Seeder	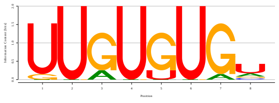	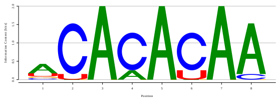	104 / 241	1476 / 20346	Simialr to Pumilio and Bruno motif, translation repression; RNCMPT00011 (PAPI),RNCMPT00166 (BRUNOL5),RNCMPT00003 (ARET),RNCMPT00270 (ARET),RNCMPT00004 (BRUNOL4)

S1	Seeder	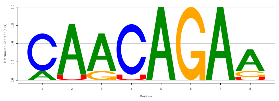	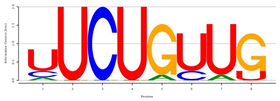	79 / 241	798 / 20346	-
S20	Seeder	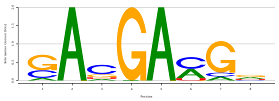	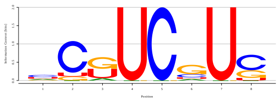	39 / 241	175 / 20346	RNCMPT00073 (SRSF7), RNCMPT00241 (RBM45)
S21	Seeder	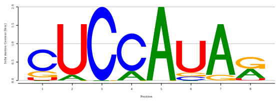	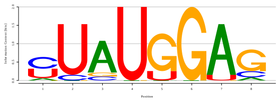	40 / 241	278 / 20346	-
S22	Seeder	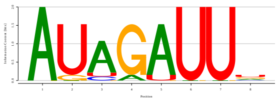	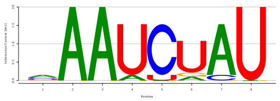	82 / 241	761 / 20346	-
S23	Seeder	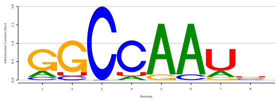	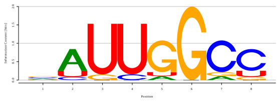	42 / 241	231 / 20346	-
S24	Seeder	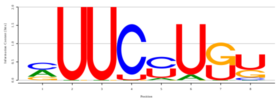	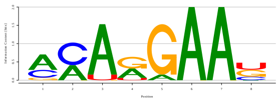	115 / 241	351 / 20346	-
S25	Seeder	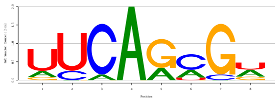	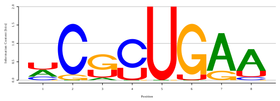	46 / 241	160 / 20346	-
S26	Seeder	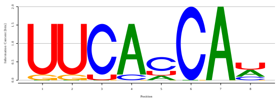	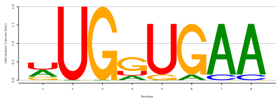	64 / 241	805 / 20346	-
S27	Seeder	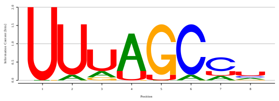	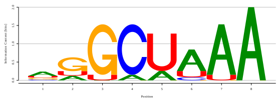	49 / 241	424 / 20346	-
S28	Seeder	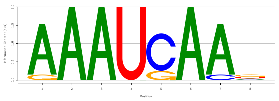	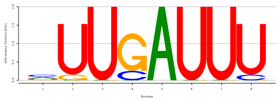	117 / 241	493 / 20346	-
S29	Seeder	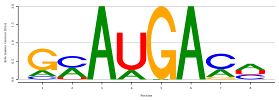	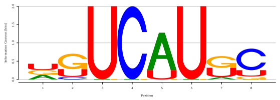	52 / 241	209 / 20346	-

S2	Seeder	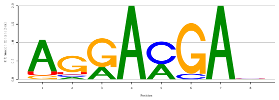	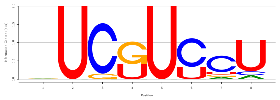	56 / 241	98 / 20346	-
S30	Seeder	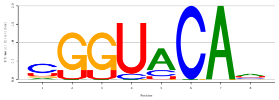	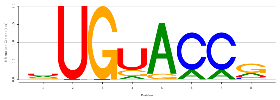	47 / 241	255 / 20346	-
S31	Seeder	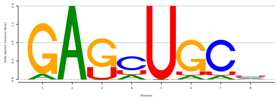	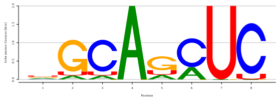	19 / 241	151 / 20346	-
S32	Seeder	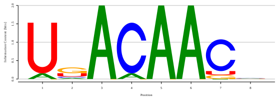	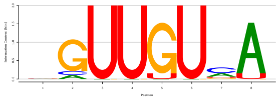	76 / 241	390 / 20346	-
S33	Seeder	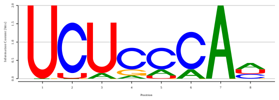	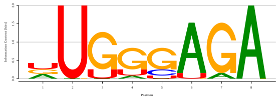	54 / 241	428 / 20346	-
S34	Seeder	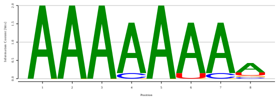	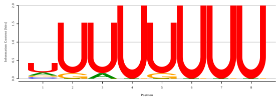	463 / 241	12492 / 20346	RNCMPT00043 (PABPC4)
S35	Seeder	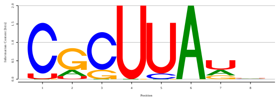	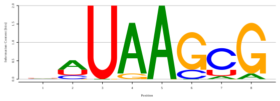	45 / 241	409 / 20346	-
S36	Seeder	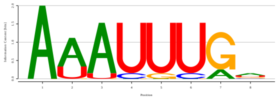	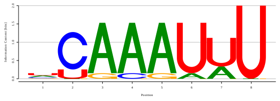	173 / 241	2757 / 20346	-
S37	Seeder	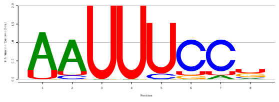	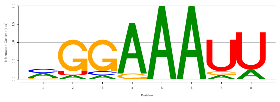	38 / 241	373 / 20346	-
S38	Seeder	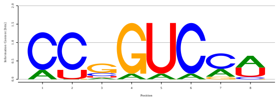	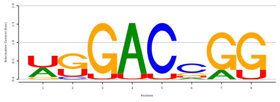	18 / 241	80 / 20346	-
S39	Seeder	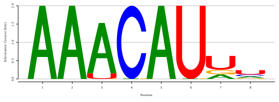	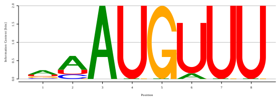	134 / 241	5148 / 20346	-

S3	Seeder	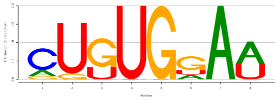	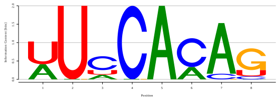	42 / 241	198 / 20346	Matches Bruno motif, regulation of translation
S40	Seeder	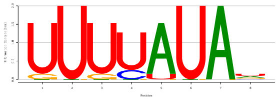	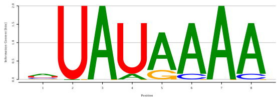	155 / 241	5142 / 20346	-
S41	Seeder	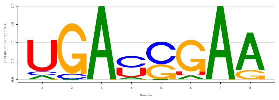	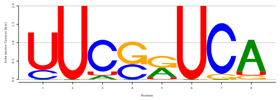	36 / 241	115 / 20346	
S42	Seeder	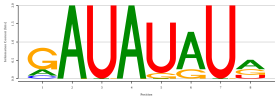	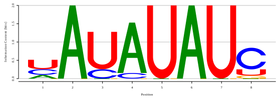	117 / 241	1184 / 20346	Matches Bruno motif, regulation of translation
S43	Seeder	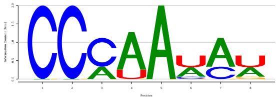	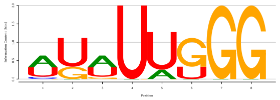	138 / 241	2754 / 20346	-
S44	Seeder	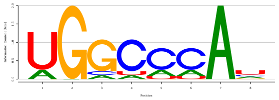	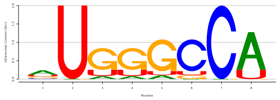	34 / 241	168 / 20346	-
S45	Seeder	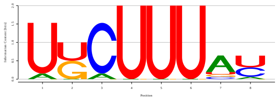	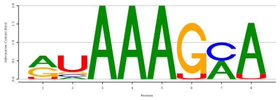	70 / 241	989 / 20346	-
S46	Seeder	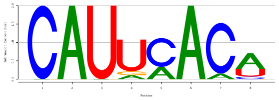	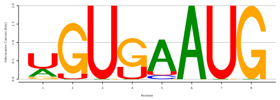	47 / 241	556 / 20346	Matches Pumilio motif, translation repression; RNCMPT00251 (Tb$_0$251)
S47	Seeder	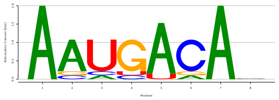	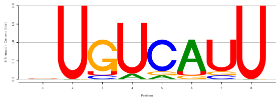	76 / 241	720 / 20346	RNCMPT00161 (FXR1)
S48	Seeder	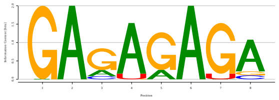	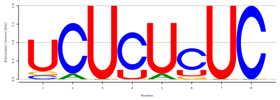	56 / 241	1498 / 20346	-
S49	Seeder	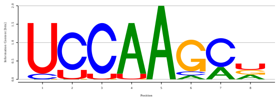	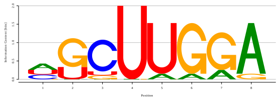	33 / 241	303 / 20346	-

S4	Seeder	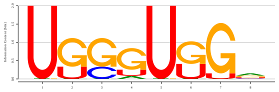	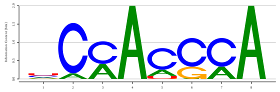	37 / 241	179 / 20346	-
S50	Seeder	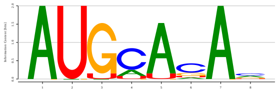	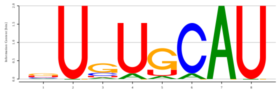	48 / 241	369 / 20346	Matches Bruno motif, translation regulation; RNCMPT00181 (MEC-8),RNCMPT00133 (CPO),RNCMPT00071 (SNRPA)
S51	Seeder	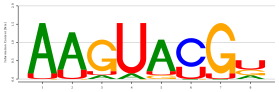	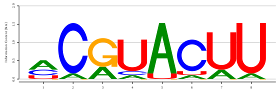	30 / 241	150 / 20346	-
S52	Seeder	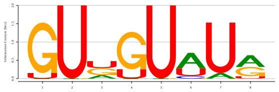	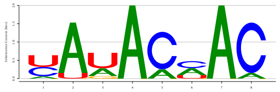	66 / 241	474 / 20346	-
S53	Seeder	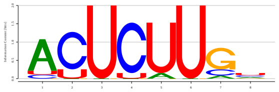	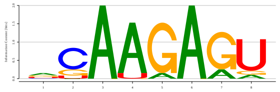	45 / 241	497 / 20346	-
S54	Seeder	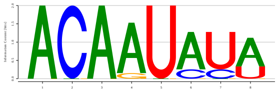	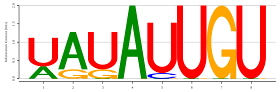	58 / 241	1481 / 20346	-
S55	Seeder	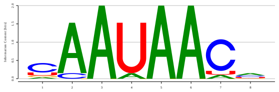	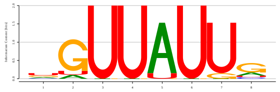	96 / 241	676 / 20346	-
S56	Seeder	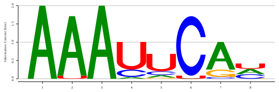	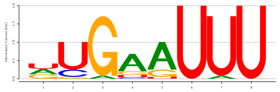	331 / 241	2423 / 20346	-
S5	Seeder	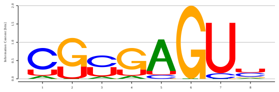	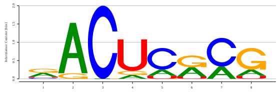	5 / 241	8 / 20346	-
S6	Seeder	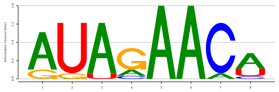	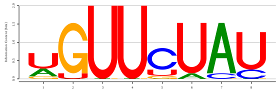	101 / 241	718 / 20346	-
S7	Seeder	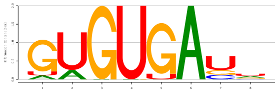	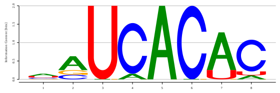	51 / 241	1469 / 20346	-

S8	Seeder	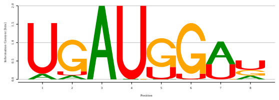	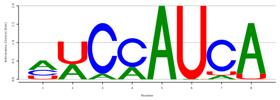	65 / 241	923 / 20346	-
S9	Seeder	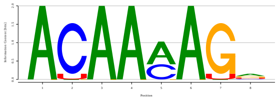	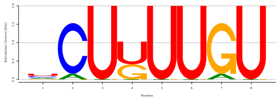	74 / 241	2645 / 20346	-
1	MEME	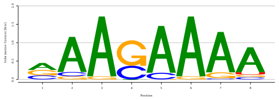	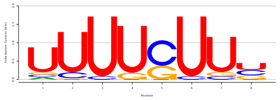	182 / 241	13219 / 20346	-
2	MEME	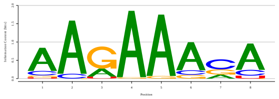	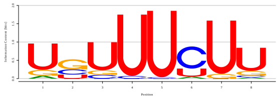	53 / 241	3931 / 20346	RNCMPT00064 (SART3), RNCMPT00155 (PABPC1)
3	MEME	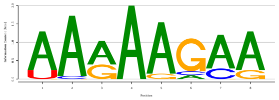	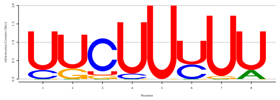	128 / 241	7311 / 20346	-
4	MEME	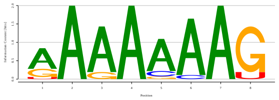	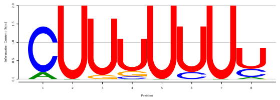	50 / 241	3862 / 20346	RNCMPT00042 (Nab2p), RNCMPT00289 (Hnrnpr)
5	MEME	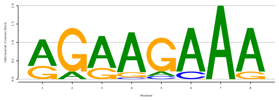	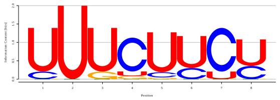	58 / 241	3237 / 20346	-
6	MEME	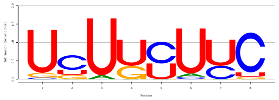	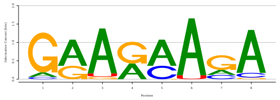	54 / 241	3061 / 20346	Similar to Bruno and Pumilio motif, translation repression
W1	Weeder	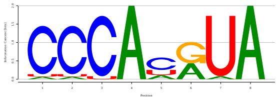	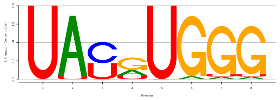	16 / 241	630 / 20346	-

[1]Name of the motif
[2]*de novo* discovery software that was used to locate the motif
[3]Forward sequence of the motif; Motif logo representing the occurence of a specific nucleotide at a respective position. The x-axis represents the position of a nucleotide and y-axis represents the amount of information in bits
[4]Reverse sequence of the motif; Motif logo representing the occurence of a specific nucleotide at a respective position.The x-axis represents the position of a nucleotide and y-axis represents the amount of information in bits
[5]Number of 3' UTRs containing the motif in the specific group. Note: Motif might occur more than once per sequence
[6]Number of 3' UTRs containing the motif in the whole Arabidopsis genome. Note: Motif might occur more than once per sequence
[7]Motif match in the database / literature

Supplemental Table 4: *De novo* motifs of length 8 found in the 3' UTRs of genes that belong to NYup group

		***De novo* motif**				
**Motif ID**[1]	**Software**[2]	**Forward**[3]	**Reverse**[4]	**Occurence in the group**[5]	**Occurence in the whole genome**[6]	**Possible function / Matches in RNA motif database - All species (Ray et al., 2013)**[7]
S10	Seeder	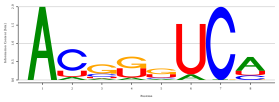	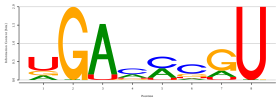	11 / 85	1816 / 20346	-
S11	Seeder	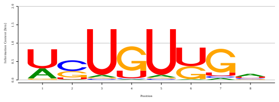	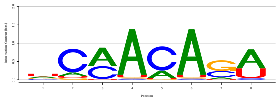	77 / 85	10766 / 20346	-
S12	Seeder	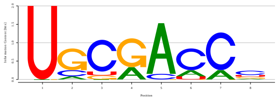	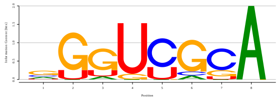	7 / 85	701 / 20346	-
S13	Seeder	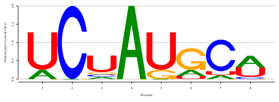	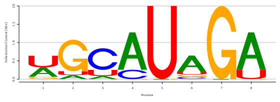	21 / 85	2962 / 20346	Matches Pumilio motif, translation repression
S14	Seeder	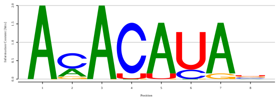	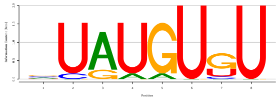	59 / 85	9676 / 20346	Matches Bruno motif, translation regulation; RNCMPT00008 (CNOT4)
S15	Seeder	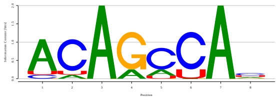	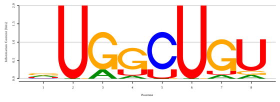	14 / 85	2150 / 20346	Matches Bruno motif, regulation of translation
S16	Seeder	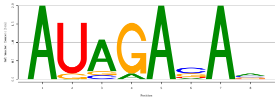	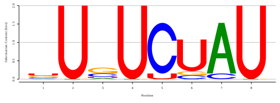	58 / 85	9875 / 20346	Matches Bruno motif, regulation of translation
S17	Seeder	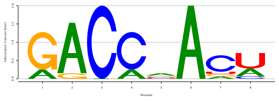	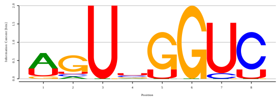	32 / 85	4459 / 20346	-
S18	Seeder	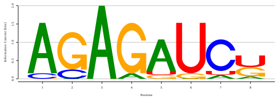	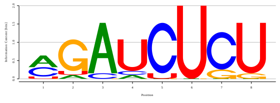	18 / 85	2729 / 20346	-
S19	Seeder	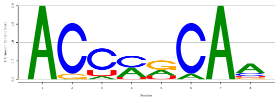	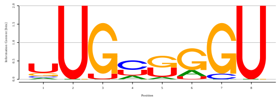	17 / 85	2900 / 20346	Matches Bruno motif, regulation of translation

S1	Seeder	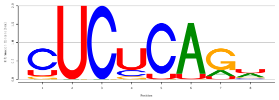	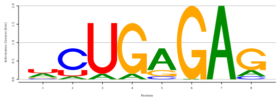	19 / 85	4415 / 20346	-
S20	Seeder	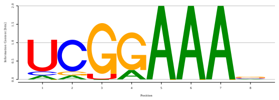	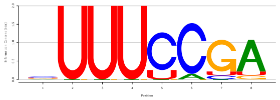	16 / 85	2014 / 20346	-
S21	Seeder	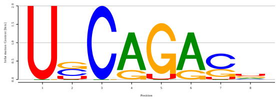	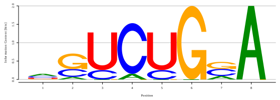	18 / 85	3209 / 20346	-
S22	Seeder	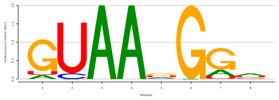	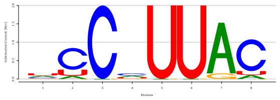	17 / 85	2514 / 20346	-
S23	Seeder	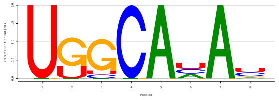	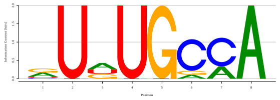	18 / 85	2326 / 20346	-
S24	Seeder	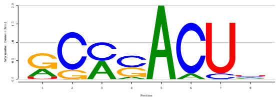	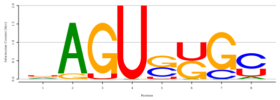	14 / 85	2832 / 20346	-
S25	Seeder	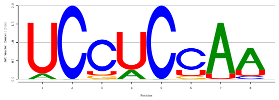	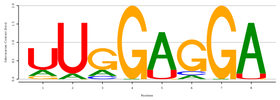	12 / 85	2108 / 20346	-
S26	Seeder	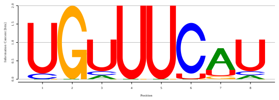	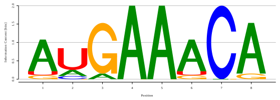	62 / 85	6369 / 20346	-
S27	Seeder	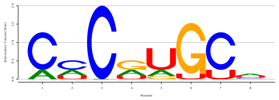	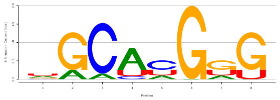	15 / 85	1718 / 20346	-
S28	Seeder	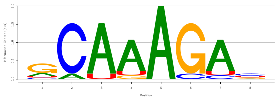	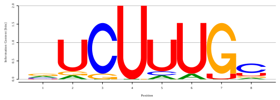	45 / 85	5772 / 20346	-
S29	Seeder	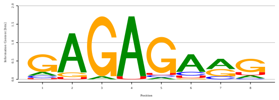	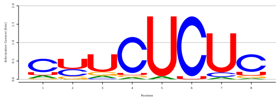	70 / 85	12919 / 20346	RNCMPT00060 (RNP4F)

S2	Seeder	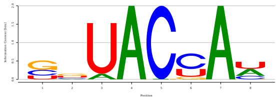	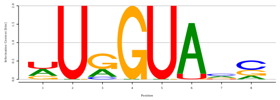	32 / 85	5376 / 20346	-
S30	Seeder	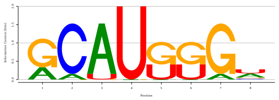	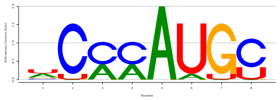	17 / 85	2822 / 20346	-
S31	Seeder	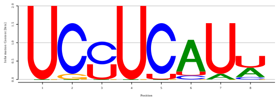	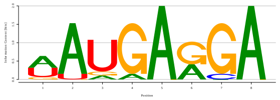	27 / 85	4888 / 20346	-
S32	Seeder	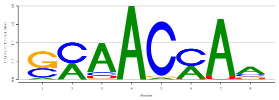	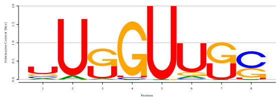	113 / 85	18252 / 20346	-
S33	Seeder	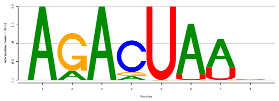	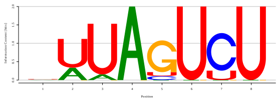	16 / 85	4072 / 20346	-
S34	Seeder	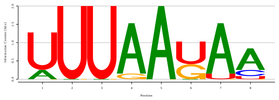	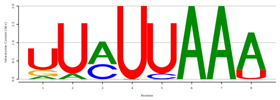	40 / 85	6751 / 20346	-
S35	Seeder	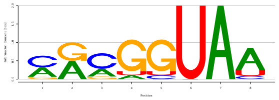	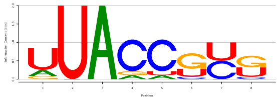	10 / 85	1417 / 20346	-
S36	Seeder	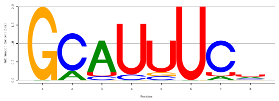	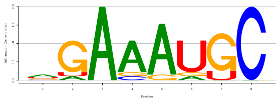	21 / 85	3351 / 20346	-
S37	Seeder	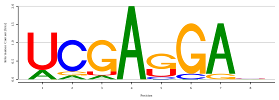	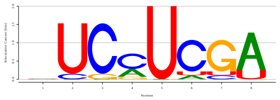	15 / 85	1878 / 20346	-
S38	Seeder	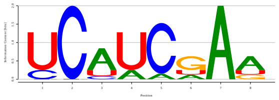	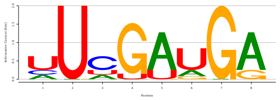	34 / 85	4778 / 20346	-
S39	Seeder	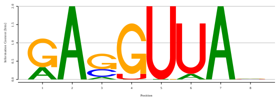	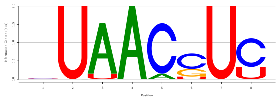	25 / 85	2618 / 20346	-

S3	Seeder	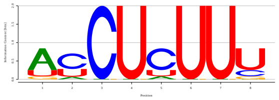	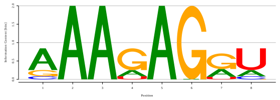	28 / 85	5349 / 20346	-
S40	Seeder	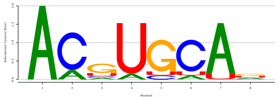	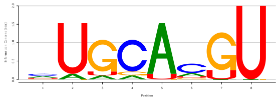	11 / 85	1497 / 20346	-
S41	Seeder	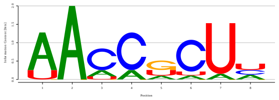	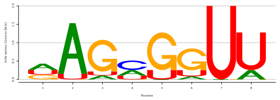	17 / 85	2546 / 20346	-
S42	Seeder	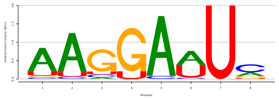	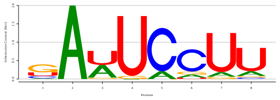	38 / 85	8148 / 20346	-
S43	Seeder	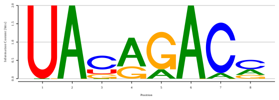	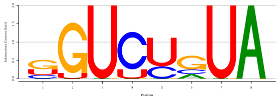	17 / 85	1822 / 20346	Matches Pumilio motif, translation repression
S44	Seeder	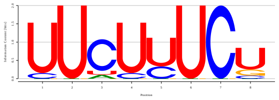	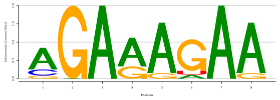	49 / 85	8049 / 20346	-
S45	Seeder	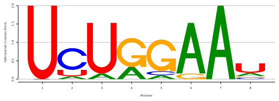	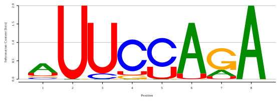	17 / 85	3290 / 20346	-
S46	Seeder	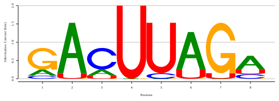	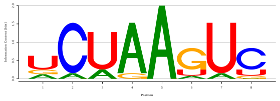	10 / 85	2175 / 20346	-
S47	Seeder	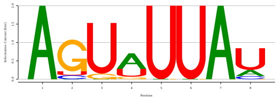	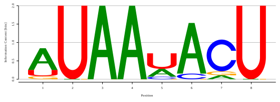	28 / 85	4378 / 20346	-
S48	Seeder	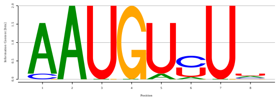	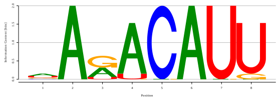	34 / 85	7331 / 20346	-
S49	Seeder	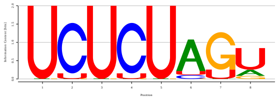	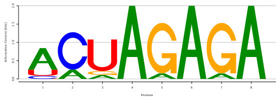	16 / 85	2066 / 20346	-

S4	Seeder	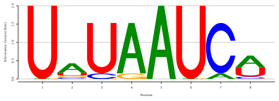	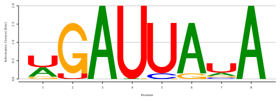	33 / 85	6096 / 20346	-
S50	Seeder	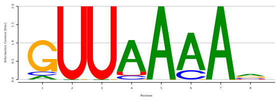	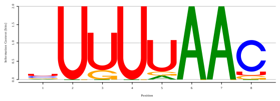	37 / 85	7078 / 20346	-
S51	Seeder	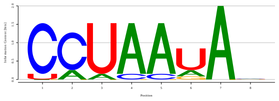	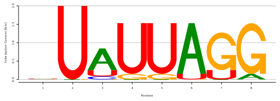	22 / 85	4249 / 20346	-
S52	Seeder	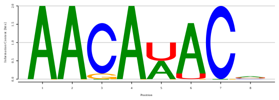	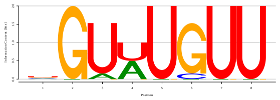	67 / 85	9153 / 20346	Matches Bruno motif, translation regulation; RNCMPT00033 (IGF2BP2),RNCMPT00172 (IGF2BP3)
S53	Seeder	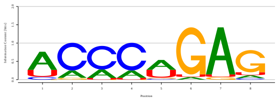	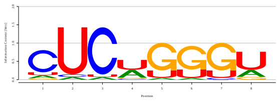	20 / 85	4175 / 20346	Matches Bruno motif, regulation of translation
S54	Seeder	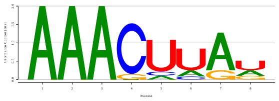	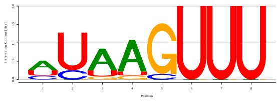	105 / 85	17627 / 20346	-
S55	Seeder	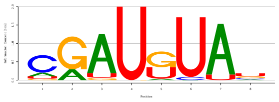	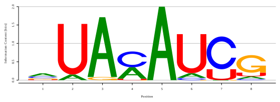	19 / 85	4958 / 20346	-
S56	Seeder	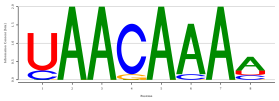	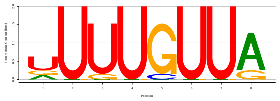	56 / 85	8935 / 20346	-
S58	Seeder	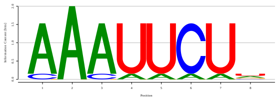	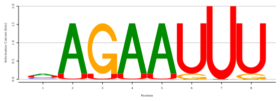	45 / 85	10448 / 20346	-
S59	Seeder	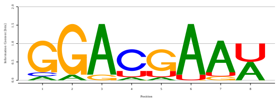	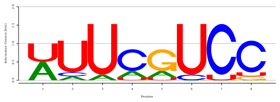	21 / 85	2755 / 20346	-
S5	Seeder	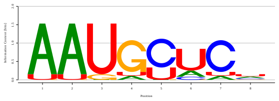	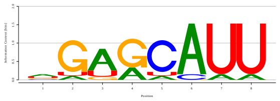	26 / 85	3372 / 20346	-

S61	Seeder	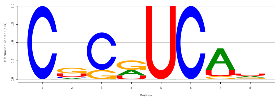	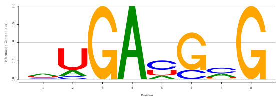	17 / 85	2747 / 20346	-
S62	Seeder	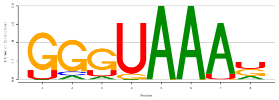	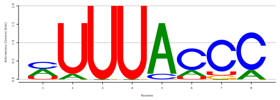	16 / 85	2100 / 20346	-
S6	Seeder	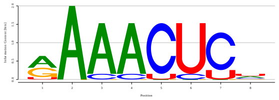	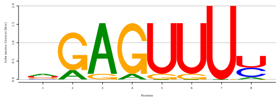	52 / 85	9649 / 20346	-
S7	Seeder	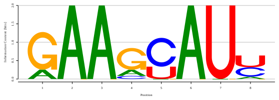	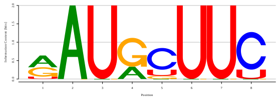	30 / 85	2809 / 20346	-
S8	Seeder	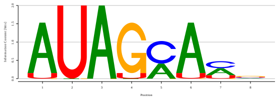	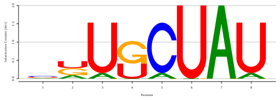	17 / 85	3318 / 20346	-
S9	Seeder	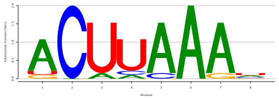	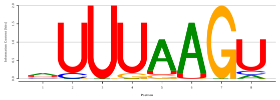	62 / 85	11917 / 20346	-
M1	MEME	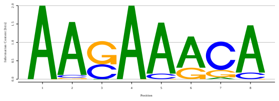	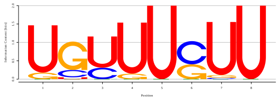	43 / 85	4252 / 20346	Matches Bruno motif, regulation of translation
M2	MEME	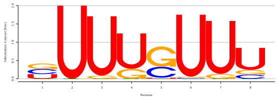	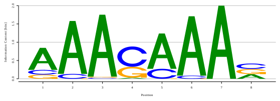	68 / 85	7193 / 20346	-
M3	MEME	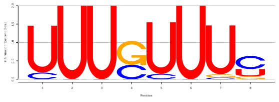	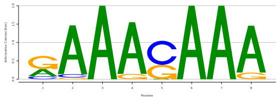	26 / 85	3169 / 20346	-
W1	Weeder	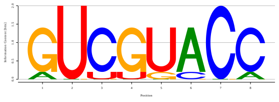	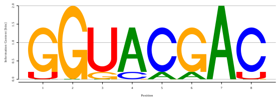	8 / 85	438 / 20346	-

[1]Name of the motif
[2]*de novo* discovery software that was used to locate the motif
[3]Forward sequence of the motif; Motif logo representing the occurence of a specific nucleotide at a respective position. The x-axis represents the position of a nucleotide and y-axis represents the amount of information in bits
[4]Reverse sequence of the motif; Motif logo representing the occurence of a specific nucleotide at a respective position.The x-axis represents the position of a nucleotide and y-axis represents the amount of information in bits
[5]Number of 3' UTRs containing the motif in the specific group. Note: Motif might occur more than once per sequence
[6]Number of 3' UTRs containing the motif in the whole Arabidopsis genome. Note: Motif might occur more than once per sequence
[7]Motif match in the database / literature

Supplemental Table 5: *De novo* motifs of length 8 found in the 3' UTRs of genes that belong to YNdown group

		***De novo* motif**				
**Motif ID**[1]	**Software**[2]	**Forward**[3]	**Reverse**[4]	**Occurence in the group**[5]	**Occurence in the whole genome**[6]	**Possible function / Matches in RNA motif database - All species (Ray et al., 2013)**[7]
S10	Seeder	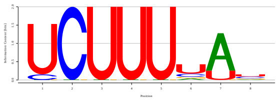	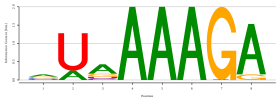	67 / 54	14715 / 20346	-
S11	Seeder	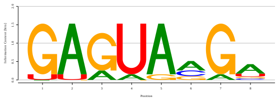	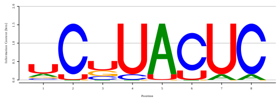	17 / 54	3657 / 20346	-
S12	Seeder	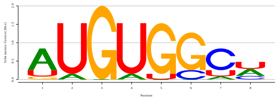	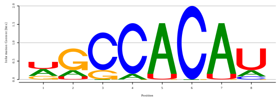	8 / 54	2565 / 20346	-
S13	Seeder	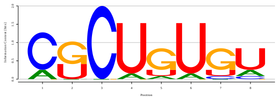	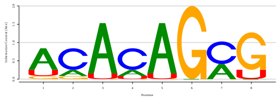	4 / 54	1410 / 20346	Matches Bruno motif, regulation of translation
S14	Seeder	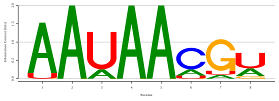	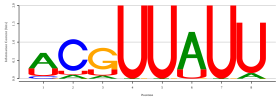	20 / 54	4787 / 20346	-
S15	Seeder	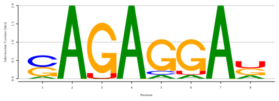	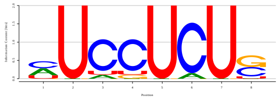	16 / 54	3958 / 20346	-
S16	Seeder	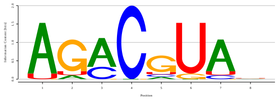	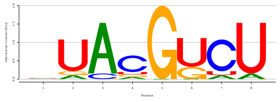	13 / 54	2118 / 20346	RNCMPT00002 (ANKHD1)
S17	Seeder	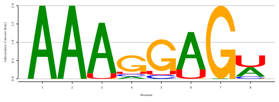	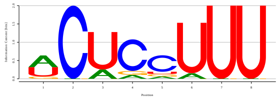	29 / 54	7276 / 20346	-
S18	Seeder	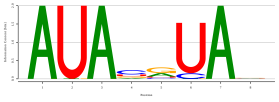	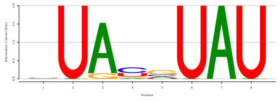	29 / 54	6863 / 20346	Matches Bruno motif, regulation of translation
S19	Seeder	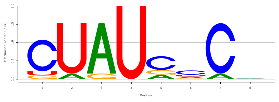	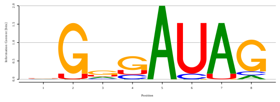	7 / 54	1687 / 20346	-

S1	Seeder	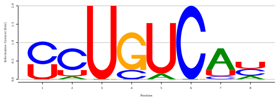	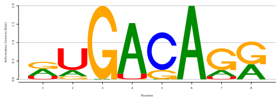	12 / 54	2389 / 20346	RNCMPT00246 (Pcbp2)
S20	Seeder	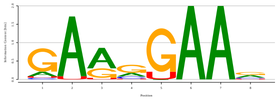	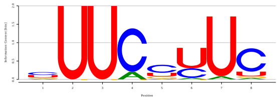	46 / 54	13800 / 20346	RNCMPT00055 (RBM5)
S21	Seeder	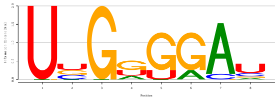	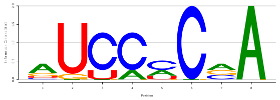	21 / 54	3773 / 20346	RNCMPT00150 (ESRP2)
S22	Seeder	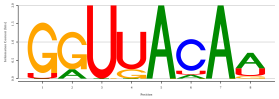	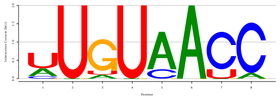	13 / 54	3081 / 20346	-
S23	Seeder	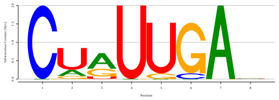	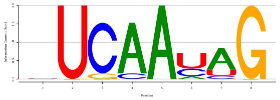	28 / 54	5582 / 20346	-
S24	Seeder	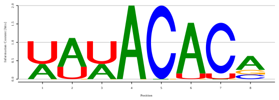	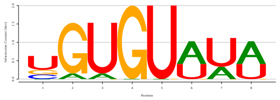	31 / 54	7839 / 20346	Similar to Bruno and Pumilio motif, translation process
S25	Seeder	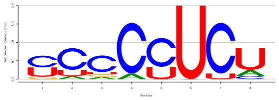	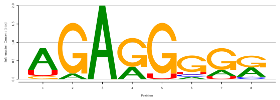	7 / 54	1458 / 20346	-
S27	Seeder	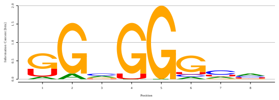	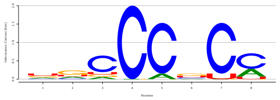	6 / 54	735 / 20346	-
S28	Seeder	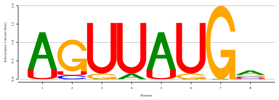	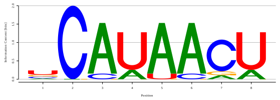	22 / 54	4543 / 20346	-
S29	Seeder	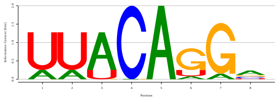	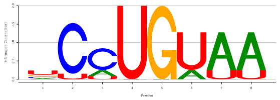	19 / 54	3800 / 20346	-
S2	Seeder	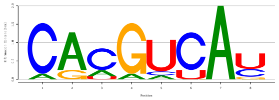	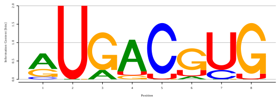	14 / 54	1810 / 20346	-

S3	Seeder	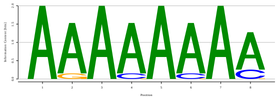	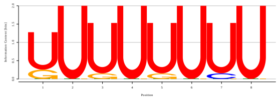	110 / 54	28345 / 20346	RNCMPT00043 (PABPC4)
S4	Seeder	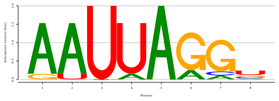	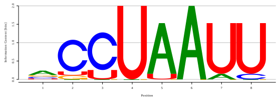	19 / 54	3407 / 20346	-
S5	Seeder	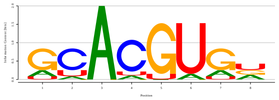	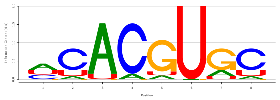	7 / 54	1099 / 20346	-
S6	Seeder	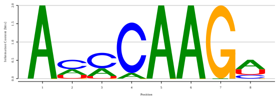	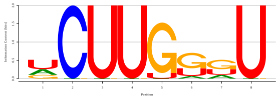	21 / 54	5712 / 20346	-
S7	Seeder	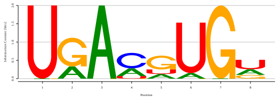	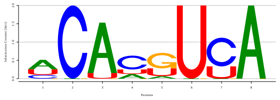	13 / 54	2650 / 20346	-
S8	Seeder	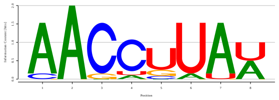	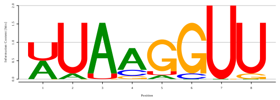	19 / 54	4908 / 20346	-
S9	Seeder	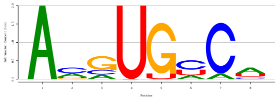	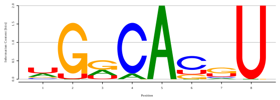	15 / 54	1886 / 20346	-
W1	Weeder	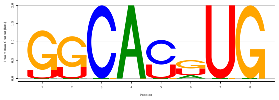	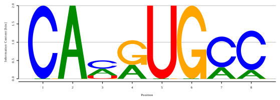	10 / 54	848 / 20346	-
M1	MEME	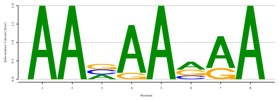	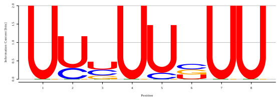	77 / 54	21635 / 20346	RNCMPT00064 (SART3),RNCMPT00155 (PABPC1),RNCMPT00253 (Tb$_0$253)
M2	MEME	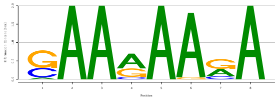	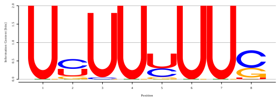	49 / 54	9820 / 20346	-

[1]Name of the motif

[2]*de novo* discovery software that was used to locate the motif

[3]Forward sequence of the motif; Motif logo representing the occurence of a specific nucleotide at a respective position. The x-axis represents the position of a nucleotide and y-axis represents the amount of information in bits

[4]Reverse sequence of the motif; Motif logo representing the occurence of a specific nucleotide at a respective position.The x-axis represents the position of a nucleotide and y-axis represents the amount of information in bits

[5]Number of 3' UTRs containing the motif in the specific group. Note: Motif might occur more than once per sequence

[6]Number of 3' UTRs containing the motif in the whole Arabidopsis genome. Note: Motif might occur more than once per sequence

[7]Motif match in the database / literature

Supplemental Table 6: *De novo* motifs of length 8 found in the 3’ UTRs of genes that belong to YNup group

		***De novo* motif**				
**Motif ID**[1]	**Software**[2]	**Forward**[3]	**Reverse**[4]	**Occurence in the group**[5]	**Occurence in the whole genome**[6]	**Possible function / Matches in RNA motif database - All species (Ray et al., 2013)**[7]
S1	Seeder	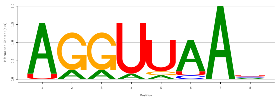	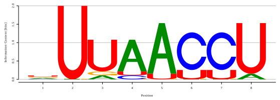	24 / 59	4418 / 20346	-
S2	Seeder	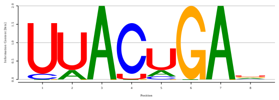	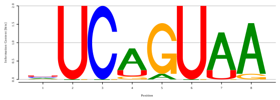	21 / 59	4078 / 20346	-
S3	Seeder	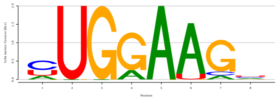	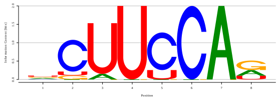	11 / 59	2933 / 20346	-
S4	Seeder	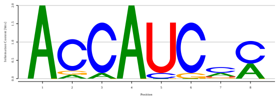	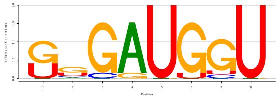	8 / 59	1556 / 20346	RNCMPT00026 (HNRNPK)
S5	Seeder	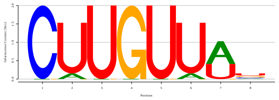	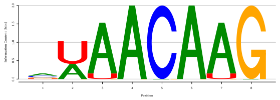	25 / 59	5869 / 20346	-
S6	Seeder	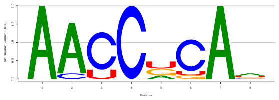	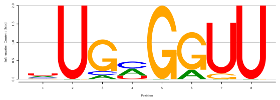	13 / 59	3610 / 20346	-
S7	Seeder	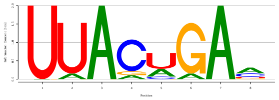	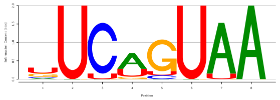	18 / 59	3489 / 20346	-
W1	Weeder	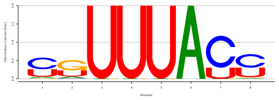	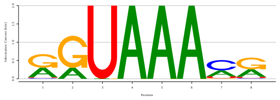	18 / 59	2341 / 20346	-

[1]Name of the motif
[2]*de novo* discovery software that was used to locate the motif
[3]Forward sequence of the motif; Motif logo representing the occurence of a specific nucleotide at a respective position. The x-axis represents the position of a nucleotide and y-axis represents the amount of information in bits
[4]Reverse sequence of the motif; Motif logo representing the occurence of a specific nucleotide at a respective position.The x-axis represents the position of a nucleotide and y-axis represents the amount of information in bits
[5]Number of 3’ UTRs containing the motif in the specific group. Note: Motif might occur more than once per sequence
[6]Number of 3’ UTRs containing the motif in the whole Arabidopsis genome. Note: Motif might occur more than once per sequence
[7]Motif match in the database / literature

Supplemental Table 7: *De novo* motifs of length 8 found in the 3' UTRs of genes that belong to YYdown group

		***De novo* motif**				
**Motif ID**[1]	**Software**[2]	**Forward**[3]	**Reverse**[4]	**Occurence in the group**[5]	**Occurence in the whole genome**[6]	**Possible function / Matches in RNA motif database - All species (Ray et al., 2013)**[7]
S10	Seeder	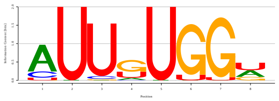	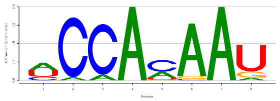	20 / 21	10853 / 20346	-
S11	Seeder	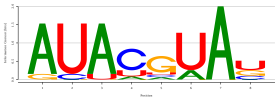	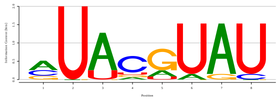	10 / 21	3927 / 20346	Matches Bruno motif, regulation of translation
S12	Seeder	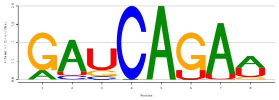	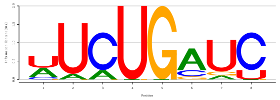	15 / 21	4508 / 20346	-
S13	Seeder	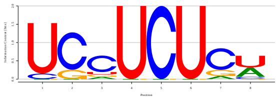	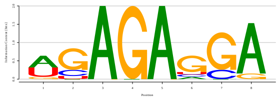	14 / 21	5081 / 20346	-
S14	Seeder	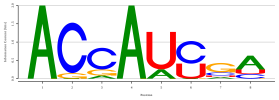	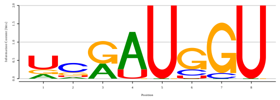	9 / 21	3528 / 20346	-
S16	Seeder	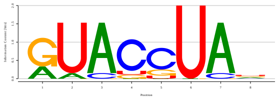	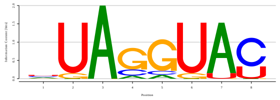	7 / 21	1711 / 20346	-
S18	Seeder	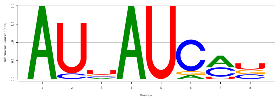	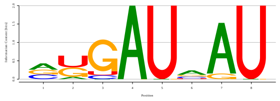	20 / 21	9540 / 20346	-
S19	Seeder	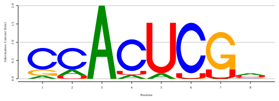	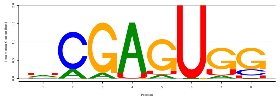	6 / 21	1391 / 20346	-
S1	Seeder	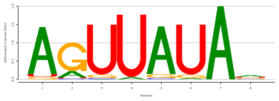	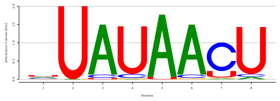	20 / 21	14253 / 20346	-
S20	Seeder	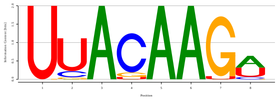	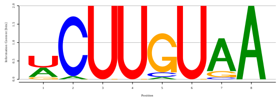	20 / 21	13311 / 20346	-

S21	Seeder	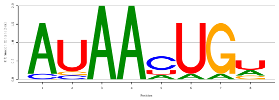	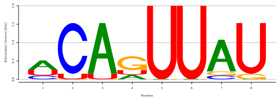	14 / 21	4794 / 20346	-
S22	Seeder	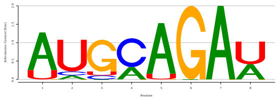	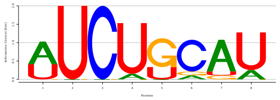	12 / 21	4347 / 20346	-
S2	Seeder	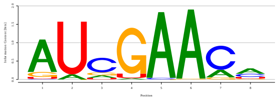	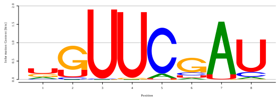	11 / 21	6830 / 20346	-
S3	Seeder	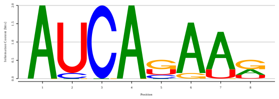	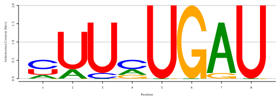	15 / 21	5403 / 20346	-
S4	Seeder	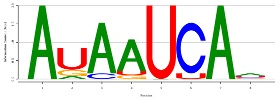	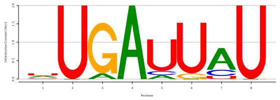	19 / 21	11705 / 20346	-
S5	Seeder	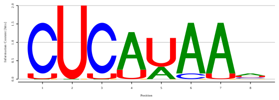	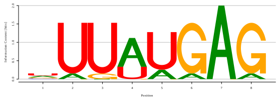	18 / 21	6259 / 20346	-
S6	Seeder	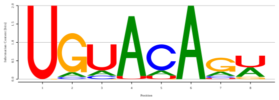	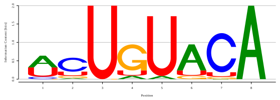	20 / 21	9117 / 20346	Matches Pumilio motif, translation repression; RNCMPT00101 (PUM),RNCMPT00102 (PUM),RNCMPT00046 (PUM)
S7	Seeder	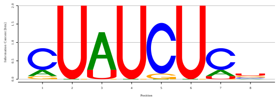	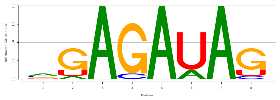	12 / 21	3682 / 20346	-
S8	Seeder	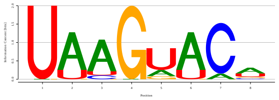	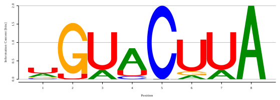	11 / 21	4570 / 20346	Matches Pumilio motif, translation repression
S9	Seeder	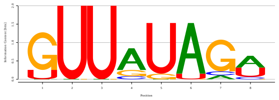	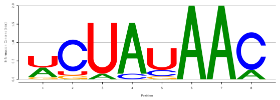	12 / 21	3703 / 20346	-
W1	Weeder	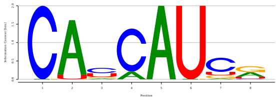	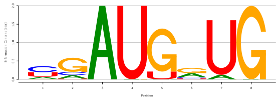	13 / 21	3231 / 20346	-

[1]Name of the motif
[2]*de novo* discovery software that was used to locate the motif
[3]Forward sequence of the motif; Motif logo representing the occurence of a specific nucleotide at a respective position. The x-axis represents the position of a nucleotide and y-axis represents the amount of information in bits
[4]Reverse sequence of the motif; Motif logo representing the occurence of a specific nucleotide at a respective position.The x-axis represents the position of a nucleotide and y-axis represents the amount of information in bits
[5]Number of 3' UTRs containing the motif in the specific group. Note: Motif might occur more than once per sequence
[6]Number of 3' UTRs containing the motif in the whole Arabidopsis genome. Note: Motif might occur more than once per sequence
[7]Motif match in the database / literature

Supplemental Table 8: *De novo* motifs of length 8 found in the 3' UTRs of genes that belong to YYup group

***De novo* motif**						
**Motif ID**[1]	**Software**[2]	**Forward**[3]	**Reverse**[4]	**Occurence in the group**[5]	**Occurence in the whole genome**[6]	**Possible function / Matches in RNA motif database - All species (Ray et al., 2013)**[7]
W1	Weeder	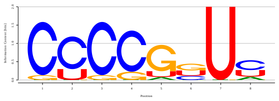	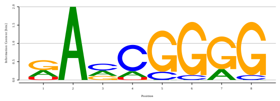	7 / 10	566 / 20346	-

[1]Name of the motif
[2]*de novo* discovery software that was used to locate the motif
[3]Forward sequence of the motif; Motif logo representing the occurence of a specific nucleotide at a respective position. The x-axis represents the position of a nucleotide and y-axis represents the amount of information in bits
[4]Reverse sequence of the motif; Motif logo representing the occurence of a specific nucleotide at a respective position.The x-axis represents the position of a nucleotide and y-axis represents the amount of information in bits
[5]Number of 3' UTRs containing the motif in the specific group. Note: Motif might occur more than once per sequence
[6]Number of 3' UTRs containing the motif in the whole Arabidopsis genome. Note: Motif might occur more than once per sequence
[7]Motif match in the database / literature

Supplemental Table 9: *De novo* motifs of length 8 found in the 5' UTRs of genes that belong to NYdown group

		***De novo* motif**				
**Motif ID**[1]	**Software**[2]	**Forward**[3]	**Reverse**[4]	**Occurence in the group**[5]	**Occurence in the whole genome**[6]	**Possible function / Matches in RNA motif database - All species (Ray et al., 2013)**[7]
S10	Seeder	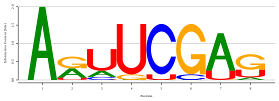	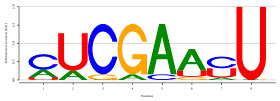	32 / 237	2623 / 19128	-
S11	Seeder	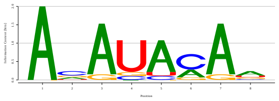	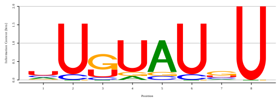	99 / 237	5967 / 19128	-
S12	Seeder	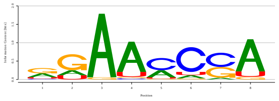	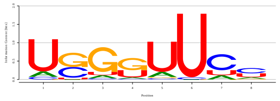	67 / 237	5435 / 19128	-
S13	Seeder	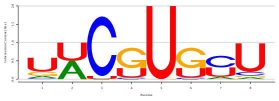	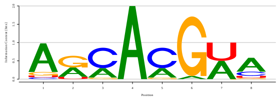	33 / 237	2754 / 19128	-
S14	Seeder	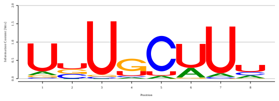	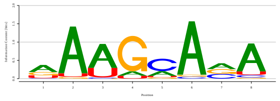	280 / 237	21460 / 19128	-
S15	Seeder	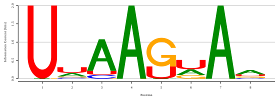	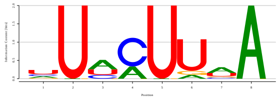	153 / 237	12156 / 19128	-
S16	Seeder	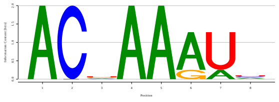	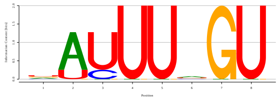	133 / 237	10820 / 19128	-
S17	Seeder	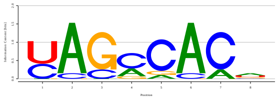	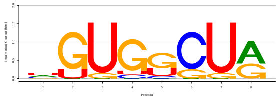	46 / 237	2994 / 19128	-
S18	Seeder	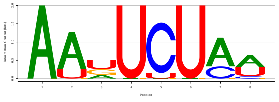	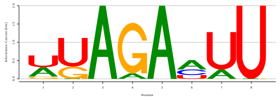	66 / 237	6026 / 19128	-
S19	Seeder	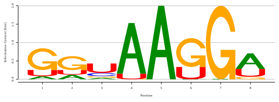	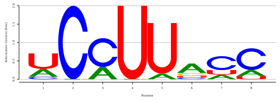	33 / 237	2335 / 19128	-

S1	Seeder	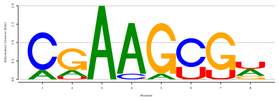	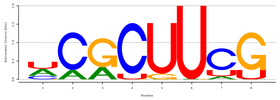	35 / 237	1662 / 19128	-
S20	Seeder	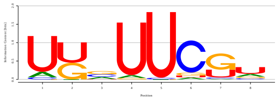	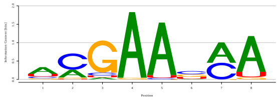	239 / 237	17199 / 19128	RNCMPT00164 (ZNF638)
S21	Seeder	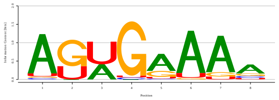	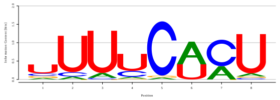	381 / 237	27130 / 19128	-
S22	Seeder	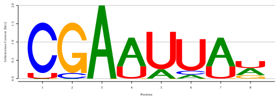	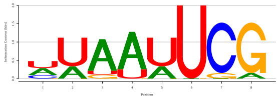	81 / 237	5173 / 19128	-
S23	Seeder	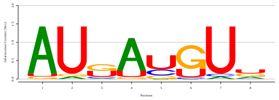	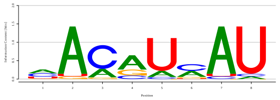	66 / 237	3612 / 19128	-
S24	Seeder	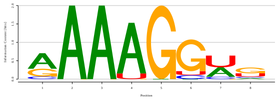	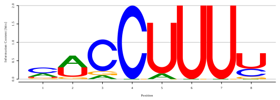	97 / 237	6026 / 19128	-
S25	Seeder	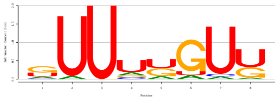	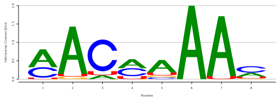	161 / 237	11943 / 19128	RNCMPT00132 (RBP9)
S26	Seeder	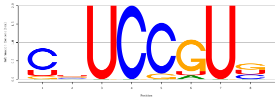	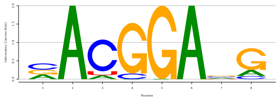	27 / 237	1953 / 19128	-
S27	Seeder	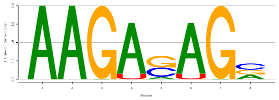	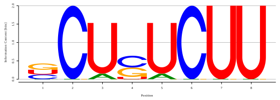	113 / 237	7517 / 19128	-
S28	Seeder	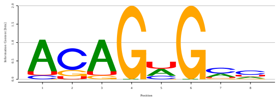	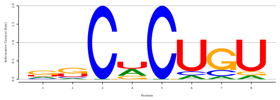	59 / 237	5027 / 19128	-
S29	Seeder	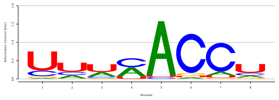	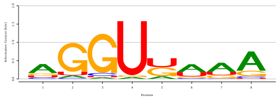	215 / 237	15106 / 19128	-

S2	Seeder	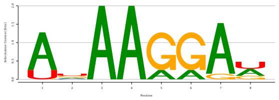	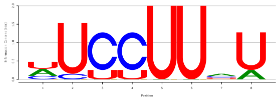	298 / 237	21549 / 19128	-
S30	Seeder	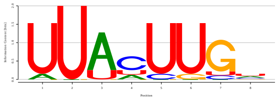	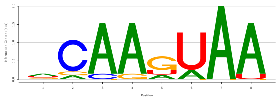	54 / 237	3560 / 19128	-
S31	Seeder	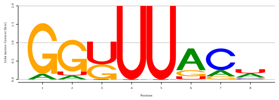	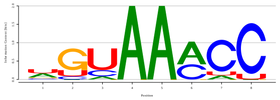	30 / 237	2251 / 19128	-
S32	Seeder	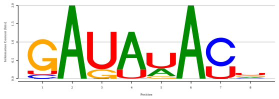	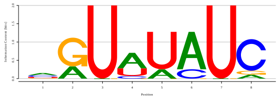	54 / 237	4298 / 19128	-
S34	Seeder	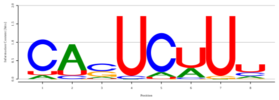	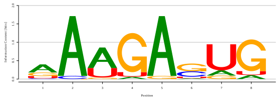	175 / 237	12507 / 19128	-
S35	Seeder	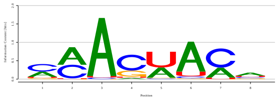	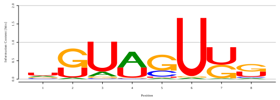	157 / 237	11320 / 19128	RNCMPT00282 (Rbm42)
S36	Seeder	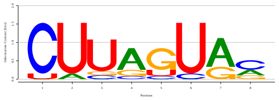	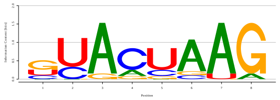	26 / 237	1617 / 19128	-
S37	Seeder	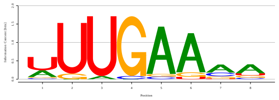	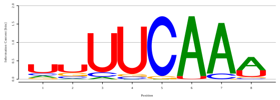	190 / 237	15941 / 19128	-
S38	Seeder	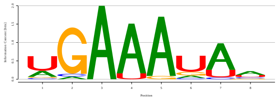	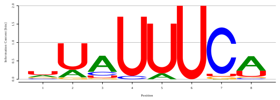	260 / 237	20037 / 19128	-
S39	Seeder	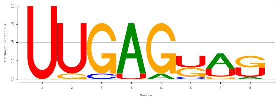	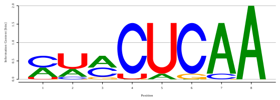	44 / 237	3528 / 19128	-
S3	Seeder	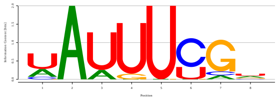	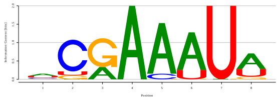	52 / 237	4285 / 19128	-

S40	Seeder	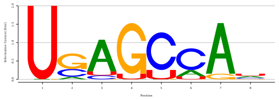	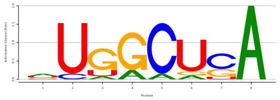	32 / 237	2132 / 19128	-
S41	Seeder	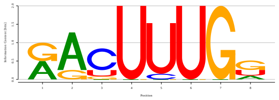	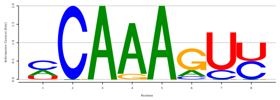	51 / 237	3615 / 19128	-
S42	Seeder	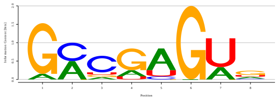	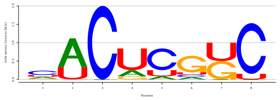	22 / 237	1947 / 19128	-
S43	Seeder	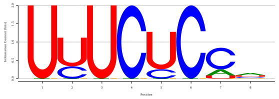	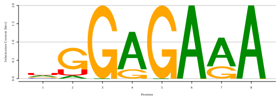	124 / 237	10461 / 19128	-
S44	Seeder	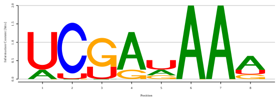	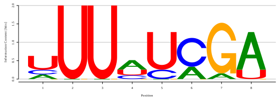	80 / 237	5476 / 19128	-
S45	Seeder	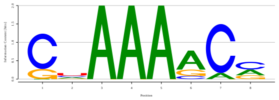	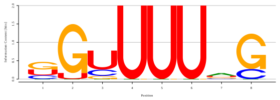	122 / 237	9103 / 19128	-
S46	Seeder	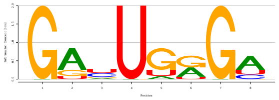	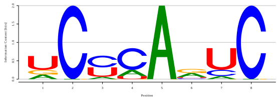	43 / 237	3088 / 19128	-
S47	Seeder	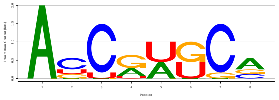	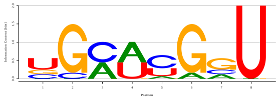	24 / 237	2116 / 19128	-
S48	Seeder	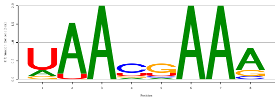	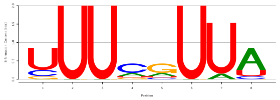	74 / 237	5313 / 19128	-
S49	Seeder	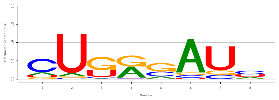	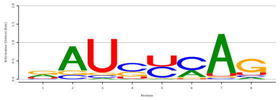	43 / 237	3914 / 19128	-
S4	Seeder	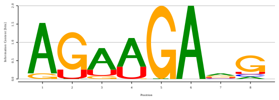	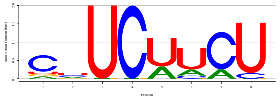	276 / 237	20878 / 19128	RNCMPT00078 (TRA2)

S50	Seeder	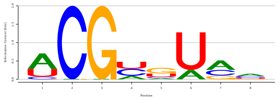	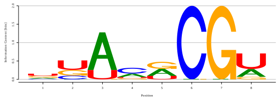	31 / 237	2874 / 19128	-
S51	Seeder	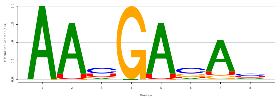	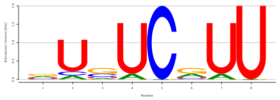	23 / 237	2799 / 19128	RNCMPT00161 (FXR1)
S52	Seeder	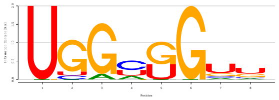	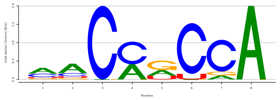	47 / 237	2214 / 19128	-
S53	Seeder	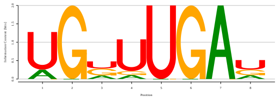	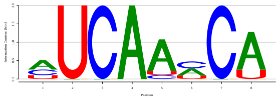	59 / 237	4529 / 19128	-
S54	Seeder	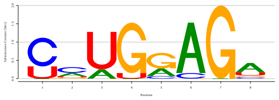	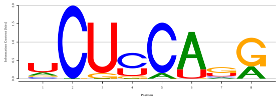	36 / 237	2912 / 19128	-
S55	Seeder	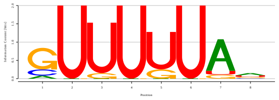	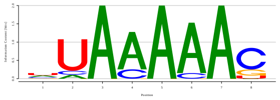	85 / 237	5784 / 19128	-
S56	Seeder	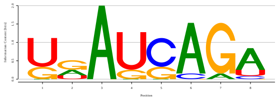	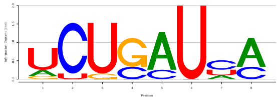	68 / 237	5558 / 19128	-
S57	Seeder	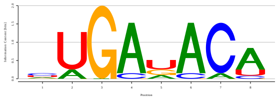	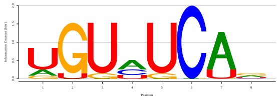	62 / 237	4736 / 19128	-
S5	Seeder	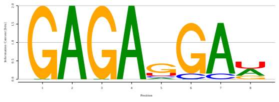	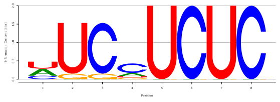	105 / 237	8238 / 19128	-
S6	Seeder	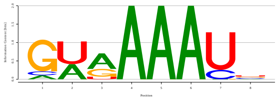	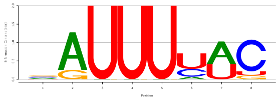	117 / 237	9691 / 19128	-
S7	Seeder	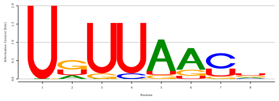	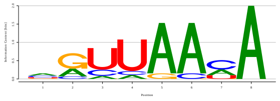	20 / 237	1982 / 19128	-

S8	Seeder	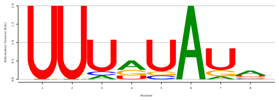	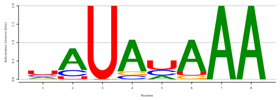	125 / 237	8116 / 19128	-
S9	Seeder	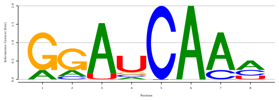	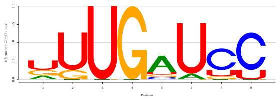	41 / 237	3654 / 19128	-
W1	Weeder	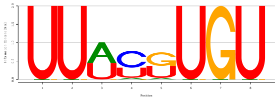	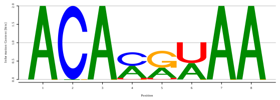	64 / 237	4258 / 19128	-
M1	MEME	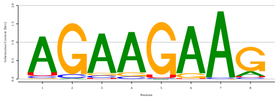	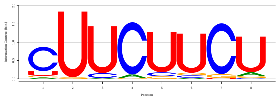	339 / 237	24856 / 19128	RNCMPT00078 (TRA2)
M2	MEME	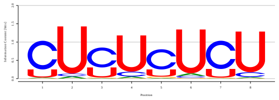	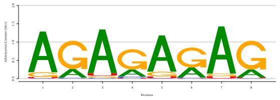	513 / 237	35216 / 19128	-
M3	MEME	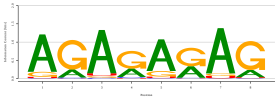	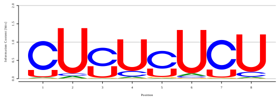	563 / 237	39309 / 19128	RNCMPT00089 (SRSF10)
M4	MEME	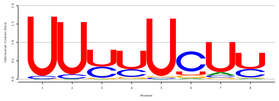	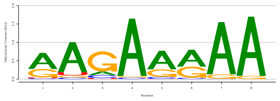	908 / 237	66925 / 19128	-
M5	MEME	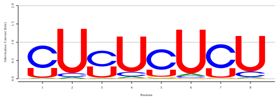	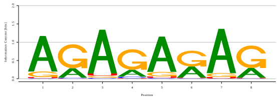	537 / 237	37670 / 19128	RNCMPT00215 (PCBP3)
M6	MEME	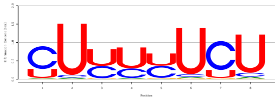	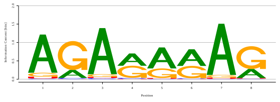	666 / 237	45998 / 19128	RNCMPT00215 (PCBP3)

[1]Name of the motif
[2]*de novo* discovery software that was used to locate the motif
[3]Forward sequence of the motif; Motif logo representing the occurence of a specific nucleotide at a respective position. The x-axis represents the position of a nucleotide and y-axis represents the amount of information in bits
[4]Reverse sequence of the motif; Motif logo representing the occurence of a specific nucleotide at a respective position.The x-axis represents the position of a nucleotide and y-axis represents the amount of information in bits
[5]Number of 5' UTRs containing the motif in the specific group. Note: Motif might occur more than once per sequence
[6]Number of 5' UTRs containing the motif in the whole Arabidopsis genome. Note: Motif might occur more than once per sequence
[7]Motif match in the database / literature

Supplemental Table 10: *De novo* motifs of length 8 found in the 5' UTRs of genes that belong to NYup group

		***De novo* motif**				
**Motif ID**[1]	**Software**[2]	**Forward**[3]	**Reverse**[4]	**Occurence in the group**[5]	**Occurence in the whole genome**[6]	**Possible function / Matches in RNA motif database - All species (Ray et al., 2013)**[7]
S10	Seeder	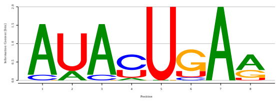	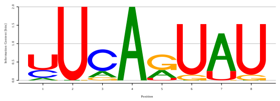	5 / 79	1562 / 19128	-
S11	Seeder	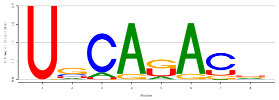	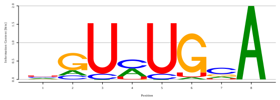	14 / 79	3453 / 19128	-
S12	Seeder	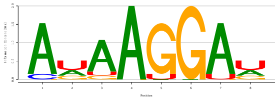	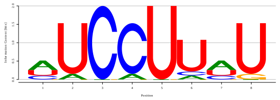	33 / 79	7955 / 19128	-
S13	Seeder	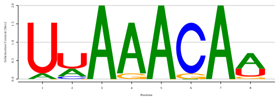	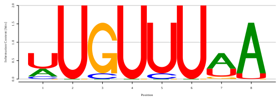	11 / 79	4407 / 19128	-
S14	Seeder	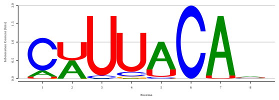	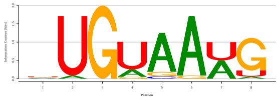	32 / 79	6802 / 19128	-
S15	Seeder	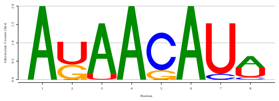	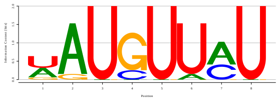	9 / 79	3160 / 19128	-
S16	Seeder	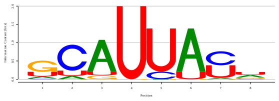	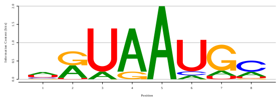	9 / 79	2853 / 19128	-
S18	Seeder	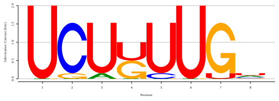	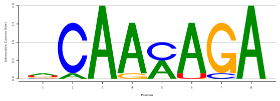	14 / 79	3788 / 19128	RNCMPT00217 (Tb_0217)
S19	Seeder	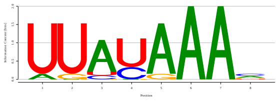	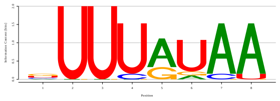	26 / 79	4502 / 19128	-
S1	Seeder	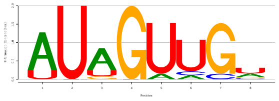	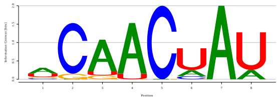	13 / 79	1905 / 19128	-

S20	Seeder	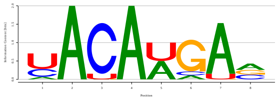	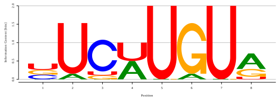	19 / 79	4257 / 19128	-
S21	Seeder	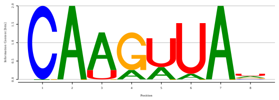	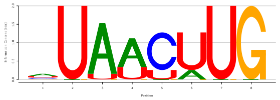	12 / 79	2628 / 19128	-
S22	Seeder	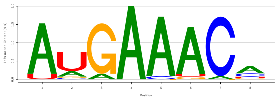	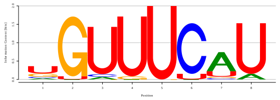	42 / 79	9392 / 19128	-
S23	Seeder	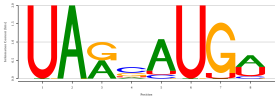	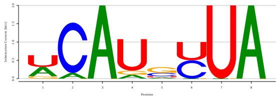	6 / 79	664 / 19128	-
S24	Seeder	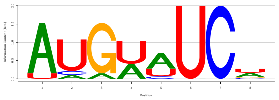	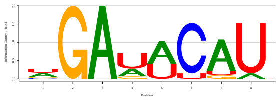	17 / 79	4529 / 19128	-
S25	Seeder	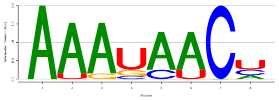	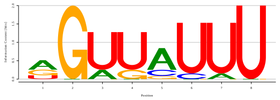	25 / 79	5208 / 19128	-
S26	Seeder	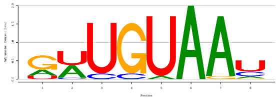	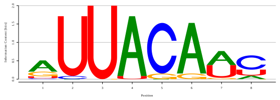	20 / 79	4528 / 19128	-
S27	Seeder	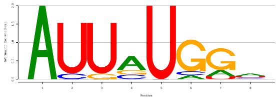	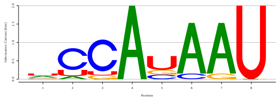	13 / 79	2639 / 19128	-
S28	Seeder	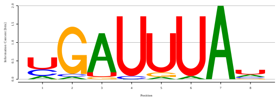	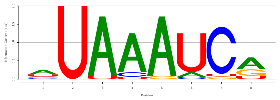	25 / 79	5072 / 19128	-
S29	Seeder	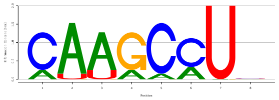	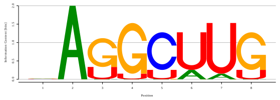	12 / 79	1251 / 19128	Matches to motif sequence TAGGGTTT, involved in translation regulation
S2	Seeder	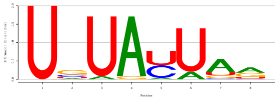	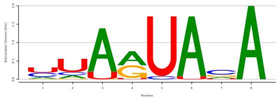	16 / 79	3216 / 19128	-

S30	Seeder	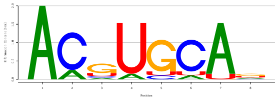	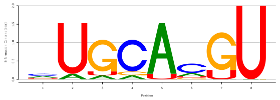	4 / 79	662 / 19128	-
S31	Seeder	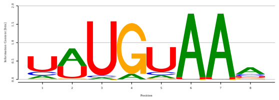	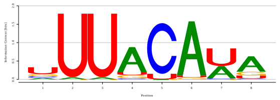	27 / 79	6254 / 19128	-
S32	Seeder	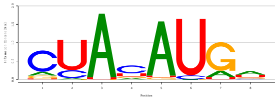	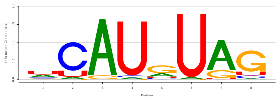	8 / 79	2779 / 19128	-
S33	Seeder	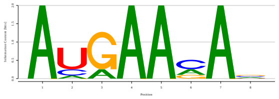	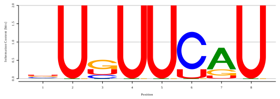	25 / 79	7134 / 19128	-
S34	Seeder	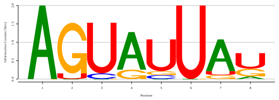	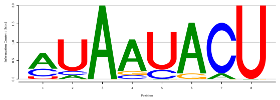	3 / 79	1283 / 19128	-
S35	Seeder	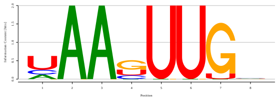	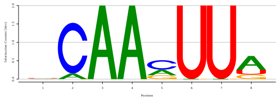	19 / 79	3574 / 19128	-
S36	Seeder	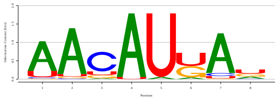	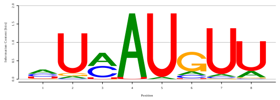	13 / 79	4087 / 19128	-
S37	Seeder	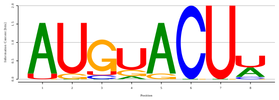	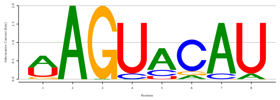	5 / 79	1179 / 19128	-
S38	Seeder	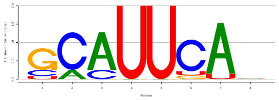	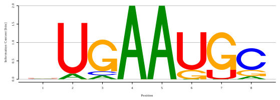	5 / 79	1732 / 19128	-
S39	Seeder	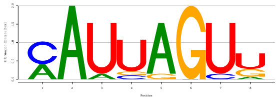	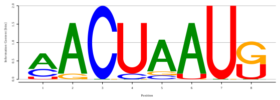	5 / 79	1515 / 19128	-
S3	Seeder	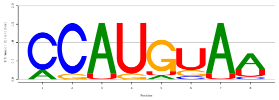	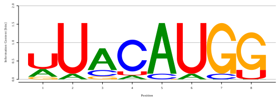	8 / 79	766 / 19128	-

S40	Seeder	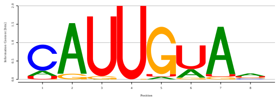	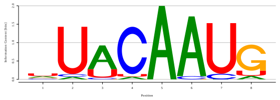	9 / 79	2973 / 19128	-
S4	Seeder	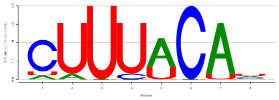	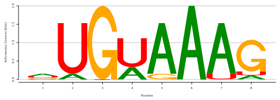	16 / 79	3045 / 19128	-
S5	Seeder	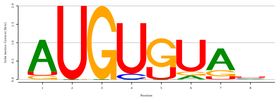	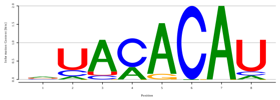	6 / 79	2736 / 19128	-
S6	Seeder	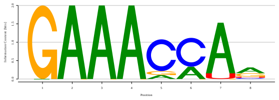	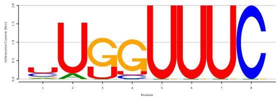	18 / 79	3911 / 19128	Matches to motif sequence TAGGGTTT, involved in translation regulation
S7	Seeder	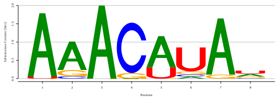	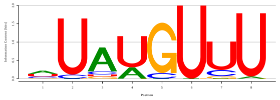	87 / 79	19231 / 19128	-
S8	Seeder	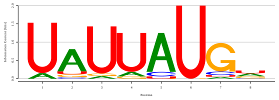	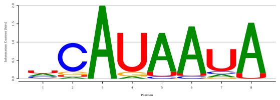	16 / 79	3623 / 19128	-
S9	Seeder	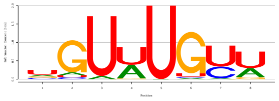	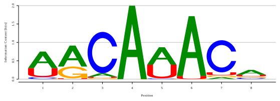	41 / 79	8515 / 19128	RNCMPT00258 (Tv_0258)
W1	Weeder	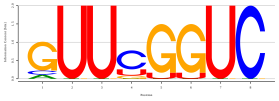	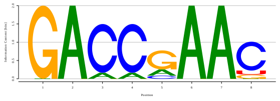	18 / 79	832 / 19128	-
M1	MEME	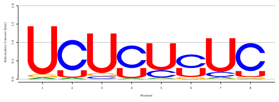	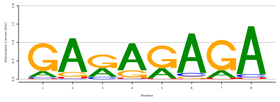	145 / 79	38301 / 19128	-
M2	MEME	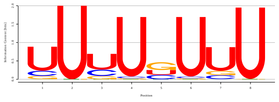	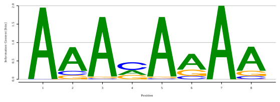	308 / 79	60633 / 19128	RNCMPT00086(ZC3H14), RNCMPT00112(HuR), RNCMPT00117(HuR), RNCMPT00121(ELAV)
M3	MEME	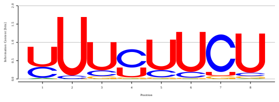	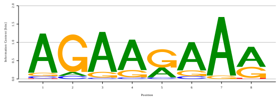	190 / 79	44507 / 19128	RNCMPT00079(U2AF2), RNCMPT00080(U2AF50), RNCMPT00165(TIA1)

M4	MEME	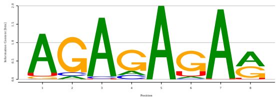	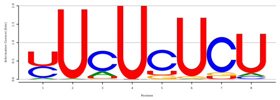	182 / 79	44589 / 19128	-
M5	MEME	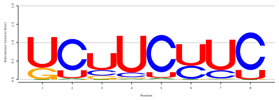	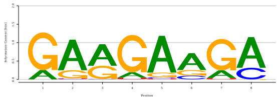	136 / 79	37497 / 19128	-
M6	MEME	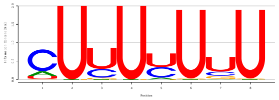	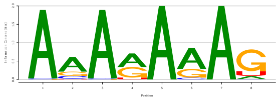	196 / 79	41592 / 19128	RNCMPT00119 (SXL), RNCMPT00005 (TIAR-3), RNCMPT00256 (TIAR-1), RNCMPT00158 (CPEB4), RNCMPT00274 (HuR), RNCMPT00012 (CPEB2)
M7	MEME	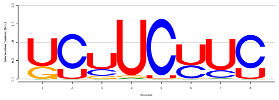	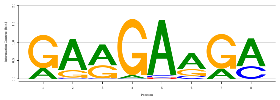	143 / 79	40533 / 19128	-
M8	MEME	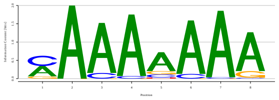	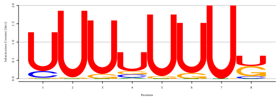	233 / 79	46703 / 19128	RNCMPT00043 (PABPC4)

[1]Name of the motif
[2]*de novo* discovery software that was used to locate the motif
[3]Forward sequence of the motif; Motif logo representing the occurence of a specific nucleotide at a respective position. The x-axis represents the position of a nucleotide and y-axis represents the amount of information in bits
[4]Reverse sequence of the motif; Motif logo representing the occurence of a specific nucleotide at a respective position.The x-axis represents the position of a nucleotide and y-axis represents the amount of information in bits
[5]Number of 5' UTRs containing the motif in the specific group. Note: Motif might occur more than once per sequence
[6]Number of 5' UTRs containing the motif in the whole Arabidopsis genome. Note: Motif might occur more than once per sequence
[7]Motif match in the database / literature

Supplemental Table 11: *De novo* motifs of length 8 found in the 5' UTRs of genes that belong to YNdown group

			***De novo* motif**			
**Motif ID**[1]	**Software**[2]	**Forward**[3]	**Reverse**[4]	**Occurence in the group**[5]	**Occurence in the whole genome**[6]	**Possible function / Matches in RNA motif database - All species (Ray et al., 2013)**[7]
S10	Seeder	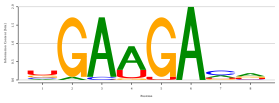	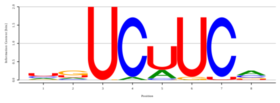	96 / 53	31464 / 19128	-
S11	Seeder	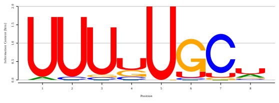	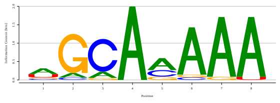	44 / 53	12313 / 19128	-
S12	Seeder	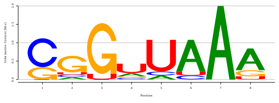	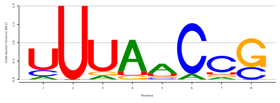	14 / 53	2238 / 19128	-
S13	Seeder	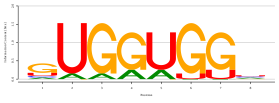	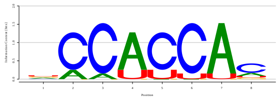	7 / 53	1944 / 19128	-
S14	Seeder	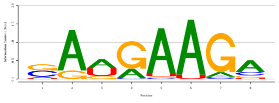	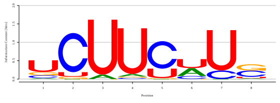	124 / 53	42009 / 19128	-
S15	Seeder	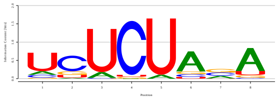	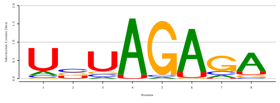	47 / 53	11488 / 19128	-
S16	Seeder	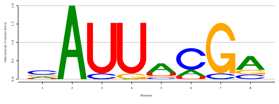	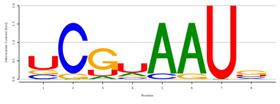	7 / 53	2047 / 19128	-
S17	Seeder	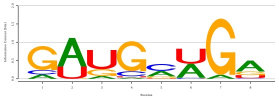	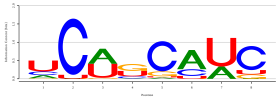	70 / 53	21552 / 19128	-
S18	Seeder	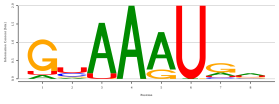	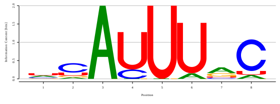	17 / 53	3951 / 19128	-
S19	Seeder	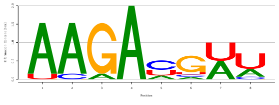	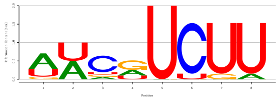	42 / 53	14140 / 19128	-

S1	Seeder	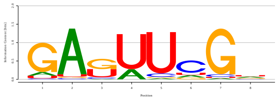	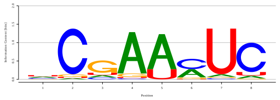	20 / 53	5581 / 19128	-
S20	Seeder	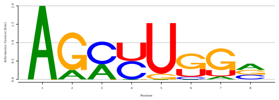	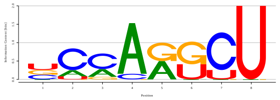	10 / 53	2094 / 19128	-
S21	Seeder	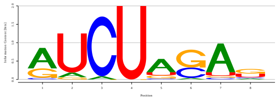	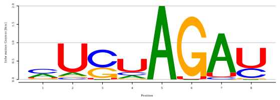	31 / 53	6396 / 19128	-
S22	Seeder	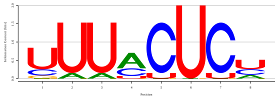	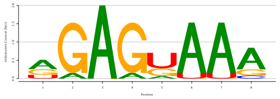	26 / 53	8139 / 19128	-
S23	Seeder	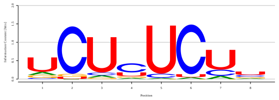	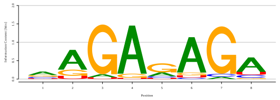	126 / 53	42638 / 19128	RNCMPT00220 (Tb_0220)
S24	Seeder	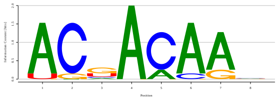	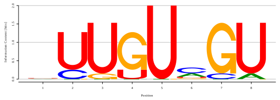	20 / 53	4180 / 19128	-
S25	Seeder	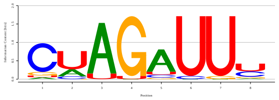	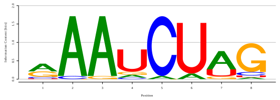	42 / 53	6270 / 19128	-
S26	Seeder	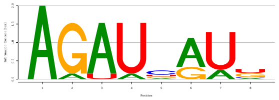	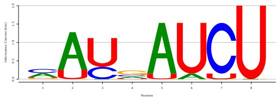	21 / 53	4364 / 19128	-
S27	Seeder	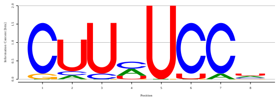	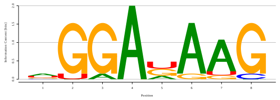	23 / 53	6138 / 19128	-
S28	Seeder	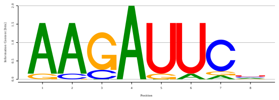	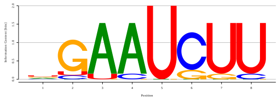	21 / 53	3654 / 19128	-
S29	Seeder	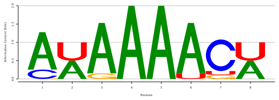	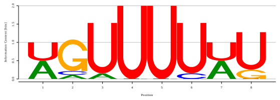	36 / 53	9895 / 19128	-

S2	Seeder	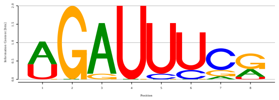	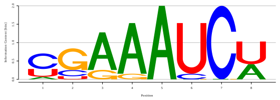	30 / 53	4470 / 19128	-
S30	Seeder	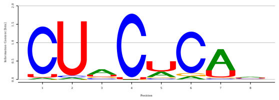	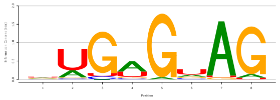	51 / 53	14542 / 19128	-
S31	Seeder	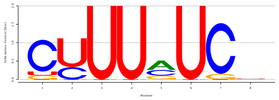	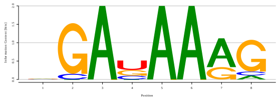	25 / 53	6856 / 19128	-
S32	Seeder	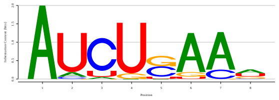	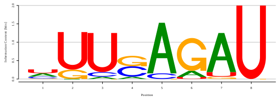	57 / 53	16723 / 19128	-
S33	Seeder	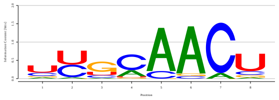	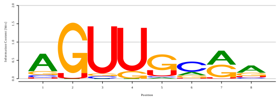	14 / 53	5614 / 19128	-
S34	Seeder	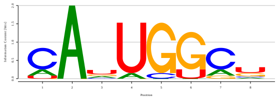	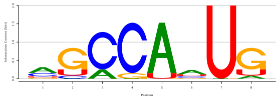	9 / 53	1918 / 19128	-
S35	Seeder	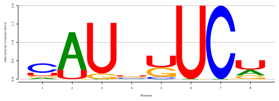	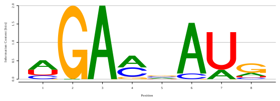	47 / 53	14497 / 19128	-
S36	Seeder	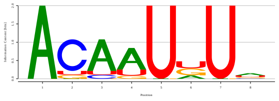	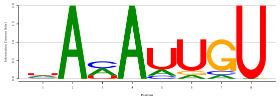	11 / 53	2317 / 19128	-
S37	Seeder	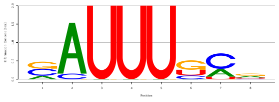	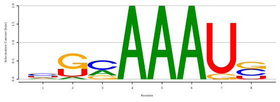	21 / 53	4575 / 19128	-
S38	Seeder	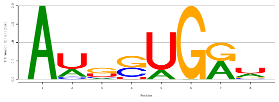	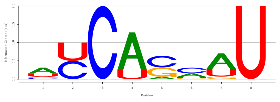	31 / 53	8057 / 19128	-
S39	Seeder	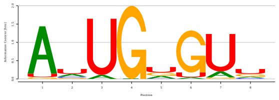	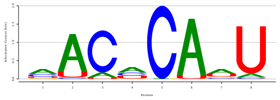	47 / 53	10071 / 19128	-

S3	Seeder	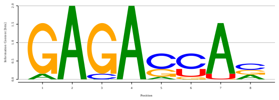	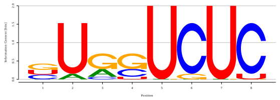	19 / 53	2973 / 19128	-
S40	Seeder	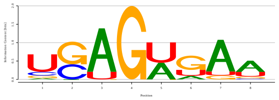	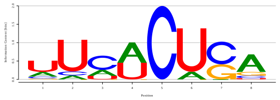	87 / 53	25809 / 19128	-
S41	Seeder	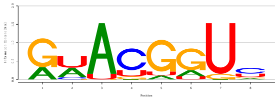	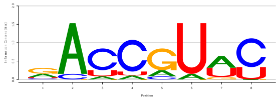	6 / 53	736 / 19128	-
S42	Seeder	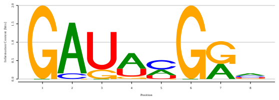	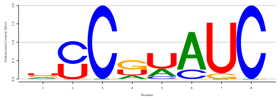	17 / 53	4850 / 19128	RNCMPT00050 (RBM3)
S43	Seeder	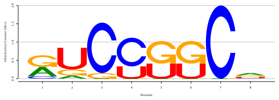	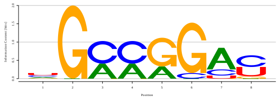	9 / 53	2346 / 19128	-
S44	Seeder	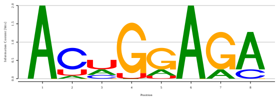	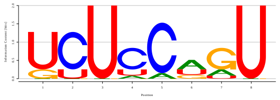	19 / 53	4835 / 19128	-
S45	Seeder	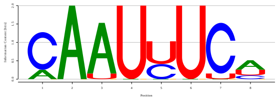	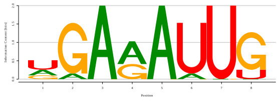	24 / 53	4021 / 19128	-
S46	Seeder	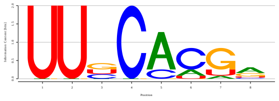	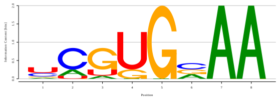	12 / 53	2452 / 19128	RNCMPT00249 (Pr_0249), RNCMPT00071 (SNRPA)
S47	Seeder	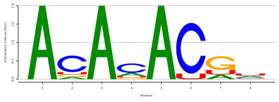	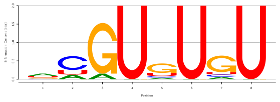	14 / 53	3266 / 19128	RNCMPT00069 (SM), RNCMPT00178 (HNRPLL)
S48	Seeder	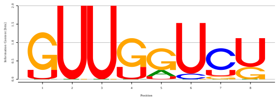	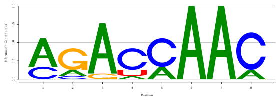	12 / 53	1824 / 19128	-
S49	Seeder	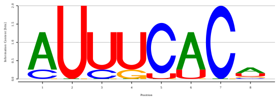	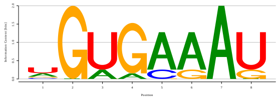	25 / 53	6172 / 19128	RNCMPT00145 (SNF)

S4	Seeder	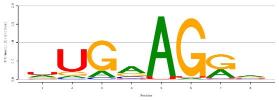	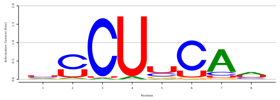	84 / 53	23947 / 19128	-
S50	Seeder	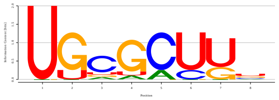	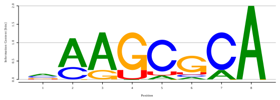	11 / 53	2114 / 19128	Matches to motif sequence TAGGGTTT, involved in translation regulation
S5	Seeder	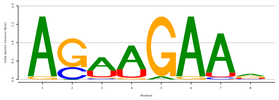	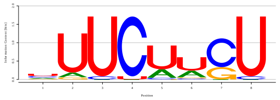	113 / 53	38165 / 19128	-
S6	Seeder	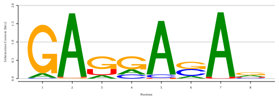	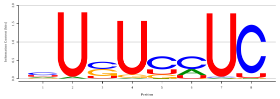	61 / 53	17479 / 19128	-
S7	Seeder	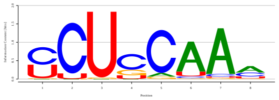	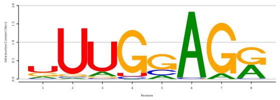	25 / 53	6896 / 19128	-
S8	Seeder	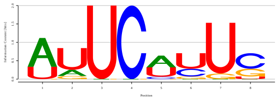	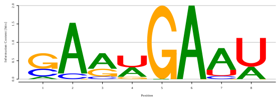	52 / 53	12452 / 19128	-
S9	Seeder	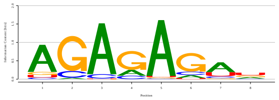	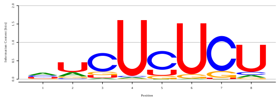	150 / 53	48878 / 19128	-
W1	Weeder	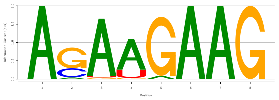	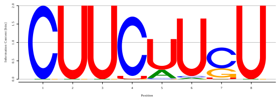	38 / 53	10355 / 19128	RNCMPT00078 (TRA2)
M1	MEME	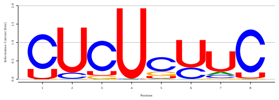	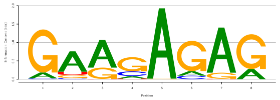	69 / 53	20644 / 19128	RNCMPT00220 (Tb_0220)
M2	MEME	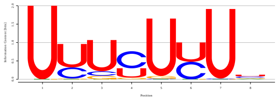	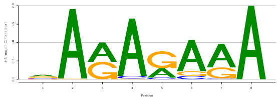	155 / 53	52082 / 19128	-
M3	MEME	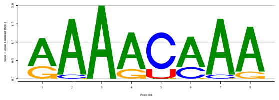	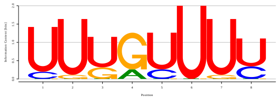	53 / 53	12815 / 19128	Matches to motif sequence TAGGGTTT, involved in translation regulation; RNCMPT00255 (Lm_0255)

M4	MEME	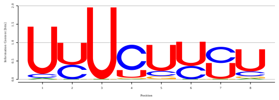	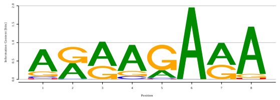	165 / 53	55595 / 19128	-
M5	MEME	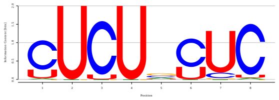	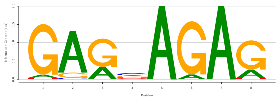	47 / 53	12280 / 19128	-
M6	MEME	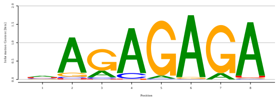	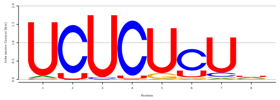	152 / 53	49835 / 19128	RNCMPT00089 (SRSF10)
M7	MEME	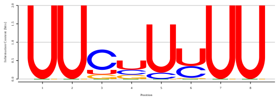	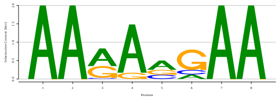	57 / 53	16492 / 19128	-
M8	MEME	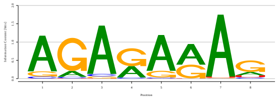	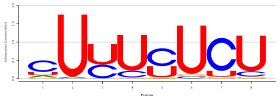	170 / 53	55854 / 19128	RNCMPT00089 (SRSF10), RNCMPT00019 (SRSF10), RNCMPT00090 (SRSF10)

[1]Name of the motif
[2]*de novo* discovery software that was used to locate the motif
[3]Forward sequence of the motif; Motif logo representing the occurence of a specific nucleotide at a respective position. The x-axis represents the position of a nucleotide and y-axis represents the amount of information in bits
[4]Reverse sequence of the motif; Motif logo representing the occurence of a specific nucleotide at a respective position.The x-axis represents the position of a nucleotide and y-axis represents the amount of information in bits
[5]Number of 5' UTRs containing the motif in the specific group. Note: Motif might occur more than once per sequence
[6]Number of 5' UTRs containing the motif in the whole Arabidopsis genome. Note: Motif might occur more than once per sequence
[7]Motif match in the database / literature

Supplemental Table 12: *De novo* motifs of length 8 found in the 5' UTRs of genes that belong to YNup group

		***De novo* motif**				
**Motif ID[1]**	**Software[2]**	**Forward[3]**	**Reverse[4]**	**Occurence in the group[5]**	**Occurence in the whole genome[6]**	**Possible function / Matches in RNA motif database - All species (Ray et al., 2013)[7]**
S10	Seeder	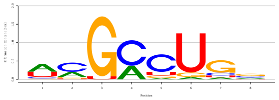	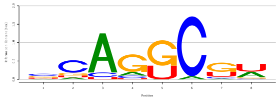	20 / 58	3002 / 19128	-
S11	Seeder	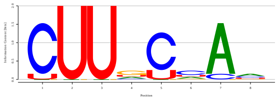	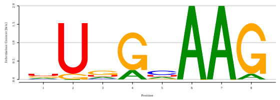	9 / 58	1720 / 19128	-
S12	Seeder	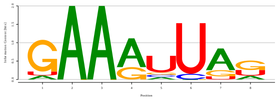	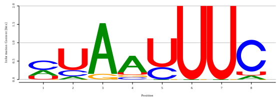	22 / 58	6173 / 19128	-
S13	Seeder	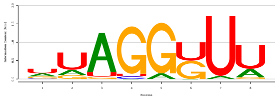	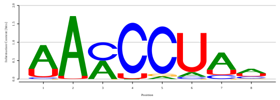	26 / 58	9564 / 19128	Matches to motif sequence TAGGGTTT, involved in translation regulation
S14	Seeder	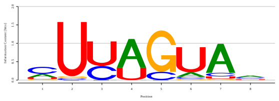	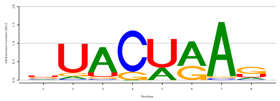	19 / 58	4220 / 19128	-
S15	Seeder	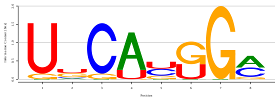	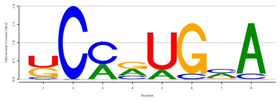	11 / 58	1971 / 19128	-
S16	Seeder	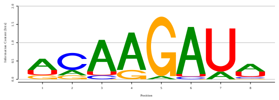	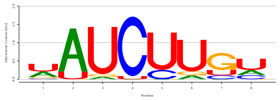	36 / 58	9671 / 19128	-
S17	Seeder	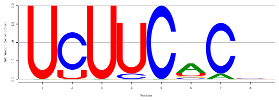	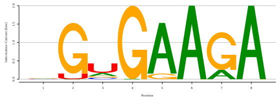	40 / 58	9715 / 19128	-
S18	Seeder	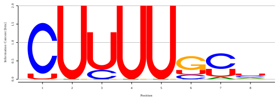	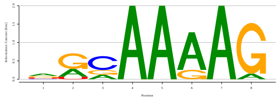	21 / 58	6929 / 19128	-
S19	Seeder	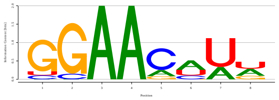	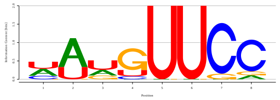	18 / 58	4889 / 19128	-

S1	Seeder	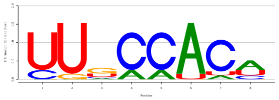	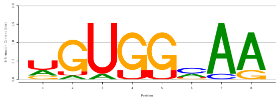	12 / 58	2657 / 19128	-
S20	Seeder	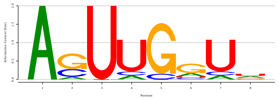	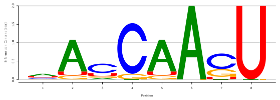	10 / 58	3483 / 19128	-
S21	Seeder	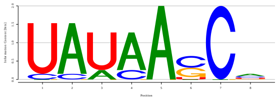	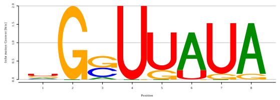	16 / 58	4025 / 19128	-
S22	Seeder	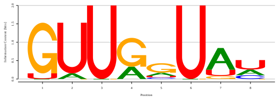	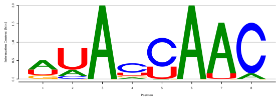	11 / 58	1654 / 19128	-
S23	Seeder	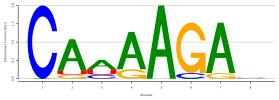	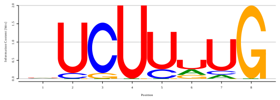	39 / 58	10098 / 19128	-
S24	Seeder	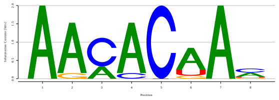	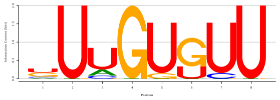	24 / 58	5632 / 19128	RNCMPT00091 (HNRNPL)
S25	Seeder	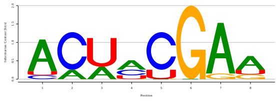	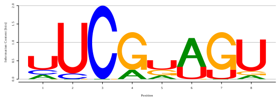	13 / 58	3206 / 19128	-
S26	Seeder	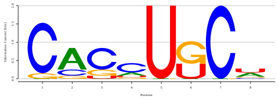	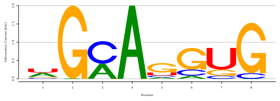	9 / 58	1936 / 19128	-
S27	Seeder	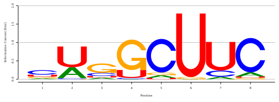	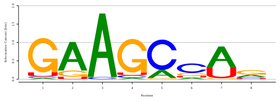	19 / 58	3796 / 19128	-
S28	Seeder	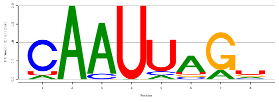	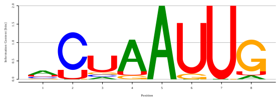	10 / 58	2296 / 19128	-
S29	Seeder	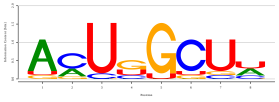	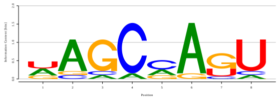	12 / 58	2020 / 19128	-

S2	Seeder	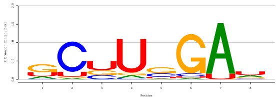	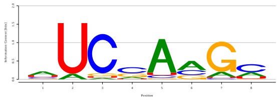	40 / 58	9892 / 19128	-
S30	Seeder	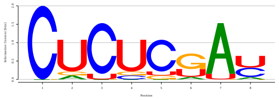	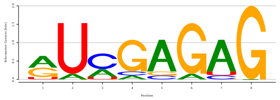	13 / 58	3476 / 19128	-
S31	Seeder	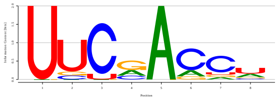	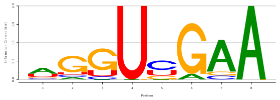	11 / 58	2455 / 19128	-
S32	Seeder	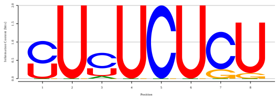	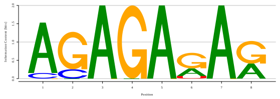	85 / 58	20125 / 19128	-
S33	Seeder	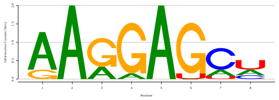	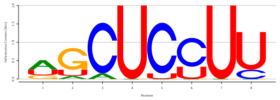	20 / 58	5012 / 19128	-
S34	Seeder	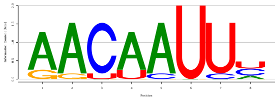	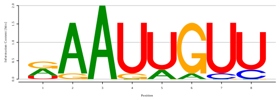	14 / 58	2508 / 19128	-
S35	Seeder	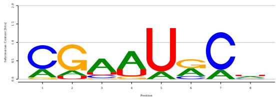	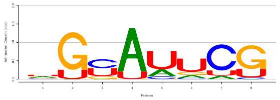	19 / 58	5417 / 19128	-
S36	Seeder	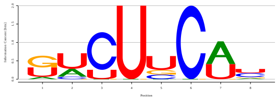	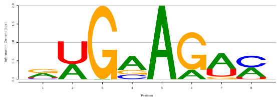	29 / 58	11125 / 19128	-
S37	Seeder	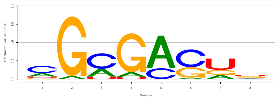	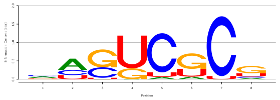	19 / 58	4872 / 19128	-
S38	Seeder	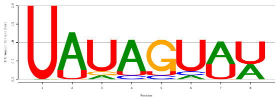	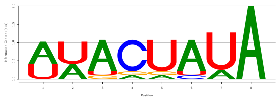	13 / 58	2237 / 19128	-
S3	Seeder	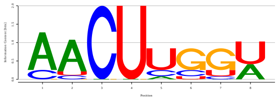	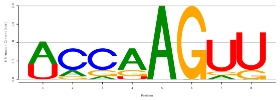	17 / 58	2654 / 19128	-

S40	Seeder	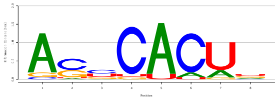	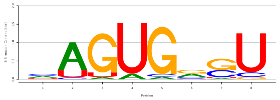	19 / 58	4652 / 19128	-
S41	Seeder	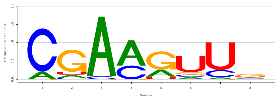	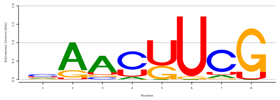	30 / 58	7594 / 19128	-
S42	Seeder	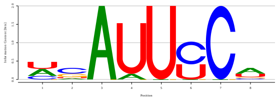	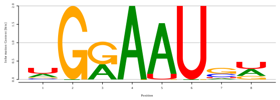	19 / 58	6041 / 19128	-
S4	Seeder	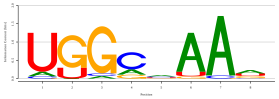	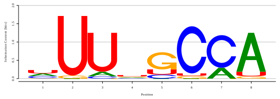	27 / 58	7959 / 19128	-
S5	Seeder	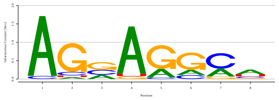	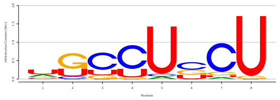	19 / 58	6269 / 19128	RNCMPT00059 (REF2)
S6	Seeder	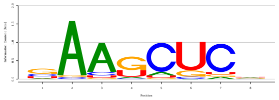	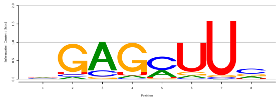	29 / 58	8464 / 19128	-
S7	Seeder	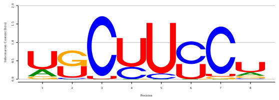	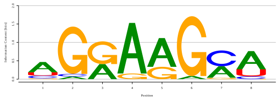	33 / 58	13465 / 19128	-
S8	Seeder	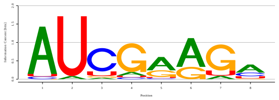	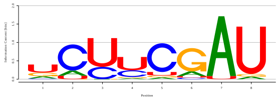	40 / 58	13342 / 19128	-
W1	Weeder	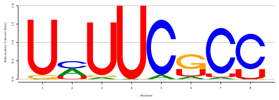	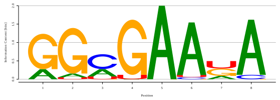	15 / 58	2801 / 19128	-
M7	MEME	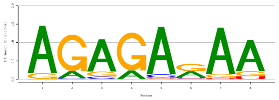	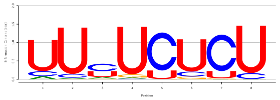	14 / 58	2114 / 19128	RNCMPT00089 (SRSF10)
M1	MEME	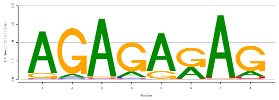	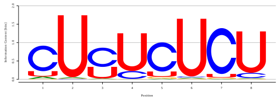	134 / 58	37904 / 19128	RNCMPT00089 (SRSF10)

M2	MEME	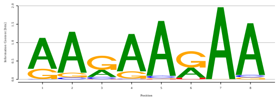	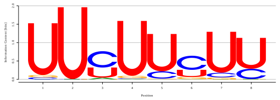	155 / 58	52185 / 19128	-
M3	MEME	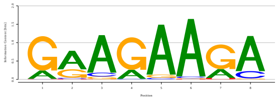	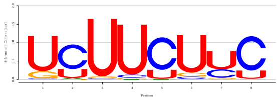	99 / 58	35886 / 19128	RNCMPT00078 (TRA2)
M4	MEME	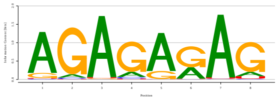	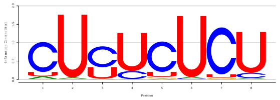	159 / 58	48468 / 19128	RNCMPT00089 (SRSF10)
M5	MEME	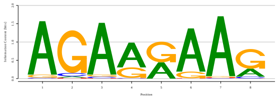	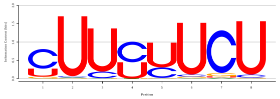	184 / 58	62806 / 19128	-
M6	MEME	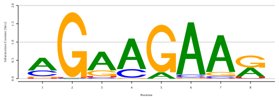	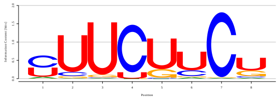	165 / 58	49464 / 19128	-

[1]Name of the motif
[2]*de novo* discovery software that was used to locate the motif
[3]Forward sequence of the motif; Motif logo representing the occurence of a specific nucleotide at a respective position. The x-axis represents the position of a nucleotide and y-axis represents the amount of information in bits
[4]Reverse sequence of the motif; Motif logo representing the occurence of a specific nucleotide at a respective position.The x-axis represents the position of a nucleotide and y-axis represents the amount of information in bits
[5]Number of 5' UTRs containing the motif in the specific group. Note: Motif might occur more than once per sequence
[6]Number of 5' UTRs containing the motif in the whole Arabidopsis genome. Note: Motif might occur more than once per sequence
[7]Motif match in the database / literature

Supplemental Table 13: *De novo* motifs of length 8 found in the 5' UTRs of genes that belong to YYdown group

			***De novo* motif**			
**Motif ID**[1]	**Software**[2]	**Forward**[3]	**Reverse**[4]	**Occurence in the group**[5]	**Occurence in the whole genome**[6]	**Possible function / Matches in RNA motif database - All species (Ray et al., 2013)**[7]
S0	Seeder	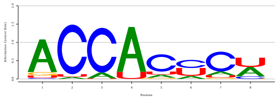	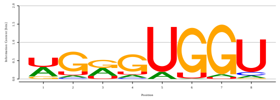	13 / 22	4134 / 19128	-
S10	Seeder	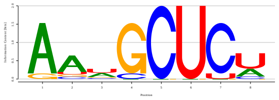	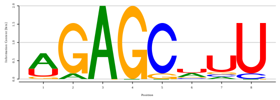	17 / 22	4152 / 19128	-
S11	Seeder	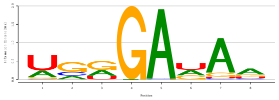	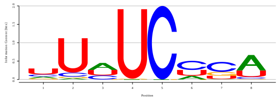	42 / 22	39501 / 19128	-
S13	Seeder	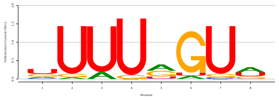	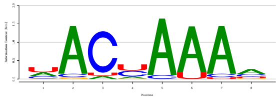	25 / 22	14796 / 19128	-
S15	Seeder	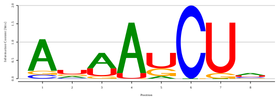	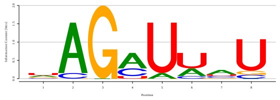	24 / 22	16745 / 19128	-
S16	Seeder	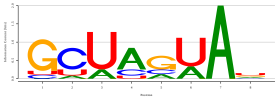	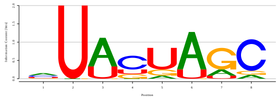	7 / 22	1970 / 19128	-
S17	Seeder	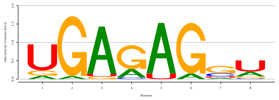	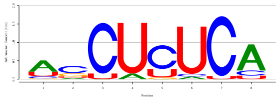	21 / 22	10278 / 19128	-
S18	Seeder	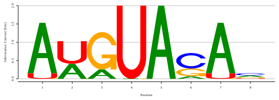	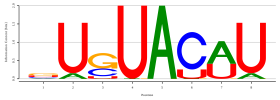	9 / 22	1717 / 19128	-
S19	Seeder	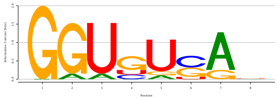	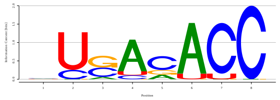	11 / 22	2974 / 19128	-
S1	Seeder	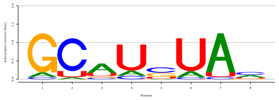	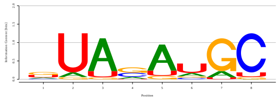	9 / 22	2539 / 19128	-

S20	Seeder	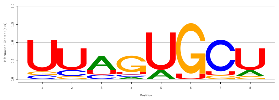	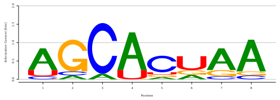	8 / 22	2451 / 19128	-
S21	Seeder	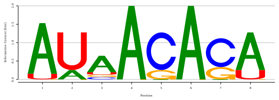	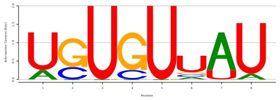	11 / 22	4494 / 19128	-
S22	Seeder	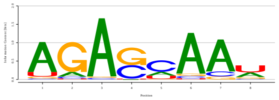	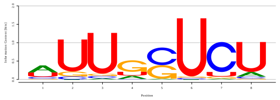	26 / 22	18892 / 19128	-
S23	Seeder	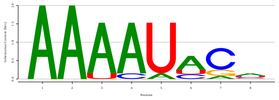	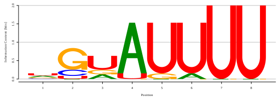	13 / 22	9649 / 19128	-
S24	Seeder	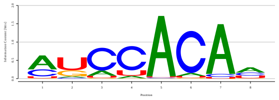	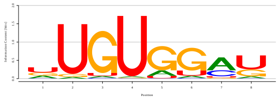	22 / 22	10516 / 19128	-
S25	Seeder	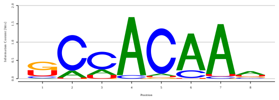	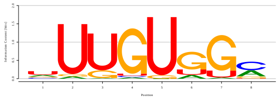	12 / 22	6362 / 19128	-
S26	Seeder	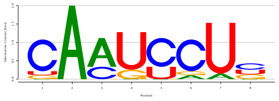	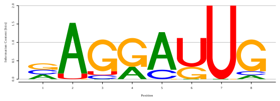	10 / 22	2723 / 19128	-
S27	Seeder	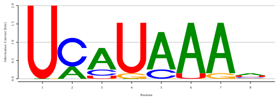	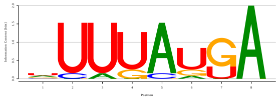	13 / 22	6975 / 19128	-
S28	Seeder	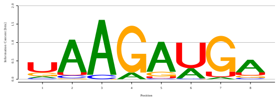	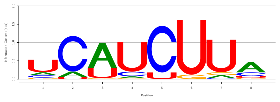	40 / 22	25074 / 19128	-
S29	Seeder	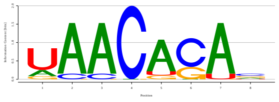	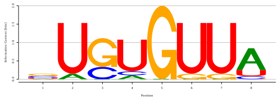	13 / 22	6007 / 19128	-
S31	Seeder	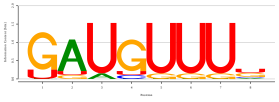	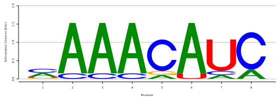	9 / 22	2220 / 19128	Matches to motif sequence TAGGGTTT, involved in translation regulation

S32	Seeder	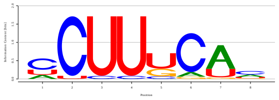	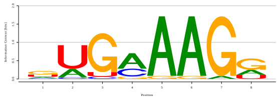	16 / 22	9203 / 19128	RNCMPT00239 (PCBP1)
S33	Seeder	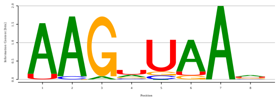	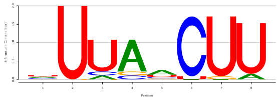	26 / 22	18392 / 19128	-
S34	Seeder	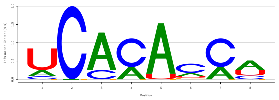	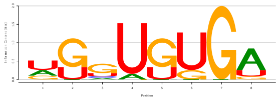	16 / 22	6289 / 19128	-
S35	Seeder	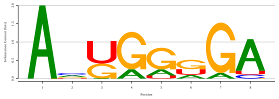	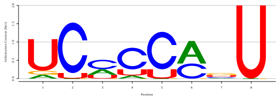	12 / 22	3171 / 19128	-
S36	Seeder	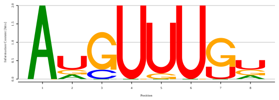	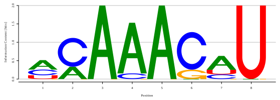	11 / 22	5218 / 19128	-
S37	Seeder	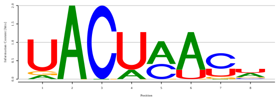	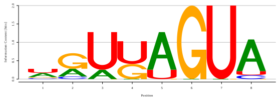	17 / 22	3439 / 19128	-
S38	Seeder	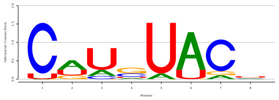	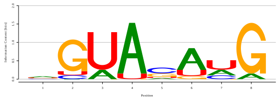	11 / 22	4365 / 19128	-
S39	Seeder	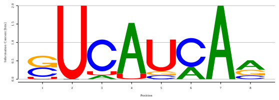	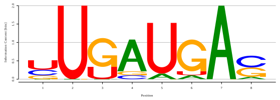	10 / 22	3460 / 19128	-
S3	Seeder	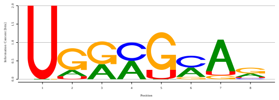	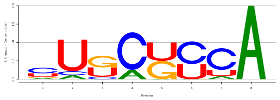	11 / 22	4559 / 19128	-
S40	Seeder	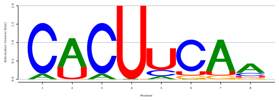	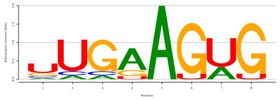	9 / 22	3664 / 19128	-
S41	Seeder	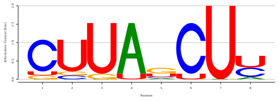	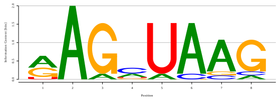	6 / 22	2344 / 19128	-

S42	Seeder	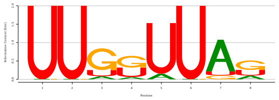	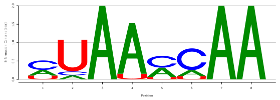	8 / 22	2437 / 19128	-
S43	Seeder	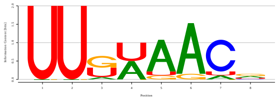	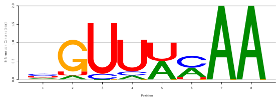	6 / 22	2756 / 19128	-
S44	Seeder	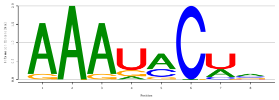	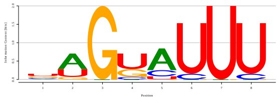	19 / 22	10360 / 19128	Matches to motif sequence TAGGGTTT, involved in translation regulation
S45	Seeder	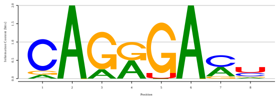	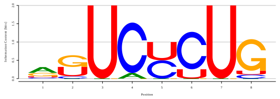	7 / 22	3029 / 19128	-
S47	Seeder	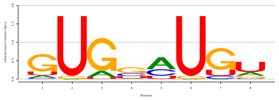	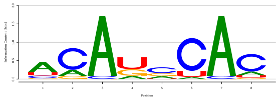	10 / 22	3551 / 19128	-
S48	Seeder	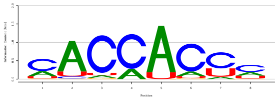	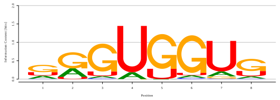	12 / 22	2761 / 19128	-
S49	Seeder	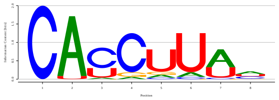	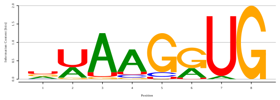	16 / 22	4400 / 19128	-
S4	Seeder	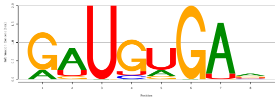	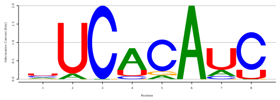	13 / 22	4352 / 19128	-
S5	Seeder	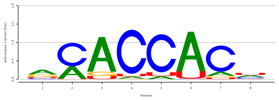	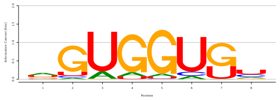	21 / 22	6168 / 19128	-
S6	Seeder	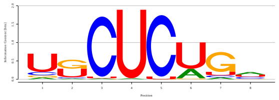	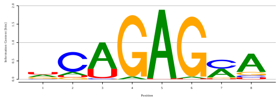	23 / 22	11719 / 19128	-
S7	Seeder	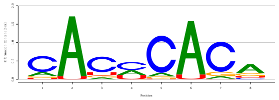	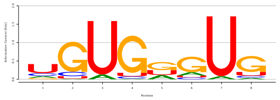	12 / 22	4853 / 19128	NCMPT00069 (SM), RNCMPT00178 (HNRPLL), RNCMPT00027 (HNRNPL)

S8	Seeder	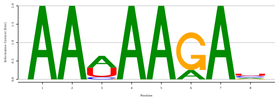	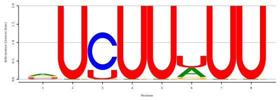	20 / 22	9777 / 19128	-
S9	Seeder	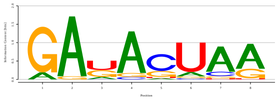	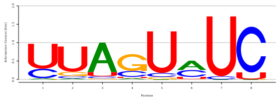	15 / 22	7304 / 19128	-
W1	Weeder	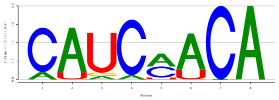	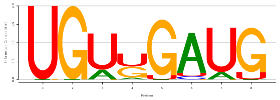	14 / 22	3157 / 19128	-
M1	MEME	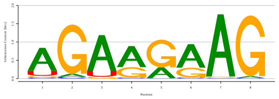	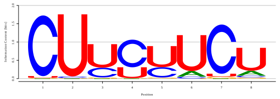	59 / 22	42584 / 19128	RNCMPT00078 (TRA2)
M2	MEME	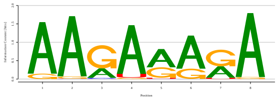	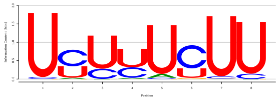	95 / 22	67585 / 19128	-
M3	MEME	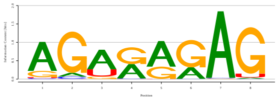	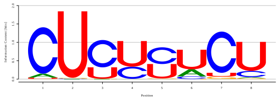	63 / 22	45269 / 19128	-
M4	MEME	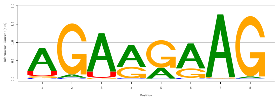	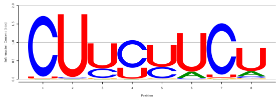	65 / 22	42790 / 19128	RNCMPT00078 (TRA2)

[1]Name of the motif
[2]*de novo* discovery software that was used to locate the motif
[3]Forward sequence of the motif; Motif logo representing the occurence of a specific nucleotide at a respective position. The x-axis represents the position of a nucleotide and y-axis represents the amount of information in bits
[4]Reverse sequence of the motif; Motif logo representing the occurence of a specific nucleotide at a respective position.The x-axis represents the position of a nucleotide and y-axis represents the amount of information in bits
[5]Number of 5' UTRs containing the motif in the specific group. Note: Motif might occur more than once per sequence
[6]Number of 5' UTRs containing the motif in the whole Arabidopsis genome. Note: Motif might occur more than once per sequence
[7]Motif match in the database / literature

Supplemental Table 14: *De novo* motifs of length 8 found in the 5' UTRs of genes that belong to YYup group

***De novo* motif**						
**Motif ID**[1]	**Software**[2]	**Forward**[3]	**Reverse**[4]	**Occurence in the group**[5]	**Occurence in the whole genome**[6]	**Possible function / Matches in RNA motif database - All species (Ray et al., 2013)**[7]
W1	Weeder	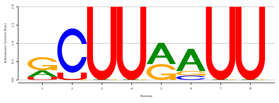	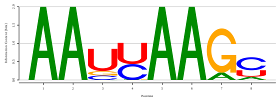	6 / 6	1886 / 19128	-

[1]Name of the motif

[2]*de novo* discovery software that was used to locate the motif

[3]Forward sequence of the motif; Motif logo representing the occurence of a specific nucleotide at a respective position. The x-axis represents the position of a nucleotide and y-axis represents the amount of information in bits

[4]Reverse sequence of the motif; Motif logo representing the occurence of a specific nucleotide at a respective position.The x-axis represents the position of a nucleotide and y-axis represents the amount of information in bits

[5]Number of 5' UTRs containing the motif in the specific group. Note: Motif might occur more than once per sequence

[6]Number of 5' UTRs containing the motif in the whole Arabidopsis genome. Note: Motif might occur more than once per sequence

[7]Motif match in the database / literature
